# Discovery and Genetic Code Expansion of a Polyethylene Terephthalate (PET) Hydrolase from the Human Saliva Metagenome for the Degradation and Bio-Functionalization of PET

**DOI:** 10.1002/anie.202203061

**Published:** 2022-06-21

**Authors:** Bhumrapee Eiamthong, Piyachat Meesawat, Thanakrit Wongsatit, Jariya Jitdee, Raweewan Sangsri, Maturada Patchsung, Kanokpol Aphicho, Surased Suraritdechachai, Nicolas Huguenin-Dezot, Shan Tang, Wipa Suginta, Boonchoat Paosawatyanyong, M. Madan Babu, Jason W. Chin, Danaya Pakotiprapha, Worawan Bhanthumnavin, Chayasith Uttamapinant

**Affiliations:** School of Biomolecular Science and Engineering, Vidyasirimedhi Institute of Science and Technology (VISTEC), Rayong 21210 (Thailand); Department of Chemistry, Faculty of Science, Chulalongkorn University, Bangkok 10330 (Thailand); Department of Biochemistry and Center for Excellence in Protein and Enzyme Technology, Faculty of Science, Mahidol University, Bangkok 10400 (Thailand); School of Biomolecular Science and Engineering, Vidyasirimedhi Institute of Science and Technology (VISTEC), Rayong 21210 (Thailand); Medical Research Council Laboratory of Molecular Biology, Cambridge CB2 0QH (UK); School of Biomolecular Science and Engineering, Vidyasirimedhi Institute of Science and Technology (VISTEC), Rayong 21210 (Thailand); Department of Physics, Faculty of Science, Chulalongkorn University, Bangkok 10330 (Thailand); Department of Structural Biology and Center of Excellence for Data Driven Discovery, St. Jude Children’s Research Hospital, Memphis, TN 38105 (USA); Medical Research Council Laboratory of Molecular Biology, Cambridge CB2 0QH (UK); Department of Biochemistry and Center for Excellence in Protein and Enzyme Technology, Faculty of Science, Mahidol University, Bangkok 10400 (Thailand); Department of Chemistry, Faculty of Science, Chulalongkorn University, Bangkok 10330 (Thailand); School of Biomolecular Science and Engineering, Vidyasirimedhi Institute of Science and Technology (VISTEC), Rayong 21210 (Thailand)

**Keywords:** Bioinformatics, Degradation, Genetic Code Expansion, Hydrolases, Plastics

## Abstract

We report a bioinformatic workflow and subsequent discovery of a new polyethylene terephthalate (PET) hydrolase, which we named MG8, from the human saliva metagenome. MG8 has robust PET plastic degradation activities under different temperature and salinity conditions, outperforming several naturally occurring and engineered hydrolases in degrading PET. Moreover, we genetically encoded 2,3-diaminopropionic acid (DAP) in place of the catalytic serine residue of MG8, thereby converting a PET hydrolase into a covalent binder for bio-functionalization of PET. We show that MG8(DAP), in conjunction with a split green fluorescent protein system, can be used to attach protein cargos to PET as well as other polyester plastics. The discovery of a highly active PET hydrolase from the human metagenome—currently an underexplored resource for industrial enzyme discovery—as well as the repurposing of such an enzyme into a plastic functionalization tool, should facilitate ongoing efforts to degrade and maximize reusability of PET.

## Introduction

Enzymatic degradation of polyethylene terephthalate (PET) with hydrolases could enable sustainable recycling of PET and be linked to downstream green processes to convert PET monomers into value-added chemicals.^[1]^ Thermostable PET hydrolases (identified from thermophilic hosts or engineered to be so) can hydrolyze PET under high temperatures, which facilitate degradation of polymer chains. These enzymes can be coupled to reactor systems for industrialscale plastic degradation.^[2]^ PET hydrolases identified from mesophilic hosts, on the other hand, can function at lower temperatures^[3]^ which can save process operation cost, and may facilitate strain-based degradation of PET waste in a facility with appropriate biosafety measures.^[4]^ Mesophilic and thermophilic PET hydrolases are not mutually exclusive; the former group can be engineered to achieve higher thermal stability, albeit with a more limited range of operational temperatures and conditions.

Almost all useful PET hydrolases were isolated from environmental microbial communities and subsequently cultured for strain isolation and gene/protein characterization. *Ideonella sakeiensis* PET hydrolase (*Is*PETase) identified from environmental samples at PET recycling facilities is a key recent example.^[3, 5]^ As 99% of bacteria cannot be cultured,^[6]^ metagenomic data from suitable environments in which PET hydrolases could evolve are rich alternative resources for new enzyme discovery. As successful examples, a highly used PET hydrolase, leaf and branch compost cutinase^[7]^ (LCC) and recently discovered PHL-7^[8]^ were found from metagenomic DNA extracts from composts. Due to limitations in current PET hydrolases, we were motivated to search for highly active PET hydrolases that can withstand changes in temperature, salinity, and pH, and became particularly interested in two microbiome environments: marine systems, and human digestive systems. Marine systems have fluctuating environmental conditions, and the concentrated presence of carbon sources in the form of solid plastics may drive evolution of bacteria to utilize such resources. Recent metagenomic surveys suggested high prevalence of PET hydrolases in marine microbial communities.^[9]^ A PET hydrolase, PE-H from a marine bacterium *Pseudomonas aestusnigri*,^[10]^ as well as several PET hydrolases from marine metagenomes^[11]^ have been biochemically characterized.

While the microbiomes of human digestive systems—typically collected from feces, rumen fluids, and saliva—reside in isothermal conditions, they experience shifts in salinity and pH, particularly due to diet, and must maintain activities of their catabolic enzymes^[12]^ under changing conditions. We reasoned that the human digestive systems may be a rich resource for PET hydrolases, due to ubiquitous use of PET packaging for consumer food and drinks. Humans can mechanically digest plastics, which may generate microplastics known to affect the digestive tract and alter the gut microbiome.^[13]^ While precise health effects of microplastic consumption are currently unknown, gastrointestinal adaptations to deal with potentially cytotoxic microplastics may arise through changes and evolution of the gut microbiome.

Beyond plastic degradation, PET hydrolases represent untapped resources for biotechnologies surrounding PET. Hydrolase enzymes can be repurposed to detect and derivatize their substrates. A prominent example is HaloTag, a haloalkane dehalogenase engineered into a sitespecific protein labeling tag via disruption of its hydrolysis of the covalent alkyl-enzyme intermediate.^[14]^ Beyond HaloTag, mutagenesis of hydrolases to specifically affect the hydrolysis step of the covalent enzyme-substrate intermediate is difficult. In the case of serine hydrolases, the same set of residues—particularly the catalytic histidine and aspartate/glutamate of the Ser/His/Asp-Glu catalytic triad, and residues constituting the oxyanion hole—is thought to catalyze the formation of the acyl-enzyme intermediate and its subsequent hydrolysis, rendering enzyme engineering to abrogate the latter step while leaving the former unaffected non-trivial.

Herein, we implemented a bioinformatic workflow to discover PET hydrolases from metagenomic data. Such a workflow was used to identify putative PET hydrolases from marine and human metagenomes, ten of which we selected for further biochemical characterization. The most active PET hydrolase, called MG8, was discovered from metage-nomic data of saliva from a healthy participant of a 2016 US-based phage-oral/gut microbiome relationship study,^[15]^ which was deposited in the MGnify metagenome database^[16]^ (MGnify study ID: MGYS00003486; NCBI sequence read archive ID: PRJNA327423). MG8 is a highly active hydrolase against different substrates—particularly PET plastics— across different temperatures (37–65 °C) and salinity conditions (up to 5 M NaCl), when compared to benchmark hydrolases including *Ideonella sakaiensis* PETase (*Is*PETase),^[3]^ engineered *Is*PETase variants, and cutinases. While MG8 is a canonical α/β hydrolase, it has a unique catalytic loop that may contribute to its high activity. Instead of employing traditional site-directed mutagenesis approaches, we used genetic code to encode 2,3-diaminopropionic acid (DAP) in place of the catalytic serine of MG8 and used this non-canonical amino acid to covalently trap the acyl-enzyme intermediate of MG8. We thereby converted this PET-degrading enzyme into a covalent binder of PET. MG8(DAP) covalently captures instead of degrading PET, and it can be used to functionalize PET plastics under mild conditions with protein cargos.

## Results and Discussion

### Discovering PET Hydrolase Candidates from Marine and Human Metagenomics

**Figure 1:**
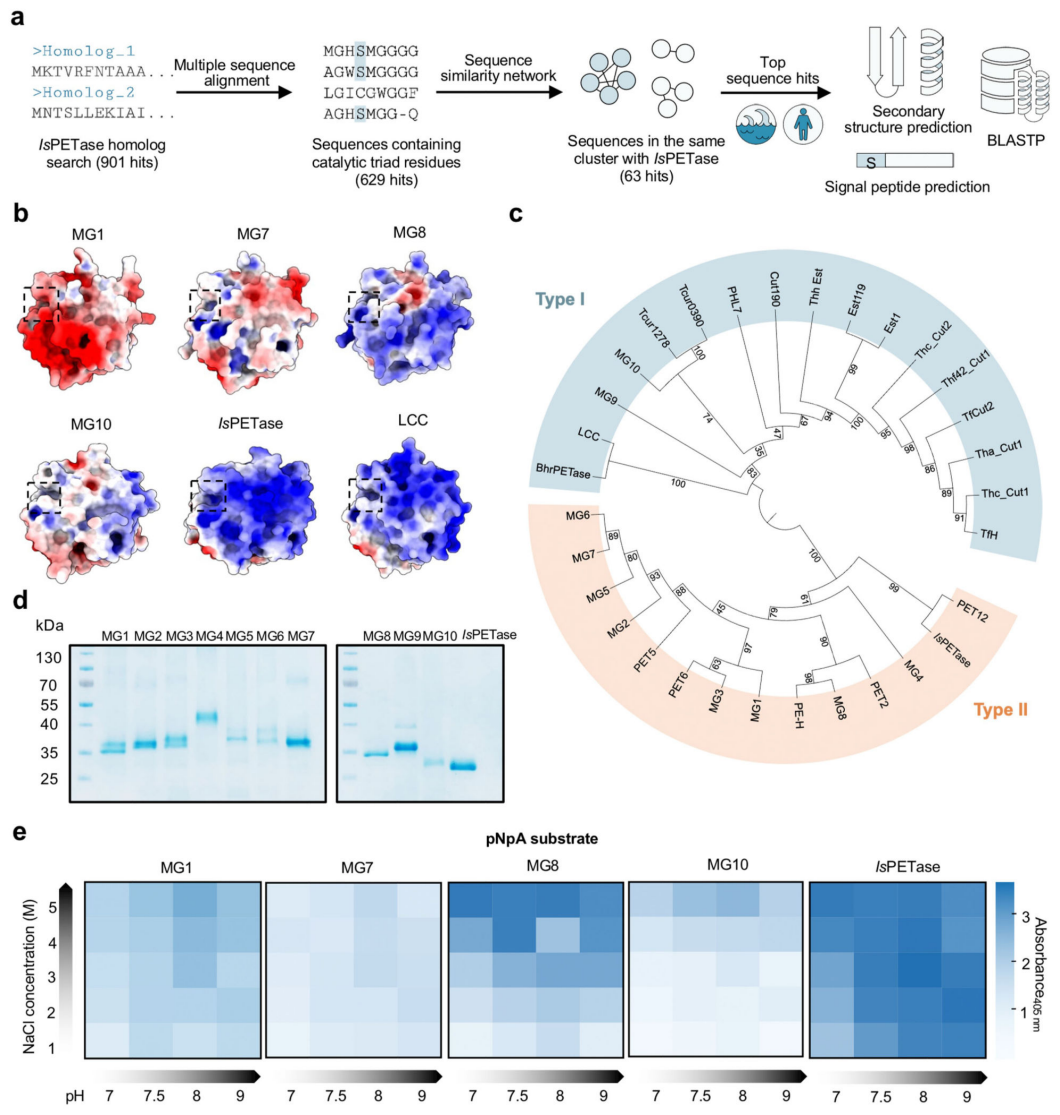
Bioinformatic discovery workflow and initial characterizations of putative PET hydrolases from marine and human metagenomics. a) Discovery workflow. PET hydrolase candidates were mined from the microbiome database MGnify through a non-redundant protein HMMER search using known PET-degrading enzymes, including *Ideonella sakeiensis* PET hydrolase (*Is*PETase), as a query. Candidate sequences were further filtered based on the presence of required catalytic triad residues of serine hydrolases and analyzed for their relationship with known PET hydrolases under a sequence similarity network. Prediction of secondary structures, signal peptide presence, and putative host organisms was further performed on ten selected candidates, named MG1-MG10. b) Electrostatic potential maps of modelled structures of MG1, MG7, MG8, and MG10 PET hydrolase candidates, along with similar maps of crystal structures of *Is*PETase (PDB:6EQE) and Leaf and Branch Compost Cutinase (LCC, PDB:4EB0), highlight the differences in global and local surface charges of the proteins. Red and blue colors represent negative (acidic residues) and positive (basic residues) potentials, respectively (scale of –5.0 to + 5.0 k_B_T/e_c_). The dashed contour outlines a putative active site of each structure. c) Sequence-based classification of PET hydrolase candidates. A maximum likelihood phylogenetic tree of PET hydrolase candidates and known PET hydrolases is shown. Bootstrap values at each node are from 100 replicates. Type I and Type II enzymes, previously classified by Joo et al.,^[20]^ contain distinct key residues summarized in Figure S6. The list of reference PET hydrolases was taken from a review by Carr et al.,^[21]^ with the addition of Est1,^[22]^ Est119,^[23]^ and PHL-7.^[8]^ d) SDS-PAGE of purified MG1-MG10. The major 1–2 bands in each lane represent the full-length hydrolase and a processed hydrolase with its signal peptide removed. e) Measuring relative esterase activity of putative PET hydrolases using *p*-nitrophenyl acetate (pNpA). Heat maps showing the activity of 300 nM MG1, MG7, MG8, and MG10 in hydrolyzing 5 mM pNpA under different pHs (7–9) and NaCl concentrations (1–5 M). The end-point mean absorbance values at 405 nm after 12 min reactions from three replicates are shown. For a full set of data for all ten enzymes and at a broader pH and salt concentration ranges, see Figure S8.

To search for putative PET hydrolases, we explored MGnify,^[16]^ a public database of microbiome data collected from diverse environments; as of January 2022, MGnify contained > 140 000 samples from human, and >45 000 samples from aquatic systems, of which ≈75% were from marine systems. We performed a non-redundant protein homolog (HMMER) search on the database using *Is*PETase as a query (Figure 1a). 901 amino acid sequences were retrieved; among these, 629 sequences were potential PET hydrolase candidates as they contained the required catalytic triad residues (S160, D206, and H237 in *Is*PETase). We generated a sequence similarity network with the Enzyme Similarity Tool (EFI-EST^[17]^) using these candidate sequences and other known PET hydrolases as input (Figure S1). Almost all known PET hydrolases with high activity—including *Is*PETase, LCC, *Thermobifida fusca* cutinase^[18]^ (TfH), and PE-H—were clustered together, along with additional 63 PET hydrolase candidate sequences.

Within this cluster, we were interested in PET hydrolase candidates from the marine (43 candidates) and human microbiomes (3 candidates). The high-saline environment of the ocean and human body fluids could yield PET hydrolase enzymes with high salt tolerance or salt-dependent catalytic activity while maintaining activity at low or near-ambient temperature similar to their native environments. As the *Is*PETase discovery supported the notion of its microbial evolution to utilize PET in the nearby environment, we empirically searched for candidates from marine microbiomes that may be located in/near ocean garbage patches, the compositions of which are mainly plastics (Figure S2). Lastly, as even the most homologous enzyme (MGYP000191526608, or MG3) to *Is*PETase shared only 68% sequence similarity compared to *Is*PETase, we picked the top entries in terms of sequence similarity to ensure the maximal likelihood of finding active enzymes.

Ultimately, seven putative PET hydrolases (MG1–MG7) with marine microbiome origins and three (MG8–MG10) with human microbiome origins were selected for further characterization (sequence-level information of the ten enzymes in Table S1; MGnify attributes in Table S2; sequence similarity to reference enzymes in Table S3). Our MG enzymes are distinct from previously characterized PET hydrolases from marine organisms or metagenomes (PE-H, PET2,^[11]^ PET5,^[11]^ PET6,^[11]^ and PET12^[11]^) with at most 74% sequence similarity between MG enzymes and known marine PET hydrolases (Table S3). Protein BLAST alignment searches indicated that the putative hosts of MG1–MG8 are from the phylum *Proteobacteria* (Gram-negative bacteria), while those of MG9 and MG10 belong to the phylum *Actinobacteria* (Gram-positive). Secondary-structure predictions with PSIPRED^[19]^ suggested canonical α/β-hydrolase folds for all candidates (Figure S3), almost all of which (except for MG4) also contain putative secretion signal peptides—suggestive of their potential natural roles in hydrolytic degradation of ester substrates in the extracellular milieu (Figure S4).

Despite similar putative structural folds, the surface charge—both global and local—of each enzyme candidate is predicted to be different. Such variation could greatly influence their substrate recognition and catalysis (Figure 1b and Figure S5). Structural homology modelling followed by predicted electrostatic potential mapping suggested that MG1–MG7 and MG9 enzymes contain overall acidic surface charges, consistent with their predicted isoelectric points (pI 4.25–5.22, Table S1) and halophilic origins. Yet, local charges around the putative active-site cleft of each enzyme candidate are different: MG1 and MG3 contain acidic active sites, while the rest of marine-origin candidates have more neutral active sites. MG8, an enzyme from a human saliva metagenome, is predicted to contain many surface patches of basic residues, resulting in an overall pI of 9.23 (similar to the values of *Is*PETase and LCC) but its active site is neutral. MG10, from a human skin microbiome, contains quite evenly distributed patches of acidic and basic residues, resulting in a near-neutral predicted pI of 6.41 (similar to TfH and PE-H) and a neutral active site.

From sequence and structural similarity calculations, we classified PET hydrolase candidates into 2 classes proposed by Joo et al.^[24]^ (Figure 1c; key residues listed in Figure S6). MG9 and MG10 putatively belong to class I PET hydrolases (which include TfH and LCC), which lack a stabilizing disulfide bond as well as extra residues following S238 in the catalytic histidine loop (S238 is a gatekeeping residue known to influence substrate recognition;^[25]^ *Is*PETase residue numbering). MG1–MG8 belong to class II, which includes *Is*PETase and PE-H.

### Characterizations of Putative PET Hydrolases MG1–MG10

We cloned, expressed, purified, and evaluated esterase activity of MG1–MG10. Using a conventional *E. coli* BL21-(DE3) expression host, we found that these enzymes were partitioned into insoluble inclusion bodies (Figure S7). Their poor solubility could arise from the preference of hypersaline conditions. For several enzyme candidates, their operons also contain genes encoding dedicated chaperones, which likely assist in folding of proteins expressed from these operons (Table S1). We attempted the following rescues to get sufficient protein into the soluble fraction without much success: titrating down expression levels; using *E. coli* Origami 2(DE3) strain with enhanced protein folding capacity; adding a solubilizing small ubiquitin-like modifier (SUMO) tag; and removing the signal peptide. Ultimately, we extracted and affinity-purified the proteins under denaturing conditions of 6 M urea, before refolding them during dialysis. We obtained all MG1–MG10 enzymes in good purity and yields (7–15 mg protein per 1 L culture); for some enzymes, two major bands—corresponding to the fulllength protein and a processed protein with its signal peptide removed—were observed on SDS-PAGE (Figure 1d).

We assayed purified MG1–MG10 for their generic esterase activity. Using *p*-nitrophenylacetate (pNpA) as a hydrolysis substrate, we also assessed whether the enzymes function optimally under certain pH and salinity conditions. We found that all enzymes exhibited esterase activity against pNpA, and all worked better at slightly basic pH, consistent with enhanced nucleophilicity of the catalytic serine at higher pH (Figure S8). Among the variants, MG1, MG7, MG8, and MG10 were the most active, and interestingly, all four enzymes showed clear salt (NaCl)-dependent activity: higher NaCl concentrations of up to 5 M led to consistent increase in their pNpA hydrolysis activities (Figure 1e). The salt-dependent activity of the MG enzymes is consistent with their hypersaline environment origins.

We next tested enzymatic hydrolysis of a small-molecule PET surrogate substrate, bis(2-hydroxyethyl) terephthalate (BHET). Similar to results with pNpA, MG1, MG7, and MG8 were the most active in hydrolyzing BHET, and showed increase in activity at higher NaCl concentrations (Figure S9, S10). However, their BHET hydrolysis activities at 37 °C were at best 27% of *Is*PETase activity (Figure S9). While BHET hydrolysis is likely a prerequisite activity of a hydrolase capable of degrading PET plastics, the hydrolysis efficiency of PET hydrolases against small-molecule BHET is a poor predictor of how well they hydrolyze rigid PET plastics.^[24]^ Therefore, we proceeded to test all enzymes for their efficiency in hydrolyzing PET powder.

### Efficient PET Plastic Degradation by MG8, a Novel Hydrolase from the Human Saliva Metagenome

**Figure 2:**
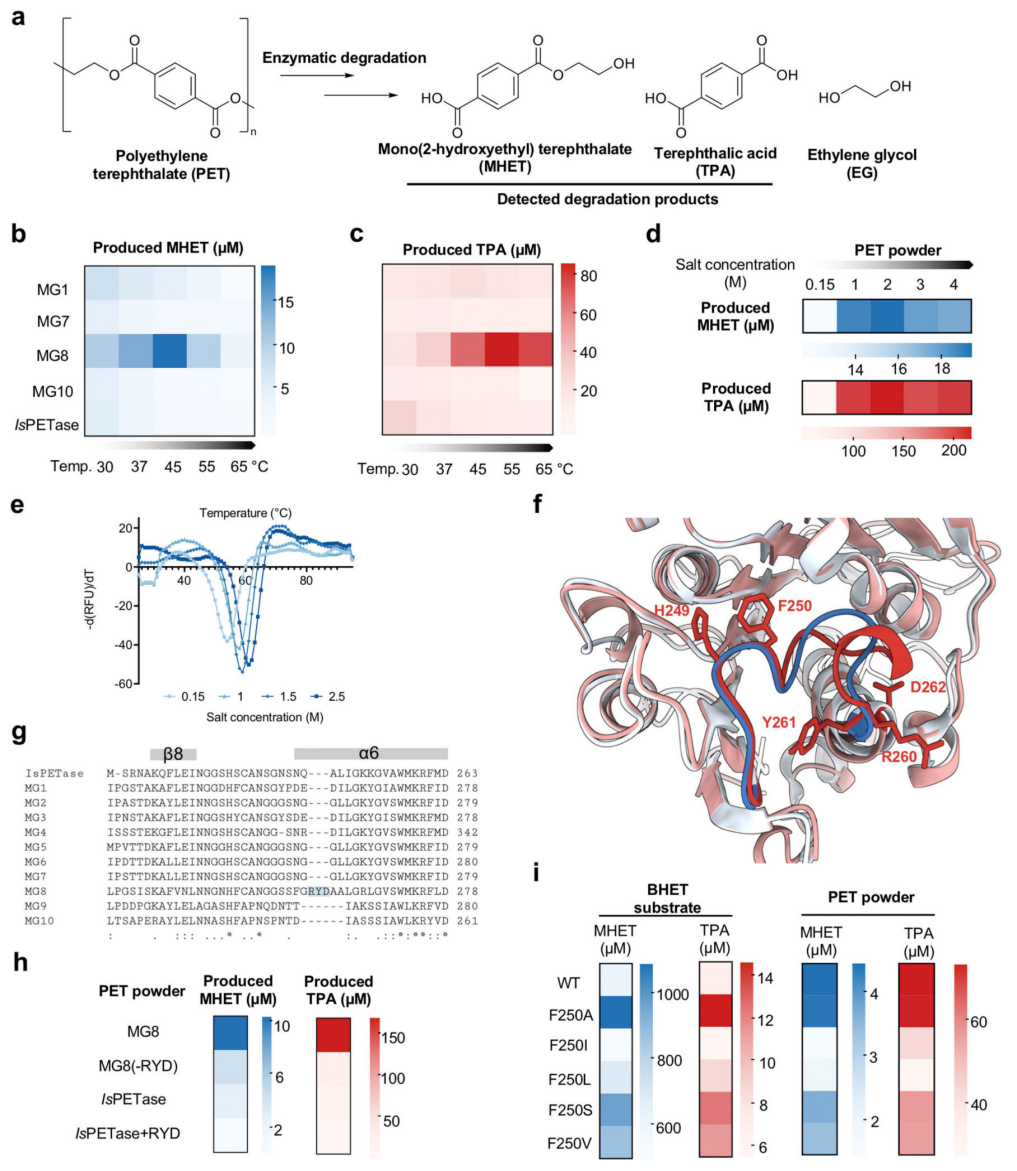
Efficient degradation of PET plastic by MG8 PET hydrolase from human saliva metagenome. a) Mono(2-hydroxyethyl) terephthalate (MHET) and terephthalic acid (TPA) generated from the degradation of PET powder were monitored by HPLC. b) Relative activity of 500 nM enzyme (MG1, MG7, MG8, or MG10) in hydrolyzing 20 mg PET powder to generate MHET (left) and TPA (right). Hydrolysis was allowed to proceed in buffer containing 4 M NaCl for 48 h at different temperatures (30, 37, 45, 55, or 65 °C), before reactions were quenched with 5 mM phenylmethylsulfonyl fluoride (PMSF) and analyzed by HPLC. *Is*PETase activity to generate MHET under the same condition acts as a reference point. c) TPA generated from the same reactions as in b). Each entry in the heat map in b) and c) represents mean values from duplicate experiments; for bar graphs, see Figure S11. d) High activity of MG8 in hydrolyzing PET powder to produce MHET (top) and TPA (bottom) at 1–4 M NaCl concentrations. Heat maps show mean amount of products generated in triplicate experiments. For bar graphs, see Figure S12. e) Higher thermal stability of MG8 at higher NaCl concentrations. f) Superimposition of a modelled MG8 structure and the crystal structure of *Is*PETase (PDB: 6EQE) highlights the extended catalytic histidine loop of MG8 (shown in red, compared to the loop from *Is*PETase in blue). The catalytic H249, adjacent F250, and the putative three-residue addition (RYD 260–262) of MG8 are shown. g) Multiple amino acid sequence alignment highlights the unique length of the catalytic histidine loop of MG8. h) Effects of deletion of the extended RYD loop from MG8 (MG8(-RYD)) and grafting of the RYD loop onto *Is*PETase (*Is*PETase+ RYD) on enzyme activity. Generated MHET and TPA from PET powder degradation assay performed in 4 M NaCl and at 55 °C for 48 h are shown. Heat maps show mean amount of products generated in triplicate experiments. For bar graphs with individual data points, see Figure S21. i) Generation of MHET and TPA from hydrolysis of BHET (left) and PET powder (right) with MG8-F250 mutants. Heat maps show the mean amount of products generated in triplicate experiments. For bar graphs with individual data points, see Figure S22.

We used the insights obtained from previous activity assays on the four MG enzymes—particularly their high-salt, basic-pH preference—in designing optimal degradation conditions for crystalline PET powder (≈ 30% crystallinity as measured by differential scanning calorimetry). We found that at 37 °C, MG8 degraded PET powder to produce detectable mono (2-hydroxyethyl) terephthalate (MHET) twenty-six times and terephthalic acid (TPA) three times more efficiently than *Is*PETase (Figures 2a–c and S11a for data of the most active enzymes; Figure S11b–c for data of the rest of enzymes, and their activity in units). We further optimized reaction temperatures and saw further 5–7-fold enhancement in TPA production, and 2-fold enhancement in MHET production, when MG8 was deployed at 45–65 °C (Figure 2b, c). At the optimal temperature of 55 °C of MG8, the enzyme produced ≈ 83-fold more TPA than *Is*PETase at its optimal condition (Figure 2c). For MG8, product profiles shifted toward more TPA—which can be further bioconverted to value-added chemicals such as vanillin^[1]^ and gallic acid^[26]^—than MHET at higher reaction temperatures (Figure 2b, c) and more basic pH (Figure S13). When both MG8 and *Is*PETase were deployed at 55°C–where degradation can function closer to the glass transition temperature of PET (≈70 °C^[27]^), facilitating degradation of crystalline PET —MG8 outperformed *Is*PETase in degrading PET powder by ≈ 68-fold, as measured from combined hydrolysis products (Figure S13).

**Figure 3:**
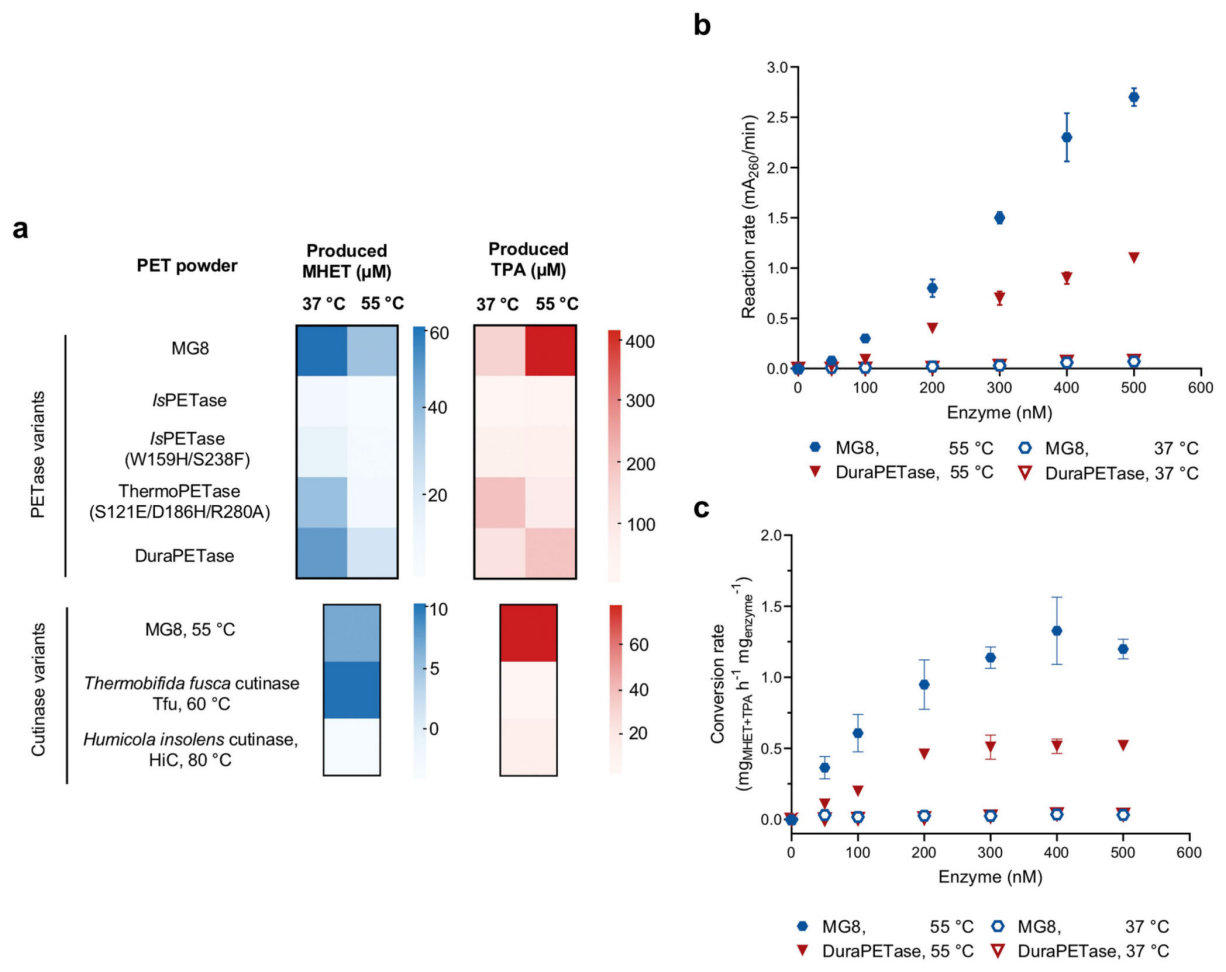
Comparing PET degradation efficiencies and kinetics of MG8 to engineered *Is*PETase variants, *Thermobifida fusca cutinase* (Tfu), and *Humicola insolens* cutinase (HiC). a) Relative activity of 500 nM enzyme in hydrolyzing 10 mg PET powder to generate MHET (left) and TPA (right). Hydrolysis was allowed to proceed for 48 h at 37 °C or 55 °C (for MG8 and PETase variants), 60 °C for Tfu, and 80 °C for HiC, before quenching and product analysis by HPLC. Each heat map entry is mean product concentration from triplicate experiments. For bar graphs with error bars, see Figure S14. b) Hydrolysis rates of PET powder as a function of enzyme concentration. PET hydrolysis reactions were performed with 15 mg PET powder and 0–500 nM MG8 or DuraPETase at 37 °C or 55 °C. Reaction rates were calculated in terms of absorbance at 260 nm^[31]^ (A_260_, which reflects generated TPA and MHET, the primary soluble hydrolysis products) per minute, at a given enzyme concentration (*n*=3 per concentration). Error bars, ± SD. c) Enzymatic conversion rates to generate TPA and MHET hydrolysis products from PET powder (same underlying data as b). Generated TPA and MHET amount was calculated from calibration curves between measured absorption values and concentrations of TPA and MHET standards, with the assumption that generated TPA:MHET molar ratio (measured at the time-coursed endpoint via HPLC) was constant throughout the reaction.

We further compared MG8 activity against other engineered PETase variants and natural cutinases. MG8 is more active in degrading PET powder than *Is*PETase^W159H/S238F^, ThermoPETase (*Is*PETase^S121E/D186H/R280A^)^[28]^ and DuraPETase^[29]^ by ≈ 43-fold, ≈ 21-fold and ≈ 5-fold respectively (Figures 3a and S14). Comparison of MG8 to two other widely used PET hydrolases—*Thermobifida fusca* cutinase^[18]^ (Tfu) and *Humicola insolens* cutinase^[30]^ (HiC), each used at their preferred reaction temperatures (Tfu, 60 °C; HiC, 80°C)—showed that MG8 (used at 55°C) is more active than both enzymes in degrading PET plastics by 17–23-fold (Figures 3a and S15). MG8 has a maximal catalytic rate when ≈ 500 nM enzyme was used (Figure S16) and showed at least 3-fold higher rates (and at least 2.5-fold higher conversion rates upon normalization with enzyme amount) compared to DuraPETase, across 50–500 nM enzyme concentration ranges at two reaction temperatures (37 and 55 °C; Figures 3b, c and S17, S18).

Despite the high activity of MG8 in releasing PET monomers from crystalline PET powder, we did not observe significant mass reduction of the powder after enzymatic treatment. Crystallinity of PET powder after MG8-mediated degradation remained largely unchanged (≈30%, Figure S19), suggesting that the enzyme digested monomers from minor amorphous portions of the polymer. This difficulty in depolymerizing highly crystalline PET is intrinsic to all PET hydrolases discovered so far,^[32]^ and remains an outstanding challenge to overcome for the field.

To understand the basis of MG8’s high PET degradation activity, we first characterized its thermal stability: MG8 has a melting temperature (*T*_m_) of 54 °C, compared to 42°C of *Is*PETase (Figure S20). As we saw better MG8 activity at higher temperatures and NaCl concentrations (Figure 2d), we measured its *T*_m_ at higher NaCl concentrations used in hydrolysis reactions: *T*_m_ of MG8 increased to 62 °C in the presence of 2.5 M NaCl (Figure 2e). While the overall pI of MG8 is predicted to be basic (9.23), clusters of acidic amino acids at the protein surface, along with hydrated salt ions, may facilitate protein hydration and maintenance of its fold and activity at higher salt concentrations.^[33]^

Another unusual feature of MG8 is the presence of an extended sequence in the catalytic histidine (H249 for MG8) loop between β-strand 8 and α-helix 6 (Figure 2f). Multiple amino acid sequence alignment highlighted the presence of three additional residues—putatively RYD, residues 260–262—in MG8 that is absent from all other MG enzymes as well as other known PET hydrolases (Figure 2g). Deletion of RYD residues from MG8 reduced its PET powder degradation activity by 5–30-fold while addition of the RYD residues to the catalytic histidine loop of *Is*PETase did not improve *Is*PETase activity, suggesting that the importance of this extended sequence is specific to the context of MG8 (Figure 2h). It is postulated, based on molecular dynamics simulations, that PET hydrolases with higher flexibility in the catalytic loop regions allow them to accommodate binding to heterogeneous yet rigid structures of PET plastics.^[34]^ The extended catalytic histidine loop of MG8 may contribute to the enzyme’s high activity in this manner.

A related feature of the catalytic histidine loop of MG8 is its F250 residue, which flanks the catalytic H249. Most PET hydrolases have small amino acids (particularly serine) at this position, presumably to facilitate flexible movement of the histidine residue during the catalytic cycle.^[10]^ In the case of MG8, we found that mutating its F250 to small- and medium-sized amino acids (S, A, I, L, and V) had little positive effect on PET hydrolysis activity. The only positive trend observed was MG8 (F250A), which could hydrolyze BHET better than wild-type MG8 (≈3-fold higher combined product output), but its PET hydrolysis efficiency remained similar to the wild-type enzyme (Figure 2i). Perhaps F250 can assist MG8 in binding to hydrophobic PET plastic substrates, and the extended length of MG8’s catalytic histidine loop compensates for the more restricted loop mobility due to the presence of a bulky residue adjacent to H249.

While we initially purified MG8 under denaturing conditions which necessitated refolding, we later successfully purified MG8 from small soluble fractions using large-scale cultures. MG8 purified under non-denaturing conditions has better hydrolysis activity against PET powder than refolded MG8 (Figure S23), but the latter can be produced at higher yields. The ease at which MG8 can be denatured and refolded while retaining decent activity should facilitate its large-scale production.

### Converting MG8 into a Covalent Binder for PET via Genetic Encoding of 2,3-diaminopropionic Acid

**Figure 4:**
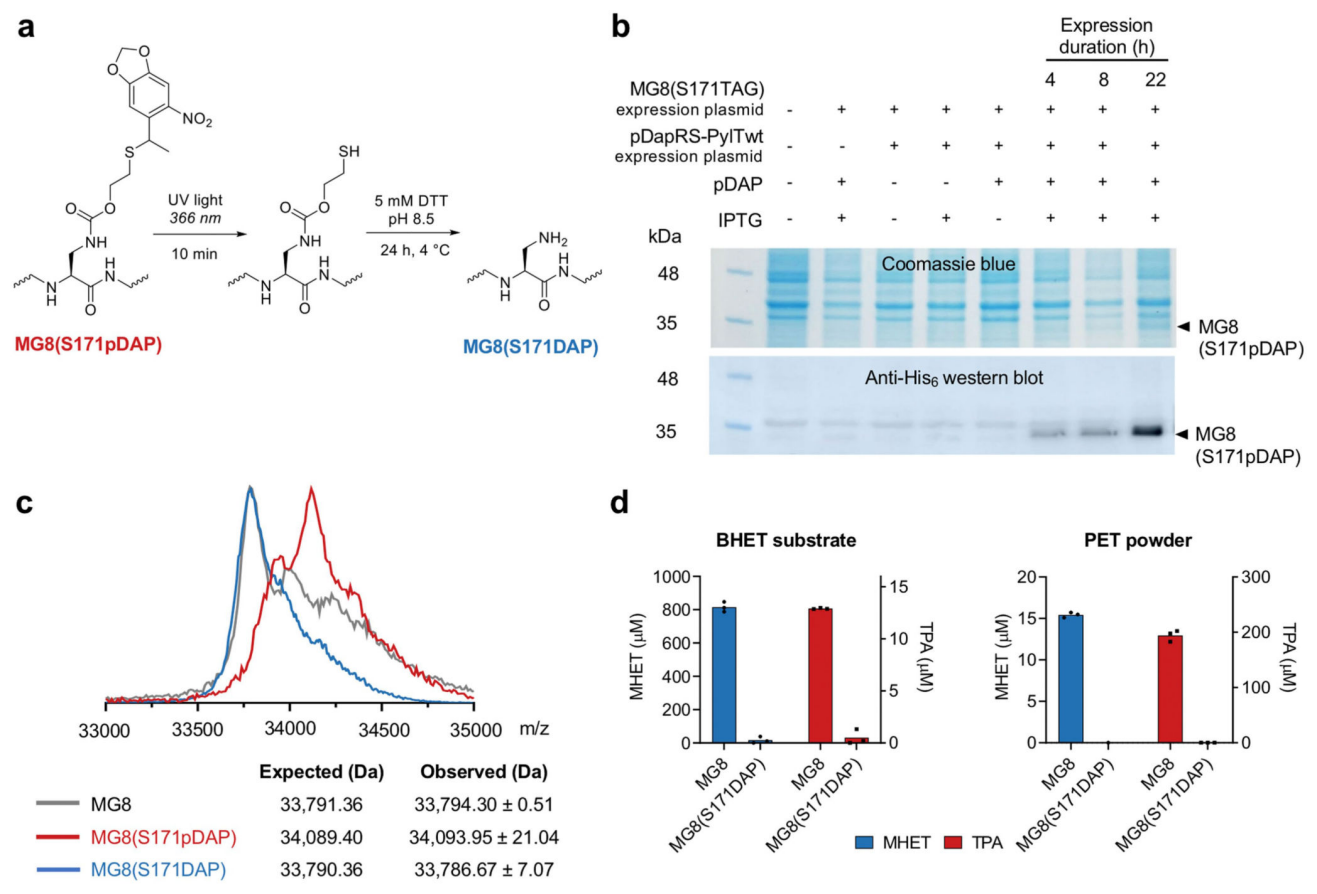
Replacement of the catalytic serine residue of MG8 with 2,3-diaminopropionic acid (DAP) abrogates its PET degradation activity. a) Genetic encoding of photocaged DAP (pDAP) to replace S171 of MG8. UV illumination initiates uncaging of pDAP, which further decomposes to produce DAP. b) SDS-PAGE of MG8 with incorporated pDAP at position 171. Proteins were visualized via Coomassie Blue (top) or anti-His_6_ western blotting (bottom). The gel shown is representative of two biological replicates which gave similar results. c) MALDI-TOF mass spectrometric analysis of purified MG8, MG8(S171pDAP), and MG8(S171pDAP) after photo-deprotection to produce MG8(S171DAP). d) MG8(S171DAP) is minimally active in hydrolyzing BHET (left) and PET powder (right). Generated MHET and TPA from MG8- or MG8(S171DAP)-catalyzed degradation of 3 mM BHET (55 °C for 30 min) or 20 mg PET powder (55 °C for 48 h) are shown. Each bar graph represents mean values from triplicate experiments.

Beyond PET plastic degradation, we explored whether MG8 can be used as a tool for surface functionalization of PET, a popular choice of substrate for wearable sensors^[35]^ due to its flexibility and deformability. A transient acyl-enzyme intermediate is formed between MG8 and PET during MG8-mediated PET hydrolysis. Stable trapping of such an intermediate is possible through site-specific replacement of the catalytic serine of MG8 with its isosteric amino analog, 2,3-diaminopropionic acid (DAP)^[36] [37]^ via genetic code expansion (Figure 4a, 5a). Instead of a labile ester bond, DAP-incorporated MG8 traps PET via an amide linkage, rendering the resulting enzyme-plastic complex highly stable. We thus envisioned creating a covalent binder for PET, using DAP-incorporated MG8 as a medium; desired protein cargoes can be adsorbed onto the PET surface either via direct fusion of the cargo to MG8(DAP), or more modularly, via a protein-protein conjugation tool such as the split-GFP^[38]^ or SpyTag system.^[39]^ Immobilizing proteins on PET via MG8(DAP) would not require harsh (and often sample-degrading^[40]^) chemical treatments of the inert plastic surface^[41]^ to generate sites for bioconjugation. Moreover, as MG8(DAP) needs to orient itself to react with PET (via the enzyme’s active site), the orientation of MG8(DAP)-linked protein cargoes adsorbed onto the plastic surface will be non-random, and may be precisely tuned for maximal activity and stability of the cargoes.

We first produced MG8 in which its catalytic serine, S171, was replaced by photocaged DAP (pDAP, Figure 4b), an efficient substrate for the engineered *Methanosarcina barkeri* pyrrolysyl-tRNA synthetase (pDAPRS)/Pyl tRNA_CUA_ pair.^[36]^ During protein purification, we photodeprotected MG8(S171pDAP) via UV illumination; subsequent thiocarbonate release by intramolecular cyclization under basic conditions produced MG8(S171DAP), which we confirmed via MALDI-TOF mass spectrometry (Figure 4c). We verified that MG8(S171DAP) is highly deficient in catalyzing hydrolysis of BHET and PET powder, consistent with MG8(S171DAP) being able to catalyze only the first round of the acyl-enzyme intermediate formation, then being trapped. Little degradation activity (≈3–6% compared to wild-type MG8) was observed with BHET, and no degradation was observed with PET powder (Figure 4d).

**Figure 5:**
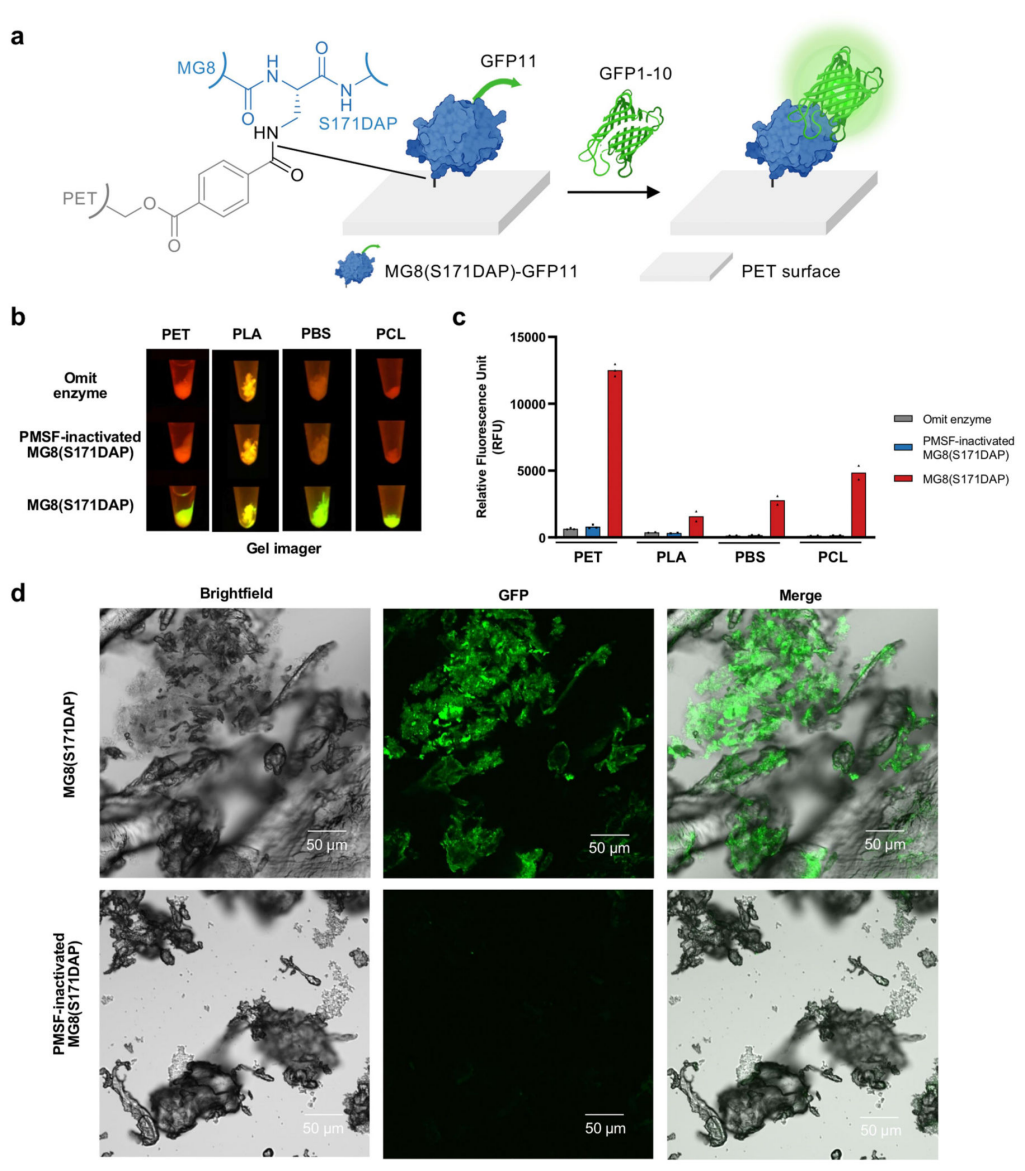
DAP incorporation in MG8 enables its stable attachment to PET plastic. a) Visualizing immobilized MG8(S171DAP) on PET with split GFP complementation. The smaller, GFP11 b-strand fragment of the split GFP system is genetically fused to the C-terminus of MG8(DAP). After immobilization of MG8(DAP)-GFP11 onto PET powder via DAP-mediated amide linkage, the protein is visualized via addition of the GFP1-10 fragment and subsequent reconstitution of fluorescent GFP. b) PET, polybutylene succinate (PBS), polycaprolactone (PCL), and polylactic acid (PLA) can be fluorescently labeled with split GFP via MG8(S171DAP). 10 mg plastic powder was incubated with 7.59 μM of purified MG8(S171DAP)-GFP11 at 50 °C for 1 h. After washing to remove excess MG8(S171DAP)-GFP11, the powder was further incubated with 49 μM GFP1-10 at 37 °C for 24 h. Plastic powder was washed extensively before visualization directly in an Eppendorf tube. Results from triplicate experiments for PET and duplicate experiments for PBS, PCL, and PLA are shown (for replicate data, see Figure S24). c) Quantification of GFP fluorescence signals from b). d) Confocal imaging of GFP-labeled PET powder via MG8(DAP)-GFP11 and split GFP reconstitution. Scale bars, 50 μm. For b–d), negative controls are shown with MG8(S171DAP)-GFP11 inactivated with 5 mM PMSF prior to addition to PET.

To enable visualization of MG8(S171DAP) immobilized on PET, we genetically appended the GFP11 β-strand fragment of the split GFP system^[38a]^ to the C-terminus of MG8(S171DAP) (Figure 5a). Incubation of MG8-(S171DAP)-GFP11 with PET powder for 1 hr followed by addition of the complementary GFP1-10 fragment produced strong fluorescence signal (20-fold higher signal than negative controls)—in a MG8 activity-dependent manner— from GFP reconstituted on PET (Figure 5b, c). Confocal images revealed largely homogeneous distribution of GFP signal on PET particles (Figure 5d), indicating successful deposition of the protein cargo at the plastic surface. The platform—already modular due to the two-component split-GFP system, and visualizable due to the fluorogenic indicator of successful cargo delivery—should be extensible to the functionalization of PET surface with diverse cargos, particularly with single-component protein-based biosensors.

Due to MG8’s likely ability to hydrolyze aliphatic polyesters (similar to other PET hydrolases^[42]^), we also demonstrated that MG8(DAP) could be used to biofunctionalize aliphatic polyesters such as polybutylene succinate (PBS), polycaprolactone (PCL), and polylactic acid (PLA), in addition to PET (Figure 5b, c).

One bottleneck to the use of the DAP system is the lengthy (7 steps; over 70 h of reaction times) synthesis of pDAP.^[36]^ We optimized the previously reported synthetic route of pDAP to be more time-efficient (22 h total reaction times), while maintaining comparable yields and using reagents more readily available in resource-limited settings (see Methods). The improved synthetic route of pDAP, which we accomplished in gram scale, should permit more accessible use of the DAP system for diverse applications.

## Conclusion

In summary, we systematically explored metagenome data and identified a new PET hydrolase, MG8, from the human saliva metagenome. While there is a plethora of existing efficient PET hydrolases, we believe MG8 is a key addition to this collection. It is the first PET hydrolase identified from the human metagenome—a vast but largely underexplored resource for industrially relevant enzymes. Without any enzyme engineering, MG8 already exhibits robust activity across a range of temperature and salinity conditions, and outperforms several naturally occurring and engineered PET hydrolases in terms of PET degradation efficiency. While MG8 is likely not as active as state-of-the-art PET hydrolases such as FAST-PETase^[31]^ and LCC^WCCG or ICCG^,^[2]^ rapid advances in enzyme engineering previously applied to other PET hydrolases—particularly machine learning-based approaches recently used to create FAST-PETase^[31]^ as well as high-throughput screening/selection platforms^[43, 44]^—can likewise be applied to further improve the properties of MG8. Further investigations of MG8’s properties through interfacial kinetic,^[45, 46]^ computational, and structural studies with relevant enzyme substrates/ligands^[44]^—ideally alongside other PET hydrolases—can shed light on features of these enzymes which contribute to their differences in activity, and facilitate further engineering.

It is currently challenging to explore the functional space of new metagenomic enzymes. Enzymes from taxonomically distant origins may not express well in conventional heterologous hosts like *E. coli*; high-throughput approaches to evaluate these enzyme candidates are best used in conjunction with dedicated biochemical optimizations to identify best functioning conditions for promising candidates. Since metagenomics is vast, there is always a bias in selecting the sequence space to experimentally validate function. Here we were conservative in enzyme selection; by selecting putative PET hydrolases with high sequence similarity to known PET hydrolases, we ensure higher likelihood of identifying active enzymes, but miss out on candidates with lower sequence identity/similarity to reference enzymes, which may have distinct properties complementing the existing PET-hydrolase arsenal.

Genetic code expansion has previously been used in conjunction with click chemistry for protein immobilization onto magnetic microparticles,^[47]^ quantum dots,^[48]^ gold-coated surfaces,^[47]^ and Sepharose resin.^[49]^ Controlled protein orientation provided by genetic code expansion and other site-specific protein labeling tools has been shown to improve protein activities on functionalized surfaces.^[47, 48]^ By applying the genetic code expansion technology to MG8, we successfully converted a PET hydrolase into a PET binder; MG8(DAP) can be used to covalently link protein cargos to PET plastics without the need of harsh surface treatment steps, and may be a useful tool for the creation of wearable sensors and plastic products that detect disease biomarkers^[35,50]^ and therapeutic levels.^[51]^

Beyond biotechnological applications, DAP can be used to trap acyl-enzyme intermediates of PET hydrolases for structural studies, facilitating better understanding of polymeric substrate recognition and structure-guided enzyme engineering.

## Supplementary Material

Supplementary Information

## Data Availability

The data that support the findings of this study are available from the corresponding author upon reasonable request.
