# Supplementary material for: Discovery and Genetic Code Expansion of a Polyethylene Terephthalate (PET) Hydrolase from the Human Saliva Metagenome for the Degradation and Bio-Functionalization of PET
Source: Angew Chem Int Ed Engl. Author manuscript; Available in PMC 2022 Nov 14. (PMC7613822; doi:10.1002/anie.202203061)
Supplement: Supplementary Information [file EMS156766-supplement-Supplementary_Information.pdf]

Angewandte Chemie
Eine Zeitschrift der Gesellschaft Deutscher Chemiker

Supporting Information

Discovery and Genetic Code Expansion of a Polyethylene Terephthalate (PET) Hydrolase from the Human Saliva Metagenome for the Degradation and Bio-Functionalization of PET

B. Eiamthong, P. Meesawat, T. Wongsatit, J. Jitdee, R. Sangsri, M. Patchsung, K. Aphicho, S. Suraritdechachai, N. Huguenin-Dezot, S. Tang, W. Suginta, B. Paosawatyanyong, M. M. Babu, J. W. Chin, D. Pakotiprapha, W. Bhanthumnavin, C. Uttamapinant**

Table of Contents

1. Experimental Procedures
1.1 Chemicals and synthetic methods 3
1.1.1 Chemicals for the synthesis of pDAP 3
1.1.2 Chemicals and reagents for biochemical assays 3
1.1.3 NMR methods 3
1.1.4 Differential scanning calorimetry (DSC) for Tm, Tg, and crystallinity characterization of PET 3
1.1.5 Synthesis of photocaged DAP 3
1.2 Bioinformatic workflow to identify putative PET-degrading enzymes 5
1.3 Homology modelling and electrostatic potential calculations 5
1.4 Molecular cloning 5
1.5 Protein expression and purification 5
1.6 Assessing esterase activity of putative PET-degrading enzymes using p-nitrophenyl acetate (pNpA) under different pH and salt concentrations 6
1.7 Assessing PET hydrolase activity using a PET surrogate substrate, bis(2-hydroxyethyl) terephthalate (BHET) 6
1.8 Assessing PET hydrolase activity using PET powder 7
1.9 Assessment of thermal stability of PET-degrading enzymes 7
1.10 MALDI-TOF analyses of intact protein masses 7
1.11 GFP functionalization on PET plastic 7

2. Supplementary Information
2.1 Figure S1-24 8
2.2 Table S1-5 32
2.3 NMR Spectra 39

3 Supplementary References 46

Experimental Procedures

Chemicals and synthetic methods

Chemicals for the synthesis of pDAP

All reactions were performed in oven-dried glassware. All reagent-grade chemicals for the synthesis in this work were purchased from Acros, Merck, Sigma- Aldrich, and TCI and were used without further purification. Laboratory grade organic solvents from RCI Labscan were used for column chromatography and thin-layer chromatography. Unless otherwise specified, analytical grade organic solvents from Burdick & Jackson and RCI Labscan were used for reaction set ups. The progress of the reactions was monitored by thin-layer chromatography (TLC) performed on Merck D.C. silica gel 60 F_{254} 0.2 mm precoated aluminium sheets and visualized using UV light (254 nm). Column chromatography was performed on Merck 70-230 mesh ASTM silica gel. Solvents for NMR experiments were purchased from Cambridge Isotope Laboratories or Euriso-top.

Chemicals and reagents for biochemical assays

p-Nitrophenyl acetate (pNpA), and bis(2-hydroxyethyl) terephthalate (BHET) were purchased from Sigma-Aldrich, and terephthalic acid (TPA) from TCI; they were used without further purification. PET powder (~0.3µm-diameter powder) was a gift from PTT Innovation Institute; its characteristics, including crystallinity, were assessed with differential scanning calorimetry (DSC). The Solubility & Stability Screen 2 kit was purchased from Hampton Research. SYPRO^{TM} Orange protein gel stain was purchased from Thermo Fisher Scientific. Polybutylene succinate (PBS) and polylactic acid (PLA) were gifts from Assoc. Prof. Khamphee Phomphrai (VISTEC), and were obtained from PTT Global Chemical and NatureWorks, respectively. Polycaprolactone (PCL) was kindly provided by Assoc. Prof. Daniel Crespy (VISTEC), and was purchased from Wako.

NMR methods

NMR spectra were obtained from Jeol NMR spectrometer and were recorded at 500 MHz for ^{1}H NMR experiments and 126 MHz for ^{13}C NMR experiments, using deuterated dimethylsulfoxide (DMSO-d_6), deuterated chloroform ($CDCl_3$), and deuterated methanol-d_4 as NMR solvents. The chemical shifts are reported in parts per million (ppm) relative to tetramethylsilane signal (δ_H = 0.00 ppm) or residual protonated signal of deuterated solvent as a reference.

Differential scanning calorimetry (DSC) for T_m, T_g, and crystallinity characterization of PET

The melting temperature (T_m), glass transition temperature (T_g), and percent crystallinity of PET powder were characterized by a differential scanning calorimeter (PerkinElmer, DSC 8500 double furnace HyperDSC). PET powder (4-10 mg) was placed in standard alum pans with cover. The temperature of the samples was held at 0 °C for 1 min, then increased from 0 °C to 300 °C at the rate of 10 °C/min, and held at 300 °C for 1 min. The system temperature was subsequently reduced from 300°C back to 0 °C at the rate of -100 °C/min and held at 0 °C for 1 min. The heating and cooling cycle was repeated once more. The percent crystallinity was calculated using data obtained from the first heating, using the following equation:

$$\%Crystallinity = \frac{\Delta Hm - \Delta Hc}{\Delta Hm^{\circ}}$$

where ΔH_m is the enthalpy of melting (J/g), ΔH_c is the enthalpy of cold crystallization (J/g), and ΔH_m° is the reference value of enthalpy of melting for 100% crystalline PET (140.1 J/g [[4]]). ΔH_m and ΔH_c values were obtained by integrating peak areas from 210 °C to 264 °C, and from 100 °C to 150 °C, respectively. The glass transition temperature was obtained by integrating peak areas from 60 °C to 100 °C from second heating. The glass transition temperature of PET powder was 79.74±0.02 °C.

Synthesis of photocaged DAP

The initial batch (~100 mg) of photocaged DAP (pDAP, **7**) was generously provided by Prof Jason Chin, Dr Mahesh Mohan, Dr Nicholas Huguenin-Dezot, and Dr Shan Tang (MRC Laboratory of Molecular Biology, Cambridge, UK). Gram-scale synthesis of pDAP, with improvements to the overall shorter reaction times, was performed, as follows:

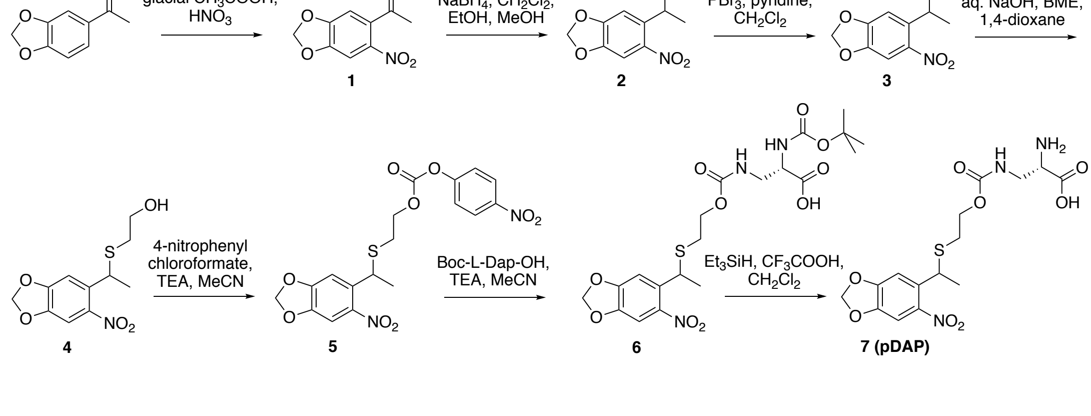

4',5'-Methylenedioxy-2'-nitroacetophenone **1.** We followed the protocol described in Huguenin-Dezot *et al.*[5], with the following modifications: 1) the 40 °C heating duration of the reaction was reduced from 2.5 h to 30 min; 2) after we obtained the precipitate, we proceeded directly to column chromatography without performing recrystallization. **1** was obtained as yellow solid (1.80 g from 4 g of 3',4'-(methylenedioxy)acetophenone starting material, 35%). ^{1}H NMR (500 MHz, $CDCl_3$) δ (ppm): 7.54 (s, 1H), 6.74 (s, 1H), 6.16 (s, 2H), 2.48 (s, 3H). ^{13}C NMR (126 MHz, $CDCl_3$) δ (ppm): 199.3 (C), 152.8 (C), 149.0 (C), 140.2 (C), 135.3 (C), 106.3 (CH), 105.0 (CH), 103.7 (CH_2), 30.4 (CH_3).

1-(4',5'-Methylenedioxy-2'-nitrophenyl)ethanol **2**. We followed the protocol described in Huguenin-Dezot *et al.*[5], with a minor scale-dependent modification: when small amount of the starting material was used (we used 1.25 g of **1**), the reaction was completed as determined by TLC 30 min after addition of $NaBH_4$ portions. **2** was obtained as yellow solid (1.26 g, 99%). ^{1}H NMR (500 MHz, $CDCl_3$) δ (ppm): 7.45 (s, 1H), 7.26 (s, 1H), 6.12-6.08 (m, 2H), 5.45 (qd, J = 6.2, 3 Hz, 1H), 2.26 (d, J = 3.5 Hz, 1H), 1.53 (d, J = 6.3 Hz, 3H). ^{13}C NMR (126 MHz, $CDCl_3$) δ (ppm): 152.5 (C), 147.0 (C), 141.6 (C), 139.1 (C), 106.4 (CH), 105.2 (CH), 103.0 (CH_2), 65.8 (CH), 24.3 (CH_3).

1-Bromo-(4',5'-methylenedioxy-2'-nitrophenyl)ethane **3**. We followed the protocol described in Huguenin-Dezot *et al.*[5], with a minor scale-dependent modification: when small amount of the starting material was used (we used 1.97 g of **2**), the reaction was allowed to proceed at r.t. after all reagent addition for 30 min, instead of 1.5 h. **3** was obtained as yellow solid (1.98 g, 77%). ^{1}H NMR (500 MHz, $CDCl_3$) δ (ppm): 7.33 (s, 1H), 7.25 (s, 1H), 6.11 (s, 2H), 5.88 (q, J = 6.8 Hz, 1H), 2.02 (d, J = 6.8 Hz, 3H). ^{13}C NMR (126 MHz, $CDCl_3$) δ (ppm): 152.1 (C), 147.7 (C), 141.6 (C), 134.8 (C), 108.8 (CH), 105.1 (CH), 103.3 (CH_2), 42.8 (CH), 27.6 (CH_3).

2-{[1-(6-Nitrobenzo[d][1,3]dioxol-5-yl)ethyl]thio}ethan-1-ol **4**. We tested THF (freshly distilled) and 1,4-dioxane (previously used by Huguenin-Dezot *et al*[5] as reaction solvent, and confirmed that the latter is a better solvent for the reaction; THF yields multiple by-products along with the desired product. We shortened the reaction time to 6 h while still preserving high yields. **4** was obtained as sticky yellow paste (0.21 g from 0.23 g of 3; 94%). ^{1}H NMR (500 MHz, $CDCl_3$) δ (ppm): 7.27 (s, 1H), 7.26 (s, 1H), 6.09 (d, J = 3.6 Hz, 2H), 4.78 (q, J = 7.0 Hz, 1H), 3.68-3.57 (m, 2H), 2.62-2.48 (m, 2H), 1.55 (d, J = 7.0 Hz, 3H). ^{13}C NMR (126 MHz, $CDCl_3$) δ (ppm): 152.3 (C), 146.9 (C), 144.0 (C), 136.0 (C), 108.0 (CH), 104.8 (CH), 103.0 (CH_2), 60.9 (CH_2), 38.3 (CH), 34.9 (CH_2), 23.2 (CH_3).

2-{[1-(6-nitrobenzo[d][1,3]dioxol-5-yl)ethyl]thio}ethyl(4-nitrophenyl)carbonate **5**. Instead of preparing the succinimidyl carbonate as previously reported[5], we switched to activating **4** as a 4-nitrophenyl carbonate, as the latter is easy to monitor through TLC and is known to be stable upon silica gel chromatography.

A solution of **4** (0.2897 g, 1.06 mmol, 1 eq) in dry CH_3CN was added to an oven-dried three-necked round-bottomed flask. Triethylamine (0.6 mL, 4.30 mmol, 4.05 eq) was added to the flask, followed by 4-Nitrophenyl chloroformate (0.3598 g, 1.78 mmol, 1.68 eq). The reaction turned turbid and white precipitates were formed. The solution was then left stirring at r.t. for 6 h. The reaction was judged to be complete by TLC (3:7 EtOAC:hexanes) and was evaporated to dryness under reduced pressure. The product was purified by column chromatography on silica (eluent: 5% to 30% EtOAc:hexanes) in the dark to obtain **5** as yellow gum (0.2853 g, 65%) with an R_f = 0.48 (silica plate, EtOAc/hexanes = 3:7). ^{1}H NMR (500 MHz, $CDCl_3$) δ (ppm): 8.27 (d, *J* = 9.2 Hz, 2H), 7.38 (d, *J* = 9.1 Hz, 2H), 7.30 (s, 1H), 7.27 (s, 1H), 6.09 (s, 2H), 4.87 (q, *J* = 7.0 Hz, 1H), 4.32 (qt, *J* = 11.1, 6.6 Hz, 2H), 2.77 – 2.63 (m, 2H), 1.56 (s, 3H). ^{13}C NMR (126 MHz, $CDCl_3$) δ (ppm): 155.6, 152.3, 152.1, 146.8, 145.5, 143.4, 135.7, 125.4, 121.9, 108.0, 104.9, 103.1, 68.0, 39.0, 31.0, 23.1.

tert-Butyl(2S)-2-[(tert-Butoxycarbonyl)amino]-3-{[(2-{[1-(6-nitrobenzo[d][1,3]dioxol-5- yl)ethyl]thio}ethoxy)carbonyl]amino}propanoate **6**. **5** (0.2853 g, 0.65 mmol, 1 eq) was dissolved in dry CH_3CN (8 mL) and transferred into a three-necked round-bottom flask. Boc-Dap-OH (0.1460 g, 0.71 mmol, 1.09 eq) was added in one portion to the flask, followed by addition of triethylamine (0.55 mL, 3.94 mmol, 6.06 eq). The reaction flask was wrapped with aluminium foil and stirred at rt for 6 h under N_2 atmosphere. After this time, the reaction was judged to be completed by TLC (98:2 EtOAc:CH_3COOH), then dried under reduced pressure. The resulting residue was purified by silica column chromatography (eluent: 50:50 EtOAc: hexanes to 100% EtOAc then 5%>40% MeOH:EtOAc) in the dark to obtain **6** as yellow gum (0.3098 g, 94 %). ^{1}H NMR (500 MHz, $CDCl_3$) δ (ppm): 7.28 (s, 1H), 7.25 (s, 1H), 6.09 (d, *J* = 15.1 Hz, 2H), 5.99 (s, 1H), 5.86 (s, 1H), 4.78 (q, *J* = 7.0 Hz, 1H), 4.17 (s, 1H), 4.07 – 4.01 (m, 2H), 3.51 (s, 2H), 2.51 (tq, *J* = 13.3, 6.7 Hz, 2H), 1.51 (d, *J* = 6.9 Hz, 3H), 1.38 (s, 9H). ^{13}C NMR (126 MHz, $CDCl_3$) δ (ppm): 173.7 (C), 158.0 (C), 156.5 (C), 152.1 (C), 146.9 (C), 143.3 (C), 136.2 (C), 108.1 (CH), 104.8 (CH), 103.1 (CH_2), 81.8 (C), 64.6 (CH_2), 54.4 (CH), 43.2 (CH_2), 38.9 (CH), 32.0 (CH_2), 28.4 (CH_3).

(2S)-2-[(tert-Butoxycarbonyl)amino]-3-{[(2-{[1-(6-nitrobenzo[d][1,3]dioxol-5-yl)ethyl] thio}ethoxy)carbonyl]amino}propanoic acid **7**. We followed the protocol described in Huguenin-Dezot *et al.*[5] and obtained **7** as pale yellow powder (0.19 g from 0.50 g of **6**; 48%). ^{1}H NMR (500 MHz, $CDCl_3$) δ (ppm): 7.31 (s, 1H), 7.27 (s, 1H), 6.11 (d, J = 4.5 Hz, 2H), 4.96 (dt, J = 11.8, 6.4 Hz, 56H), 4.73 (q, J = 6.9 Hz, 1H), 4.15 – 3.98 (m, 3H), 3.69 (ddd, J = 14.9, 5.1, 3.9 Hz, 1H), 3.54 (dd, J = 15.1, 6.2 Hz, 2H), 2.66 – 2.47 (m, 2H), 1.52 (d, J = 6.9 Hz, 3H). ^{13}C NMR (126 MHz, CD_3OD) δ (ppm): 168.5 (C), 161.4 (C), 160.0 (C), 152.1 (C), 147.1 (C), 141.0 (C), 135.4 (C), 115.8 (C), 107.4 (CH), 104.0 (CH), 103.3 (CH_2), 64.3 (CH_2), 53.6 (CH), 40.5 (CH_2), 38.4 (CH), 29.9 (CH_2), 21.7 (CH_3).

Bioinformatic workflow to identify putative PET-degrading enzymes

We retrieved amino acid sequences of known PET hydrolases from UniProt. The amino acid sequence of *Is*PETase was used as a query for HMMER search in MGnify non-redundant protein with the default options in May/2019. The sequence of *Is*PETase was aligned with the downloaded sequences from MGnify using the local version of MUSCLE 3.8.31. The aligned sequences were filtered using Biopython script. The filtered sequences together with the known PET hydrolase sequences were used to generate a sequence similarity network by EFI-EST. The generated network is visualized by Cytoscape 3.8.2.

The candidates for experimental validation were manually selected based on the HMMER search results in the MGnify non-redundant protein database with the marine or human database option. The selected candidates were subjected to secondary structure prediction by PSIPRED, signal peptide prediction by SignalP5.0, and putative host search by BLASTP.

Homology modelling and electrostatic potential calculations

Homology modeling of MG1 to MG10 was performed using SWISS-MODEL[6]. PDB files obtained from the modeling or Protein Data Bank were optimized by PDB2PQR (ver. 3.5.1) before the electrostatic potential calculation using APBS (ver. 3.0.0) on APBS web service[7]. Structures were rendered by using UCSF ChimeraX[8]. We used the following template information for the modelling: PDB 6SCD for MG1, MG2, MG5, MG7, and MG8; PDB 7EC8 for MG3 and MG6; PDB 7ECB for MG4; PDB 7NEI for MG9; and PDB 3VIS for MG10.

Molecular cloning

Constructs used in this study were summarized in Table S4; primer sequences used for their cloning were summarized in Table S5.

Each of the ten candidate genes for putative PET-degrading enzymes, MG8-GFP11 and GFP1-10 were custom-synthesized by Twist Bioscience and subcloned into a pET21a expression vector; their sequence information is provided in Table S4. For MG8, site-directed mutagenesis was performed on its F250 residue, converting the residue to A, S, V, I, and L using primers provided in Table S5. To delete the RYD loop of MG8 and add RYD to *Is*PETase, we amplified corresponding segments and used Gibson Assembly, using primers provided in Table S5. Genes for *Is*PETase (S121E/D186H/R280A, also known as ThermoPETase) and DuraPETase (A214H/I168R/W159H/S188Q/R280A/A180I/G165A/Q119Y/L17F/T140D), both also containing a G11 tag for split-GFP reconstitution, were custom-synthesized by Twist Bioscience and subcloned into a pET21b expression vector. For the expression of 2,3-diaminoproprionic acid (DAP)-incorporated MG8, we subcloned the MG8-GFP11 tag sequence into pNHD1.3-His_6-lipoyl-TEV-Strep[5] via Gibson assembly. The catalytic serine residue of MG8, S171, was then converted to an amber stop codon (TAG) by site-directed mutagenesis to produce pNHD1.3-His_6-MG8(S171TAG)-GFP11 tag. The pNHD1.3-His_6-MG8(S171TAG)-GFP11 tag plasmid was used in combination with pMB1-DapRS-PylT (a gift from Jason Chin), the latter of which provides *E.coli* expression of photocaged DAP-specific *Methanosarcina barkeri* pyrrolysyl-tRNA synthetase and its cognate amber-suppressor pyrrolysyl tRNA (PylT), for genetic code expansion experiments. All plasmids used in this study were sequence-confirmed by Sanger sequencing. To fuse MG8 with the MBP tag (pMBP-MG8) for expression of MG8 under non-denaturing conditions, the MG8 gene was amplified by PCR with primers provided in Table S5. The amplified gene was subcloned into pMBP-LbCas12a (Addgene plasmid #113431) doubly digested with NdeI and BamHI using Gibson assembly.

Protein expression and purification

To express putative PET-degrading enzymes, a PET21a plasmid containing sequences for one of the ten putative PET-degrading enzymes was transformed into *E.coli* BL21(DE3) and cultured in Lysogeny broth (LB) supplemented with 100 μg/ml Ampicillin at 37 °C, 220 rpm until OD_{600} reached 0.6-0.8 Isopropyl β-D-1-thiogalactopyranoside (IPTG) was then added to the final concentration of 1 mM, and the culture growth was continued at 37 °C, 220 rpm for 4 h. Cells (~2 g) were harvested, resuspended in lysis buffer (25 mM Tris-HCl, 500 mM NaCl, 1% Triton-X, and 1 mM DTT at pH 7.5), and lyzed by ultrasonication (Sonics Vibracell VCX750) using 220-B probe and 40% pulse amplitude for 3 min. The cell debris was separated by centrifugation at 15,000 rpm for 10 min and the supernatant discarded; the centrifugation and supernatant removal were repeated two more times. The resulting pellet was resuspended with 15 mL denaturing binding buffer (25 mM Tris-HCl, 500 mM NaCl, and 6 M urea at pH 7.5). The resulting suspension was subject to affinity chromatography purification under a denaturing condition, as follows: the lysate-binding buffer mix was loaded into Chelating Sepharose™ Fast Flow column (GE Healthcare) which was pre-loaded with 0.2 M Nikel sulphate ($NiSO_4$) and pre-equilibrated with the denaturing binding buffer; washed with denaturing binding buffer for 5 column volumes (CV); washed with denaturing binding buffer supplemented with 30 mM imidazole for 10 CV; and eluted with denaturing binding buffer supplemented with 350 mM imidazole for 5 CV. Eluted denatured protein fractions were pooled based on SDS-PAGE analysis and refolded by dialysis with urea-free binding buffer containing 200 mM L-arginine to remove imidazole and urea. Purified proteins were dialyzed into storage buffer (25 mM Tris-HCl, pH 7.5, 150 mM NaCl), snap-frozen with liquid nitrogen, and stored at -80 °C. We normalized protein concentrations based on band densitometry in SDS-PAGE analysis upon using them in enzymatic activity assays.

To express DAP-incorporated MG8, pNHD1.3-His_6-MG8(S171TAG) and pMB1-DapRS-PylT plasmids were co-transformed into *E.coli* BL21(DE3) and cultured at 1 L volume in LB supplemented with 100 μM photocaged DAP, 50 μg/ml streptomycin and 10 μg/ml tetracycline in the dark at 37 °C, 220 rpm. When culture OD_{600} reached ~0.6, IPTG was added at 1 mM (final concentration), and the cultures grown for further 20 h at 37 °C, 220 rpm in the dark. Thereafter, cells were harvested and lyzed as previously described, but in the dark. The lysate was subject to denaturing Ni affinity chromatography purification as previously described (also in the dark). The eluted protein fractions were pooled based on SDS-PAGE analysis and irradiated with UV light (CAMAG® UV lamp 4 dual wavelength) at 366 nm, 5.2 mW/cm^2 for 10 min to initiate photouncaging of DAP. After irradiation, the protein solution was dialyzed in 25 mM Tris-HCl, 500 mM NaCl, 5 mM DTT, 6 M urea and 350 mM imidazole at an elevated pH of 8.5 for 24 h to promote complete deprotection of DAP. After the deprotection promotion step, the denaturing protein was refolded as the procedure above. The protein solution was further dialyzed into 25 mM Tris-HCl and 150 mM NaCl at pH 7.5, snap-frozen with liquid nitrogen, and stored at -80 °C.

To express *Is*PETase and its variants under non-denaturing conditions, pET21b-*Is*PETase (Addgene plasmid # 112202), pET21b-*Is*PETase (W159H/S238F, Addgene plasmid # 112203) or another pET21b-*Is*PETase variant-expressing plasmid was transformed into BL21(DE3), and the expression and purification were performed as previously reported[3]. To express GFP1-10, pET21a-GFP1-10 was transformed into BL21(DE3), and the protein harvested and purified as previously reported[9].

To express MG8 under non-denaturing conditions, pMBP-MG8 plasmid was transform into *E. coli* BL21 (DE3) and cultured at 4 L volume in LB supplemented with 100 µg/ml ampicillin at 37 °C, 220 rpm then OD600 reached 0.6-0.8, IPTG was added at 1 mM (final concentration), and the cultures grown for further 16 h at 18 °C. Cells was collected by centrifugation at 8,000 rpm for 20 min at 4 °C. The supernatant was discarded, and the cell pellet was resuspended in extraction buffer (25 mM Tris-HCl, 500 mM NaCl, and 5 mM imidazole; pH 7.5). The cell resuspension was lysed by sonication using 40% pulse amplitude (on 5 s and off 10 s) until completely lysed. The lysate was centrifuged at 15,000 rpm for 30 min at 4 °C. The resulting supernatant was applied to Chelating Sepharose™ Fast Flow column which was pre-loaded with 0.2 M $NiSO_4$ and pre-equilibrated with binding buffer (25 mM Tris-HCl, 500 mM NaCl, 5 mM imidazole; pH 7.5). The column was washed with 5 CV of binding buffer, 10 CV of washing buffer (25 mM Tris-HCl, 500 mM NaCl, 30 mM imidazole; pH 7.5) and eluted with 7 CV of elution buffer (25 mM Tris-HCl, 250 mM NaCl, 500 mM imidazole; pH 7.5). The eluted fractions containing His-MBP-MG8 were pooled and a fusion tag removed with UltraTEV protease using 20:1 of TEV substrate (50 mM Tris-HCl, 0.5 mM EDTA, 1 mM DTT, 5% Glycerol; pH 7.5). The reaction mixture was gently agitated at 20 °C overnight, diluted with 50 mM sodium phosphate pH 6.0 to conductivity around 3-5 mS/cm, then adjusted to pH 6.0 with 1 M phosphoric acid. Subsequently, the protein was applied to a HiTrap SP FF column, which was pre-equilibrated with binding buffer (50 mM sodium phosphate pH 6.0). The column washed with 10 CV of binding buffer and eluted with 10 CV of 50 mM sodium phosphate, 50 mM sodium chloride pH 6.0. The purified MG8 fractions were pooled, concentrated, and exchanged against 25 mM Tris-HCl, 150 mM NaCl, pH 7.5 with 10k Amicon (Merk Millipore) and stored at -80 °C.

To express *Thermobifida fusca* cutinase (Tfu) and *Humicola insolens* cutinase (HiC), the pRSFDuet-1 harbouring Tfu and HiC sequences was transformed into *E.coli* BL21(DE3) and C41(DE3), respectively. They were cultured in LB supplemented with 50 µg/ml Kanamycin at 37 °C, 250 rpm until OD_{600} reached 0.7-0.8. IPTG was then added to the final concentration of 0.2 mM, and the culture growth was continued at 16 °C, 130 rpm for 24 h. The cells were harvested by centrifuge at 10,000 xg for 15 min at 4 °C, resuspended in lysis buffer (50 mM sodium phosphate, 500 mM NaCl, 10 mM Imidazole; pH 8.0) and lyzed by ultrasonication using 80% pulse amplitude for 6 min at 12 °C. The cell debris was separated by centrifugation at 15,000 rpm for 30 min. The resulting supernatant was loaded into nickel-nitriloacetic acid (Ni-NTA) based affinity chromatography purification, as follows: the lysate-binding buffer mix was loaded into nickel bead placed in the column and pre-equilibrated with the lysate-binding buffer; washed with lysate-binding buffer supplemented with 20 mM Imidazole for 10 CV and eluted with lysate-binding buffer supplemented with 250 mM Imidazole for 5 CV. Eluted protein fractions were pooled based on SDS-PAGE analysis, dialyzed into storage buffer (25 mM Tris-HCl, 150 mM NaCl, 5 mM BME, 20% glycerol; pH 7.5) and stored at -80 °C.

Assessing esterase activity of putative PET-degrading enzymes using p-nitrophenyl acetate (pNpA) under different pH and salt concentrations

A 100 mM pNpA stock solution in methanol was prepared. To set up reactions, 5 mM pNpA was incubated with 300 nM purified enzyme (concentrations normalized based on protein purity observed on SDS-PAGE) in buffers in varying pH (4.5-9.5) and NaCl concentrations (0-5 M). The reactions were set at 50-µL volume, and pNpA hydrolysis was monitored at 30 °C through absorbance measurements at 405 nm over 12 min using MULTISKAN Sky microplate reader. Background-subtracted absorbance values were calculated using background absorbance from a negative control, with enzyme omitted, measured in the same experiment.

Specifically, for Figure S8a, we used Solubility & Stability Screen 2™ kit (supplied in a 96-well plate format) from Hampton Research at 4-fold dilutions ratio to generate buffers with different pHs and 0.05-1 M NaCl. We performed the pNpA assays for these 96 conditions. For Figure 1e and Figure S8b, we tested further reaction conditions: the pH of the reactions was narrowed to the neutral/basic range of pH 7, 7.5, 8, or 9; we prepared these buffers using 50 mM Tris-HCl. NaCl concentrations were tested at 1-5 M, which is a higher range than what was provided in the Solubility & Stability Screen 2 kit.

Assessing PET hydrolase activity using a PET surrogate substrate, bis(2-hydroxyethyl) terephthalate (BHET)

150 mM BHET stock solutions were prepared by dissolving BHET in dimethyl sulfoxide. To set up 500 µL BHET hydrolysis reactions, 3 mM BHET was incubated with 500 nM purified enzyme in 50 mM Tris-HCl pH 8 and NaCl concentrations (0.15, 1, or 4 M) at 30, 37, 45, 55 or 65 °C for 30 min. Reactions were quenched with phenylmethylsulfonyl fluoride (PMSF, final concentration 5 mM), diluted 2-fold with 5 mM phosphate pH 7.0, filtered using 0.22 µm filter (FilterBio®, Nylon Syringe filter), and analyzed on an Agilent 1260 Infinity II HPLC equipped with a reverse-phase Agilent Eclipse Plus C18 column (4.6 x 250 mm, 5 µm), heated in a column oven to 30 °C. We used a 12-min isocratic gradient of 27% acetonitrile in water at 0.6 mL/min flow rate, then cleaned the column with 80% acetonitrile in water for another 5 min. In these reactions, we specifically monitored three analytes, the starting material BHET, and the hydrolysis products mono(2-hydroxyethyl) terephthalate (MHET) and terephthalic acid (TPA). We generated calibration curves for BHET and TPA using commercial standards. BHET and TPA concentrations used for calibration curves were 3, 2, 1, 0.1 and 0.01 mM. MHET used for its calibration curve was generated *in situ* using *Is*PETase-mediated hydrolysis of BHET; BHET was completely hydrolyzed by *Is*PETase to 99% MHET and 1% TPA, as previously reported[10]. MHET concentrations used for calibration curves were also 3, 2, 1, 0.1 and 0.01 mM.
For Figure S9, we tested the hydrolytic activity of enzymes toward BHET under different NaCl concentrations, at a fixed temperature of 37 °C. For Figure S10, we tested the hydrolytic activity of enzymes toward BHET under different temperatures (30, 37, 45, 55 and 65 °C), at a fixed NaCl concentration of 4 M.

Assessing PET hydrolase activity using PET powder

We characterized PET hydrolase activity of four selected enzyme candidates (MG1, MG7, MG8 and MG10) using PET powder as a substrate. 20 mg PET powder was incubated with 500 nM purified enzyme in 50 mM Tris-HCl and 4 M NaCl at pH 8 or 9, at 30, 37, 45, 55 or 65 °C for 48 h. 500 µL of reactions were quenched with PMSF (final concentration 5 mM) and pH-adjusted to pH 7.5 using 160

mM phosphate pH 2.5 solution. After centrifugation, the supernatant was transferred to a new Eppendorf tube, and diluted 2-fold with 5 mM phosphate pH 7.0, filtered using 0.22 µm filter, and analyzed with HPLC using the same HPLC method as described above.
For Figure 2b and Figure S11, we tested the hydrolytic activity of enzymes toward PET powder under different temperatures (30, 37, 45, 55 and 65 °C), at a fixed NaCl concentration of 4 M. For Figure S13, we compared MG8 and *Is*PETase for their PET powder degradation activities under different reaction conditions: varying NaCl concentrations (0.15, 1, 2, 3, or 4 M) and pH (8 or 9), at a fixed temperature of 55 °C. For Figure 2i and Figure S22, we compared PET powder degradation activity of different MG8 mutants (F250A, F250I, F250L, F250S and F250V) under a fixed condition of pH 9, 4 M NaCl and 55 °C, which we found to be optimal for the activity of wild-type MG8.

For Figure 3a and Figure S14, we compared PET powder degradation activity of MG8 and *Is*PETase variants--*Is*PETase (W159H/S238F), ThermoPETase, and DuraPETase--under two reaction temperatures (37 and 55 °C), at pH 9.0 and 4 M NaCl for 48 h. All enzymes used for this comparison were freshly prepared during the same period, using non-denaturing purification conditions.

Kinetic measurements

We measured the reactions rate at difference enzyme concentrations (50, 100, 150, 200, 300, 400, and 500 nM). All reactions were performed in triplicate using 15 mg PET powder in 400 µl with the same buffer (50 mM Glycine-NaOH, 4 M NaCl; pH 9.0) in 96 deep-well plates (sealed plate with tape during incubation). The reaction plates were placed on a shaking incubator at 250 rpm at 37 or 55 ℃. The reaction supernatant was transferred every 2 h to a 96-well microplate using a multi-channel pipette, for absorbance at 260 nm (A_{260}) measurements on a MULTISKAN Sky microplate reader (Thermo scientific). When absorbance values approached the maximal detection range of the instrument, the supernatant was diluted 10-fold prior to the measurement. The supernatant was then transferred back to the deep-well plate for more incubation with PET powder until the next time point. TPA and MHET standards (1, 0.5, 0.1, 0.05, 0.005 mM) were used to generate A_{260} calibration curves.

Assessment of thermal stability of PET-degrading enzymes

We characterized the melting temperature of enzymes of interest using the Thermofluor assay. Sypro orange (10x final concentration; 5000x stock solution from Thermo Fisher) was mixed with purified enzyme (7.5 µM final concentration) in 25 mM Tris-HCl and 150 mM NaCl at pH 7.5; the total volume of the mixture was 25 µl. Melting curve analyses were performed according to a guideline “Protein thermal shift assays made easy” from Bio-Rad, on a Bio-Rad CFX Connect Real-time PCR detection system.

MALDI-TOF analyses of intact protein masses

The MG8 enzyme with different states of DAP modifications was precipitated using the methanol-chloroform-water (MCW) method, as follows. 100 µL of the protein solution (7.2 µM in storage buffer) was added to an eppendorf tube, followed by sequential addition of 400 µL methanol, 100 µL chloroform and 300 µL molecular biology-grade water, and with vortexing after each addition step. The mixture was centrifuged at 14,000 xg for 1 min to expedite layer separation; proteins generally precipitated as flakes at the aqueous-organic interface. The top, aqueous phase was discarded and 400 µL methanol was added to the remaining mixture, followed by vortexing. The mixture was centrifuged at 20,000 xg for 5 min. After the supernatant was removed, the resulting protein pellet was air-dried and stored at -20 °C. Prior to MALDI-TOF analysis, 5 µL of 80% formic acid in water was added to resuspend the protein pellet, followed by incubation in -20 °C for 2 min, mixing by pipetting, and incubation in -20 °C for another 8 min. Lastly, the solution was diluted 10-fold with 45 µL molecular biology-grade water. The protein solution was further diluted 2-fold with 100% acetonitrile.

Sinapinic acid (12.5 mg/ml in 50% acetonitrile/water and 0.1% trifluoroacetic acid) was used as a matrix. We mixed the protein solution and the matrix at 3:7 protein:matrix volume ratio. 0.5 µL of the resulting solution was dropped in each sample well and air-dried. The resulting dried crystals of the mixed solution between proteins and the matrix were analyzed on a MALDI-TOF mass spectrometer (MALDI-TOF-MS/MS, Bruker, Autoflex speed™ system) with ion source 1: 19.50 kV; ion source 2: 17.75kV and 50 Hz smartbeam-II laser. The spectra were obtained in positive linear mode. The conditions were set as 90% laser power, 10 shots at raster spot and 800 µm limit diameter.

GFP functionalization on plastic surface

10 mg of plastic powder (PET, PBS, PCL, or PLA) was incubated with 7.59 µM GFP11-tagged MG8(S171DAP) in 50 mM Tris-HCl and 4 M NaCl at pH 9 and 50 °C for 1 h. Alternatively, GFP11-tagged MG8(S171DAP) was inactivated by incubation with 5 mM PMSF for 40 min prior to addition to plastic powder. Thereafter, the powder was washed with 1 mL of 1x TBST three times, and further incubated with 500 µL of 49 µM GFP1-10 in the presence of 5 mg/mL bovine serum albumin (BSA) at 37 °C for 24 h. The powder was washed three times with TBST and then resuspended with storage buffer (25 mM Tris-HCl, 150 mM NaCl; pH 7.5).

Fluorescence imaging and quantification of GFP fluorescence

Plastic powder was imaged using either an Amersham™ ImageQuant 800 Fluor biomolecular imager or an Olympus Fluoview FV3000 confocal microscope. To provide better contrast of GFP signal over intrinsic fluorescence of plastic powder, we merged Cy2-channel images (colorized as green, 488/525 nm) with Cy3-channel images (colorized as red, 365/605 nm). For confocal microscopy, resuspended plastic powder was transferred to a 12-mm-diameter glass-base dish, then imaged with a 20x/0.8 NA objective lens.

For fluorescence quantification, resuspended plastic powder was transferred to a 96-well black polystyrene microplate. Fluorescence signals were measured using a Tecan infinite 200 PRO microplate reader at excitation and emission wavelengths of 488 and 522 nm, respectively.

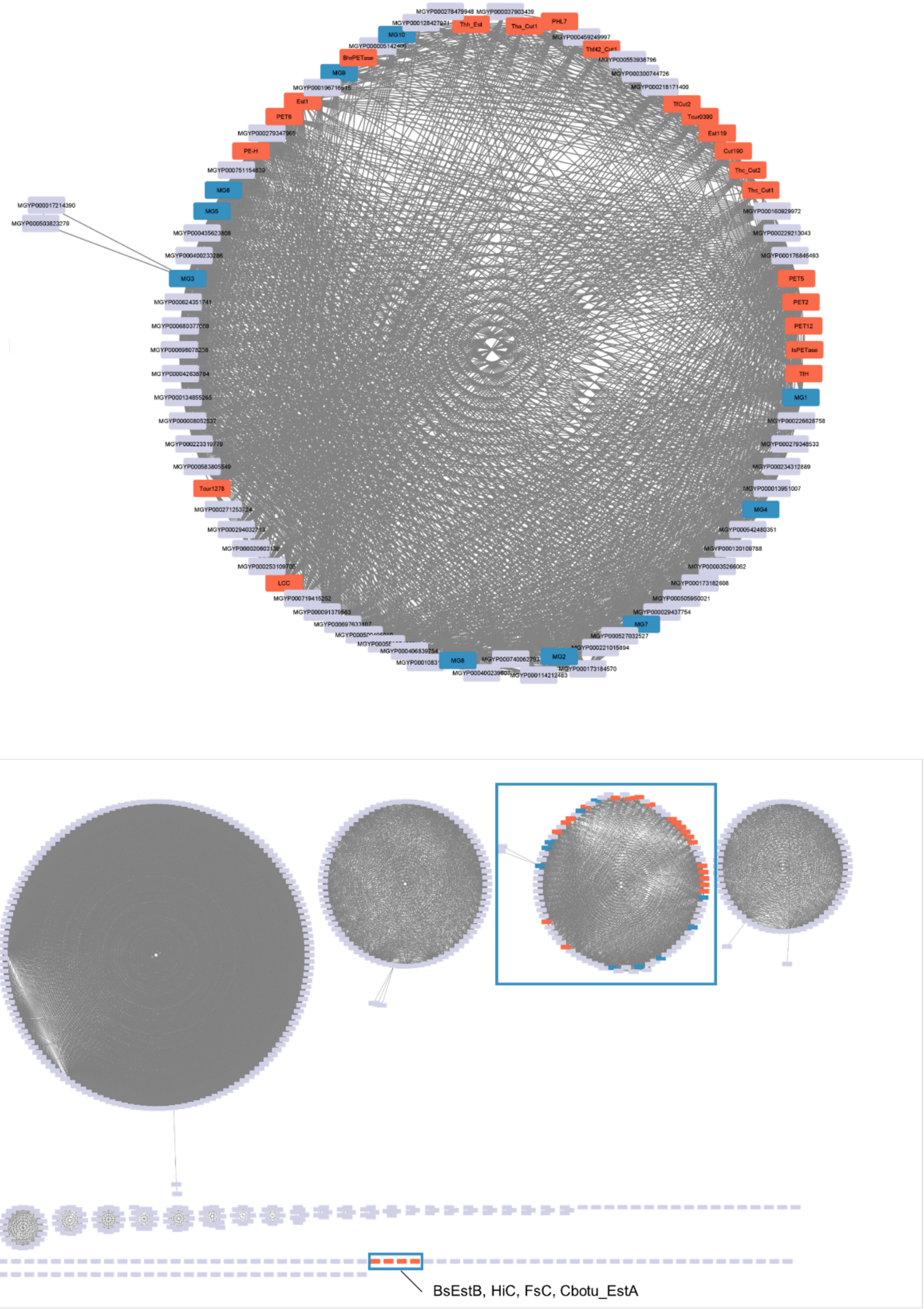

Figure S1. A sequence similarity network of putative and known PET hydrolases. The putative PET hydrolases used to generate this network all contain required catalytic triad residues (S/H/D) of serine hydrolases. Known PET hydrolases are labeled in red; PET hydrolase candidates of our highest interest, named MG1 to MG10, are labeled in dark blue; all other PET hydrolase candidates are labeled in light blue. Except for *Bacillus subtilis* p-nitrobenzylesterase (BsEstB), *Humicola insolens* cutinase (HiC), *Fusarium solani* cutinase (FsC), and *Clostridium botulinum* esterase (Cbotu_EstA) other known PET hydrolases belong to the same cluster as MG1-MG10.

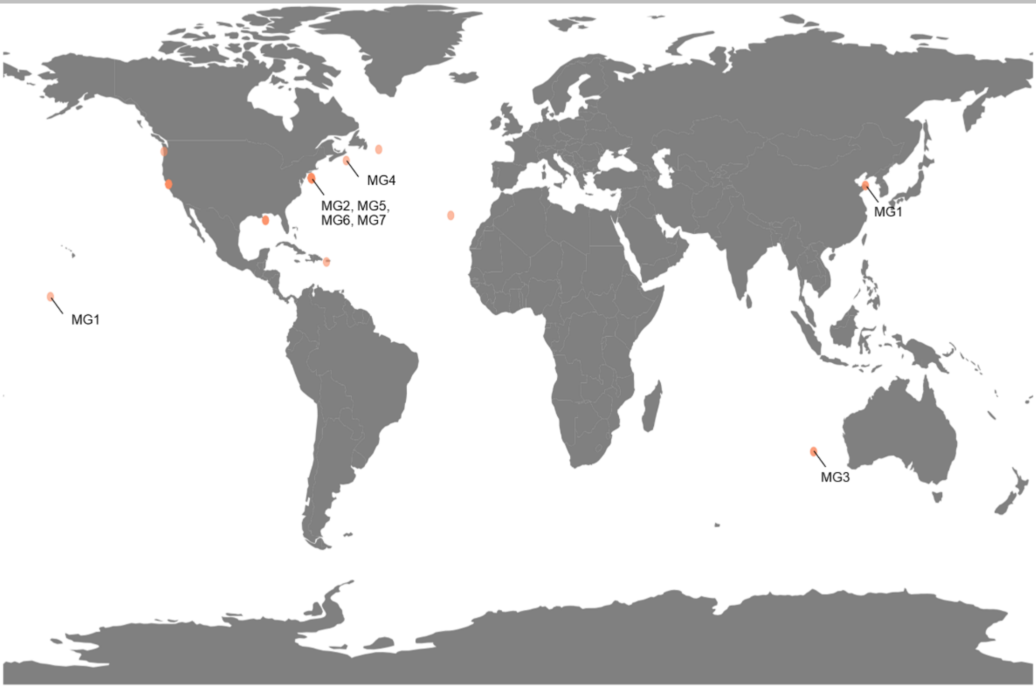

Figure S2. The geographical distribution of putative PET hydrolases from the MGnify database. Shown in this map are sample collection locations of microbiomes of marine origin, from where a subset of putative PET hydrolase sequences—those belong to the same cluster of the sequence similarity network (Figure 1) as the majority of known PET hydrolases—can be retrieved.

MG1

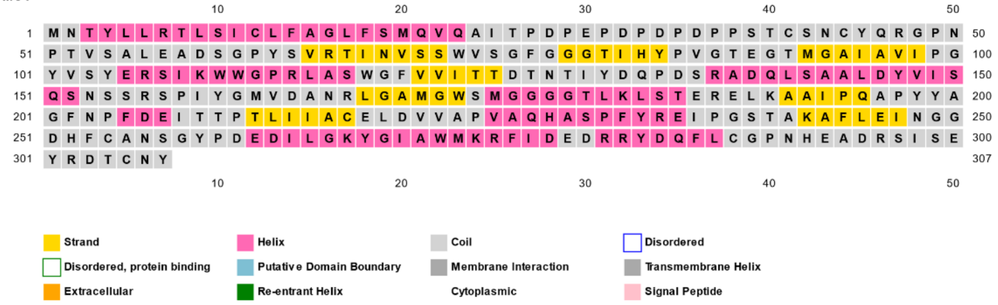
10 20 30 40 50
1 MNTYLLRTLSICLFAGLFSMQVQAITPDPEPDPDPDPPSTCSNCYQRGPN 50
51 PTVSALEADSGPYSVRTINVSSWVSGFGGGTIHYPVGTEGTMGAIAVIPG 100
101 YVSYERSIKWWGPRLASWGFVVITTDTNTIYDQPDSRADQLSAALDYVIS 150
151 QSNSSRSPIYGMVDANRLGAMGWSMGGGGTLKLSTERELKAAIPQAPYYA 200
201 GFNPFDEITTPTLIIACELDVVAPVAQHASPFYREIPGSTAKAFLEINGG 250
251 DHFCANSGYPDEDILGKYGIAWMKRFIDEDRRYDQFLCGPNHEADRSISE 300
301 YRDTCNY 307
10 20 30 40 50
Strand
Helix
Coil
Disordered
Disordered, protein binding
Putative Domain Boundary
Membrane Interaction
Transmembrane Helix
Extracellular
Re-entrant Helix
Cytoplasmic
Signal Peptide

MG2

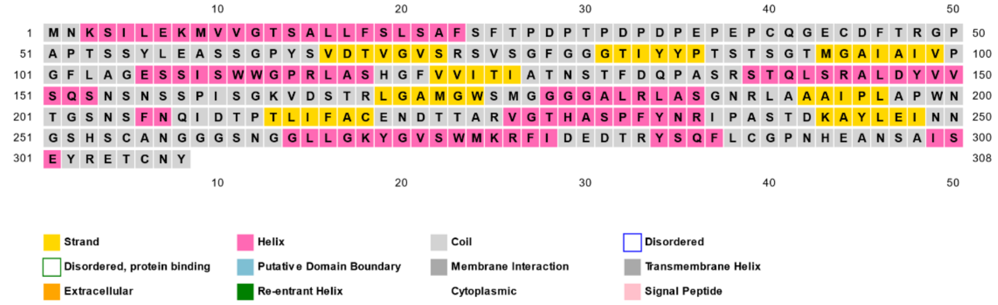
10 20 30 40 50
1 MNKSILEKMVVGTSALLFSLSAFSFTPDPTPDPDPEPEPCQGECDFTRGP 50
51 APTSSYLEASSGPYSVDTVGVSRSVSGFGGGTIYYPTSTSGTMGAIAIVP 100
101 GFLAGESSISWWGPRLASHGFVVITIATNSTFDQPASRSTQLSRALDYVV 150
151 SQSNSNSSPISGKVDSTRLGAMGWSMGGGGALRLASGNRLAAAIPLAPWN 200
201 TGSNSFNQIDTPTLIFACENDTTARVGTHASPFYNRIPASTDKAYLEINN 250
251 GSHSCANGGGSNGGLLGKYGVSWMKRFIDEDTRYSQFLCGPNHEANSAIS 300
301 EYRETCNY 308
10 20 30 40 50
Strand
Helix
Coil
Disordered
Disordered, protein binding
Putative Domain Boundary
Membrane Interaction
Transmembrane Helix
Extracellular
Re-entrant Helix
Cytoplasmic
Signal Peptide

MG3

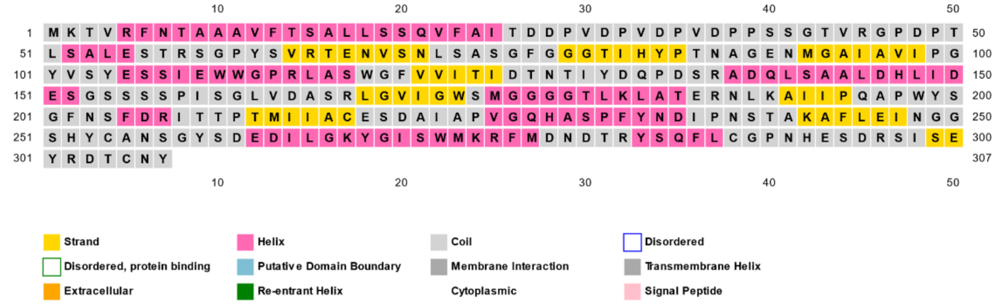
10 20 30 40 50
1 MKTVRFNTAAAVFTSALLSSQVFAITDDPVDPVDPVDPPSSGTVRGPDPT 50
51 LSALESTRSGPYSVRTENVSNLSASGFGGGTIHYPTNAGENMGAIAVIPG 100
101 YVSYESSIEWWGPRLASWGFVVITIDTNTIYDQPDSRADQLSAALDHLID 150
151 ESGSSSSPISGLVDASRLGVIGWSMGGGGTLKLATERNLKAIIPQAPWYS 200
201 GFNSFDRITTPTMIIACESDAIAPVGQHASPFYNDIPNSTAKAFLEINGG 250
251 SHYCANSGYSDEDILGKYGISWMKRFMDNDTRYSQFLCGPNHESDRSISE 300
301 YRDTCNY 307
10 20 30 40 50
Strand
Helix
Coil
Disordered
Disordered, protein binding
Putative Domain Boundary
Membrane Interaction
Transmembrane Helix
Extracellular
Re-entrant Helix
Cytoplasmic
Signal Peptide

MG4

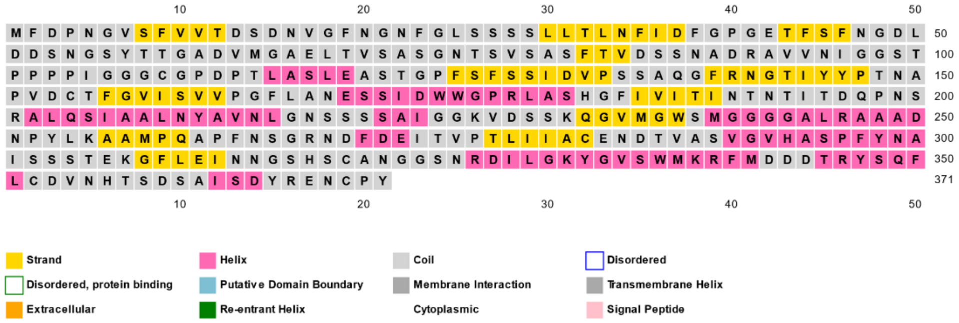
10 20 30 40 50
1 MFDPNGVSFVVTDSDNVGFNGNFGLSSSSLLTLNFIDFGPGETFSFNGDL 50
51 DDSNGSYTTGADVMGAELTVSASGNTSVSASFTVDSSNADRAVVNIGGST 100
101 PPPPIGGGCGPDPTLASLEASTGPFSFSSIDVPSSAQGFRNGTIYYPTNA 150
151 PVDCTFGVISVVPGFLANESSIDWWGPRLASHGFIVITINTNTITDQPNS 200
201 RALQSIAALNYAVNLGNSSSSAIGGKVDSSKQGVMGWSMGGGGALRAAAD 250
251 NPYLKAAMPQAPFNSGRNDFDEITVPTLIIACENDTVASVGVHASPFYNA 300
301 ISSSTEKGFLEINNGSHSCANGGSNRDILGKYGVSWMKRFMDDDTRYSQF 350
351 LCDVNHTSDSAISDYRENCPY 371
10 20 30 40 50
Strand
Helix
Coil
Disordered
Disordered, protein binding
Putative Domain Boundary
Membrane Interaction
Transmembrane Helix
Extracellular
Re-entrant Helix
Cytoplasmic
Signal Peptide

MG5

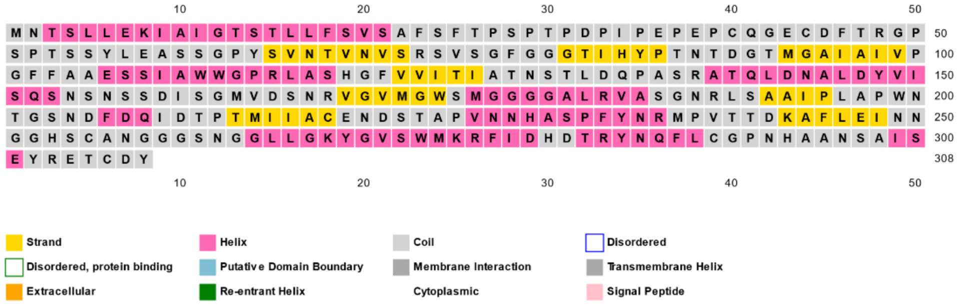
10 20 30 40 50
1 MNTSLLEKIAIGTSTLLFSVSAFSFTPSPTPDPIPEPEPCQGECDFTRGP 50
51 SPTSSYLEASSGPYSVNTVNVSRSVSGFGGGTIHYPTNTDGTMGAIAIVP 100
101 GFFAAESSIAWWGPRLASHGFVVITIATNSTLDQPASRATQLDNALDYVI 150
151 SQSNSNSSDISGMVDSNRVGVMGWSMGGGGALRVASGNRLSAAIPLAPWN 200
201 TGSNDFDQIDTPTMIIACENDSTAPVNNHASPFYNRMPVTTDKAFLEINN 250
251 GGHSCANGGGSNGGLLGKYGVSWMKRFIDHDTRYNQFLCGPNHAANSAIS 300
301 EYRETCDY 308
10 20 30 40 50
Strand
Helix
Coil
Disordered
Disordered, protein binding
Putative Domain Boundary
Membrane Interaction
Transmembrane Helix
Extracellular
Re-entrant Helix
Cytoplasmic
Signal Peptide

MG6

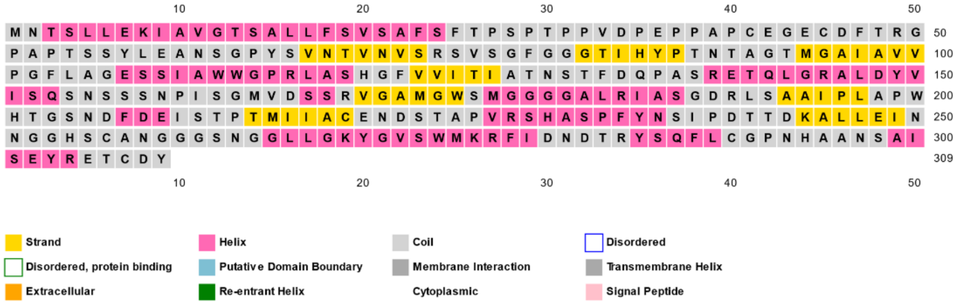
10 20 30 40 50
1 MNTSLLEKIAVGTSALLFSVSAFSFTPSPTPPVDPEPPAPCEGECDFTRG 50
51 PAPTSSYLEANSGPYSVNTVNVSRSVSGFGGGTIHYPTNTAGTMGAIAVV 100
101 PGFLAGESSIAWWGPRLASHGFVVITIATNSTFDQPASRETQLGRALDYV 150
151 ISQSNSSSNPISGMVDSSRVGAMGWSMGGGGALRIASGDRLSAAIPLAPW 200
201 HTGSNDFDEISTPTMIIACENDSTAPVRSHASPFYNSIPDTTDKALLEIN 250
251 NGGHSCANGGGSNGGLLGKYGVSWMKRFIDNDTRYSQFLCGPNHAANSAI 300
301 SEYRETCDY 309
10 20 30 40 50
Strand
Helix
Coil
Disordered
Disordered, protein binding
Putative Domain Boundary
Membrane Interaction
Transmembrane Helix
Extracellular
Re-entrant Helix
Cytoplasmic
Signal Peptide

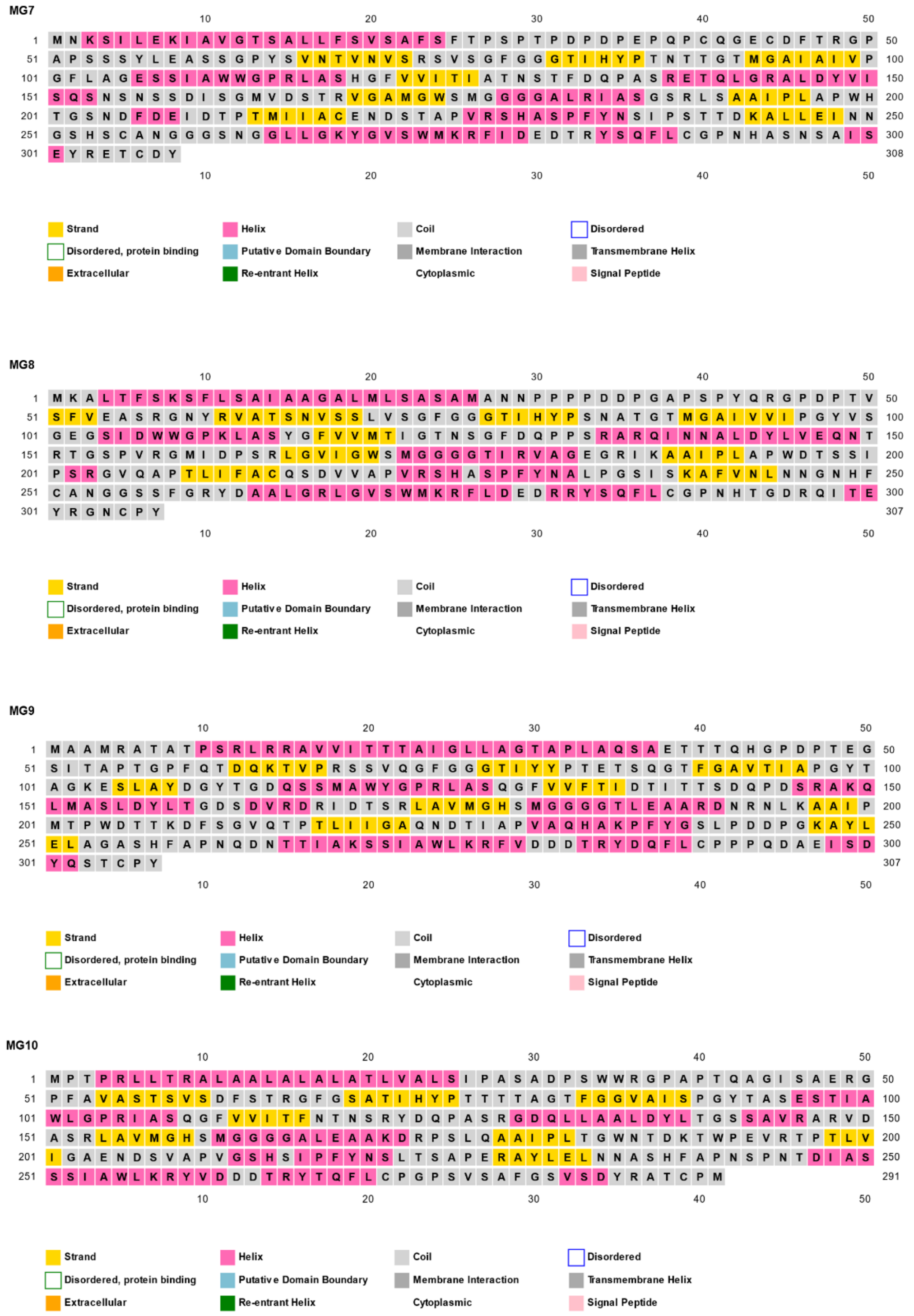

Figure S3. Secondary structure prediction of MG1-MG10 by PSIPRED[1]. Similar to *Is*PETase, MG1-MG10 enzymes are predicted to contain a canonical alpha/beta hydrolase fold comprising eight beta-strands connected by helices.

MG1

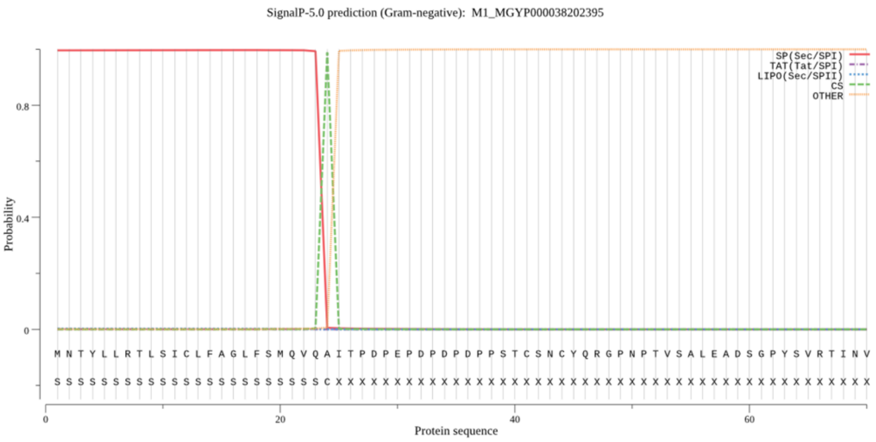
SignalP-5.0 prediction (Gram-negative): M1_MGYP000038202395
SP(Sec/SPI)
TAT(Tat/SPI)
LIPO(Sec/SPII)
CS
OTHER
Probability
0.8
0.4
0
M N T Y L L R T L S I C L F A G L F S M Q V Q A I T P D P E P D P D P D P P S T C S N C Y Q R G P N P T V S A L E A D S G P Y S V R T I N V
S S S S S S S S S S S S S S S S S S S S S S S C X X X X X X X X X X X X X X X X X X X X X X X X X X X X X X X X X X X X X X X X X X X X X X
0
20
40
60
Protein sequence

MG2

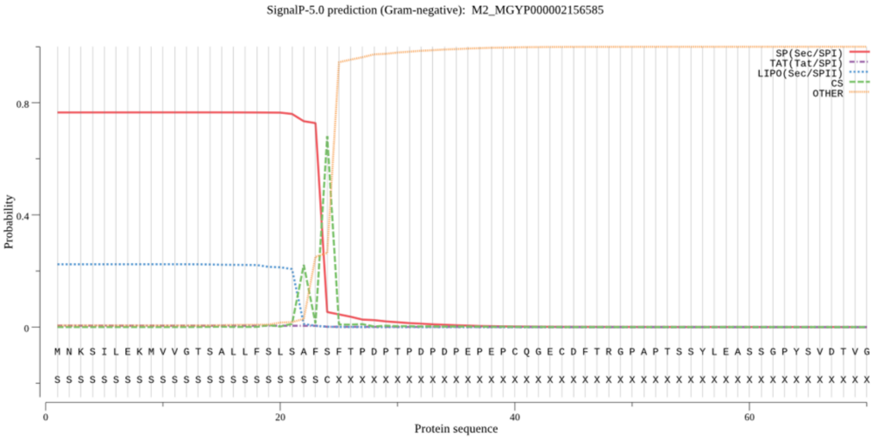
SignalP-5.0 prediction (Gram-negative): M2_MGYP000002156585
SP(Sec/SPI)
TAT(Tat/SPI)
LIPO(Sec/SPII)
CS
OTHER
Probability
0.8
0.4
0
M N K S I L E K M V V G T S A L L F S L S A F S F T P D P T P D P D P E P E P C Q G E C D F T R G P A P T S S Y L E A S S G P Y S V D T V G
S S S S S S S S S S S S S S S S S S S S S S S C X X X X X X X X X X X X X X X X X X X X X X X X X X X X X X X X X X X X X X X X X X X X X X
0
20
40
60
Protein sequence

MG3

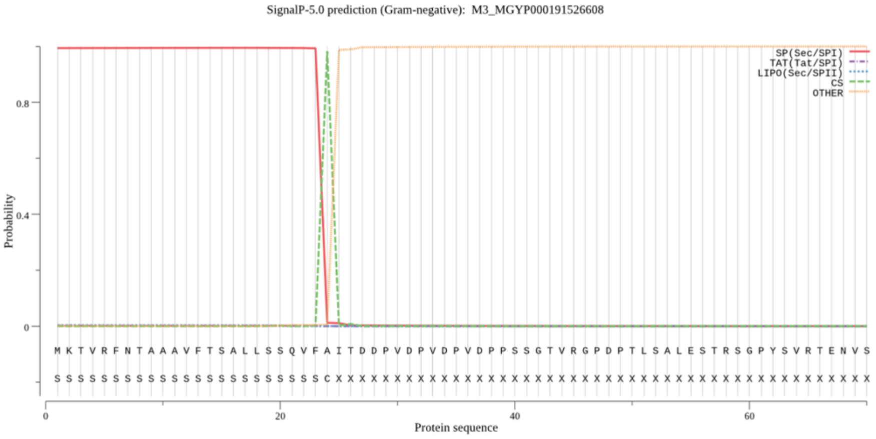
SignalP-5.0 prediction (Gram-negative): M3_MGYP000191526608
SP(Sec/SPI)
TAT(Tat/SPI)
LIPO(Sec/SPII)
CS
OTHER
Probability
0.8
0.4
0
M K T V R F N T A A A V F T S A L L S S Q V F A I T D D P V D P V D P V D P P S S G T V R G P D P T L S A L E S T R S G P Y S V R T E N V S
S S S S S S S S S S S S S S S S S S S S S S S C X X X X X X X X X X X X X X X X X X X X X X X X X X X X X X X X X X X X X X X X X X X X X X
0
20
40
60
Protein sequence

MG4

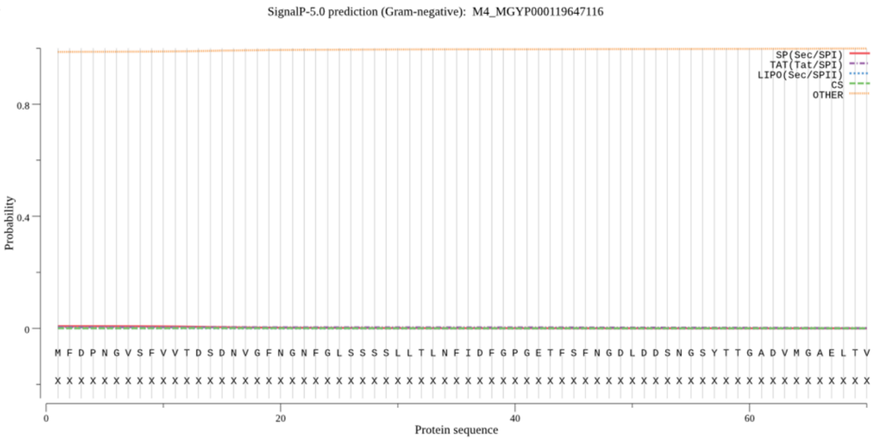
SignalP-5.0 prediction (Gram-negative): M4_MGYP000119647116
SP(Sec/SPI)
TAT(Tat/SPI)
LIPO(Sec/SPII)
CS
OTHER
Probability
0
0.4
0.8
M F D P N G V S F V V T D S D N V G F N G N F G L S S S S L L T L N F I D F G P G E T F S F N G D L D D S N G S Y T T G A D V M G A E L T V
X X X X X X X X X X X X X X X X X X X X X X X X X X X X X X X X X X X X X X X X X X X X X X X X X X X X X X X X X X X X X X X X X X X X X X
0
20
40
60
Protein sequence

MG5

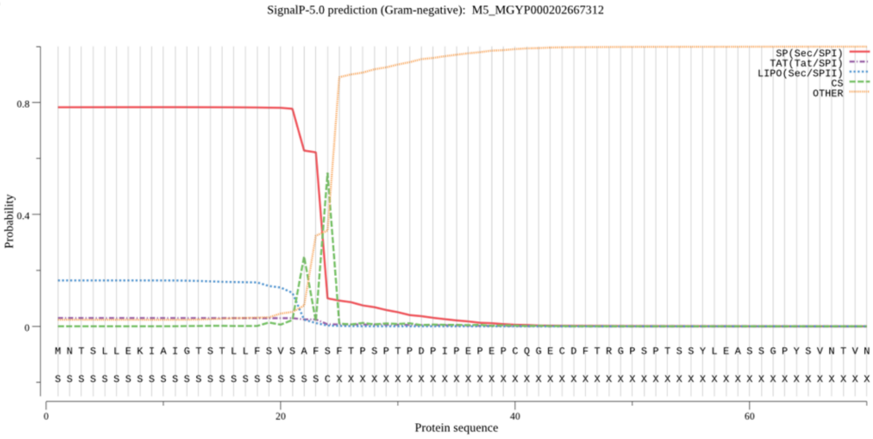
SignalP-5.0 prediction (Gram-negative): M5_MGYP000202667312
SP(Sec/SPI)
TAT(Tat/SPI)
LIPO(Sec/SPII)
CS
OTHER
Probability
0
0.4
0.8
M N T S L L E K I A I G T S T L L F S V S A F S F T P S P T P D P I P E P E P C Q G E C D F T R G P S P T S S Y L E A S S G P Y S V N T V N
S S S S S S S S S S S S S S S S S S S S S C X X X X X X X X X X X X X X X X X X X X X X X X X X X X X X X X X X X X X X X X X X X X X X X X
0
20
40
60
Protein sequence

MG6

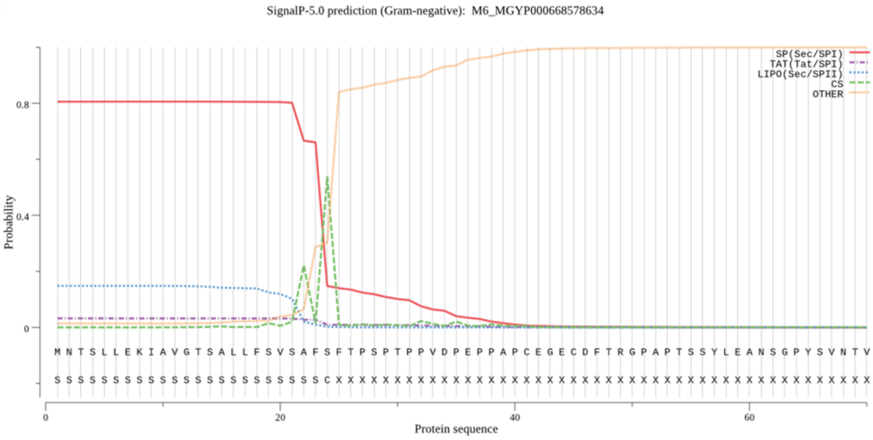
SignalP-5.0 prediction (Gram-negative): M6_MGYP000668578634
SP(Sec/SPI)
TAT(Tat/SPI)
LIPO(Sec/SPII)
CS
OTHER
Probability
0
0.4
0.8
M N T S L L E K I A V G T S A L L F S V S A F S F T P S P T P P V D P E P P A P C E G E C D F T R G P A P T S S Y L E A N S G P Y S V N T V
S S S S S S S S S S S S S S S S S S S S S C X X X X X X X X X X X X X X X X X X X X X X X X X X X X X X X X X X X X X X X X X X X X X X X X
0
20
40
60
Protein sequence

MG7

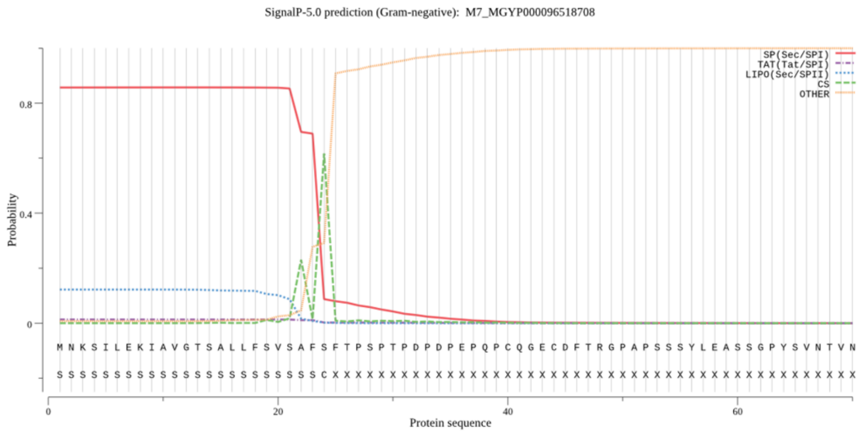

MG8

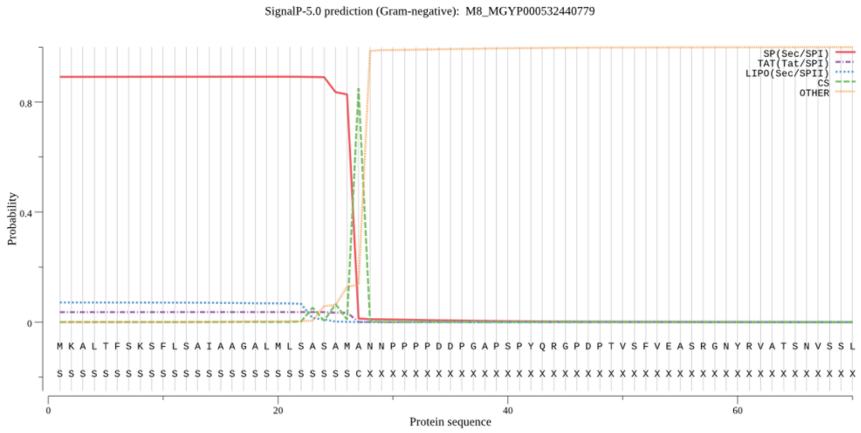

MG9

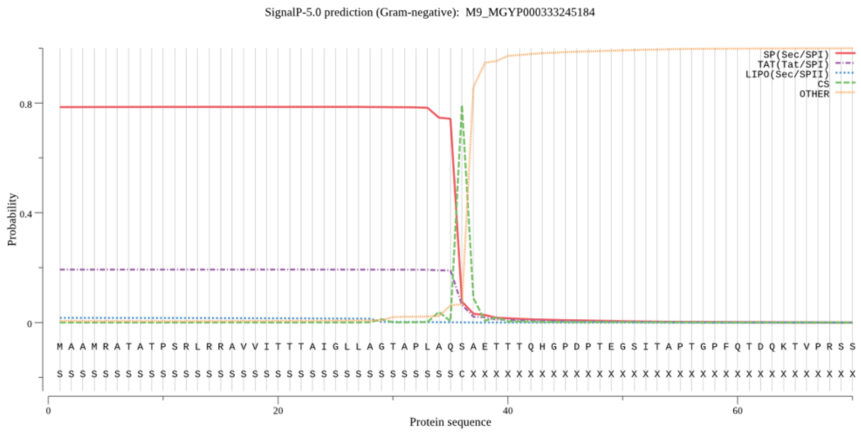

MG10

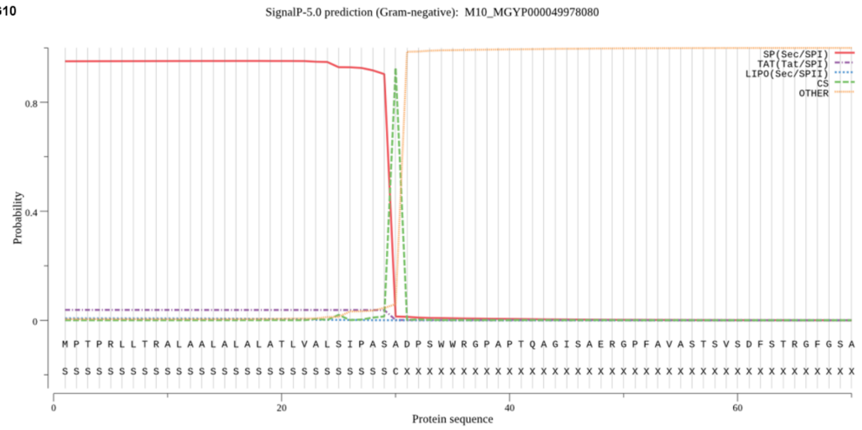

Figure S4. The likelihood of presence of a signal peptide in MG1-MG10 sequences, as predicted by SignalP5.0[2]. Aside from MG4, all other putative PET hydrolases are predicted to contain Sec signal peptides cleavable by SPase I (Sec/SPI).

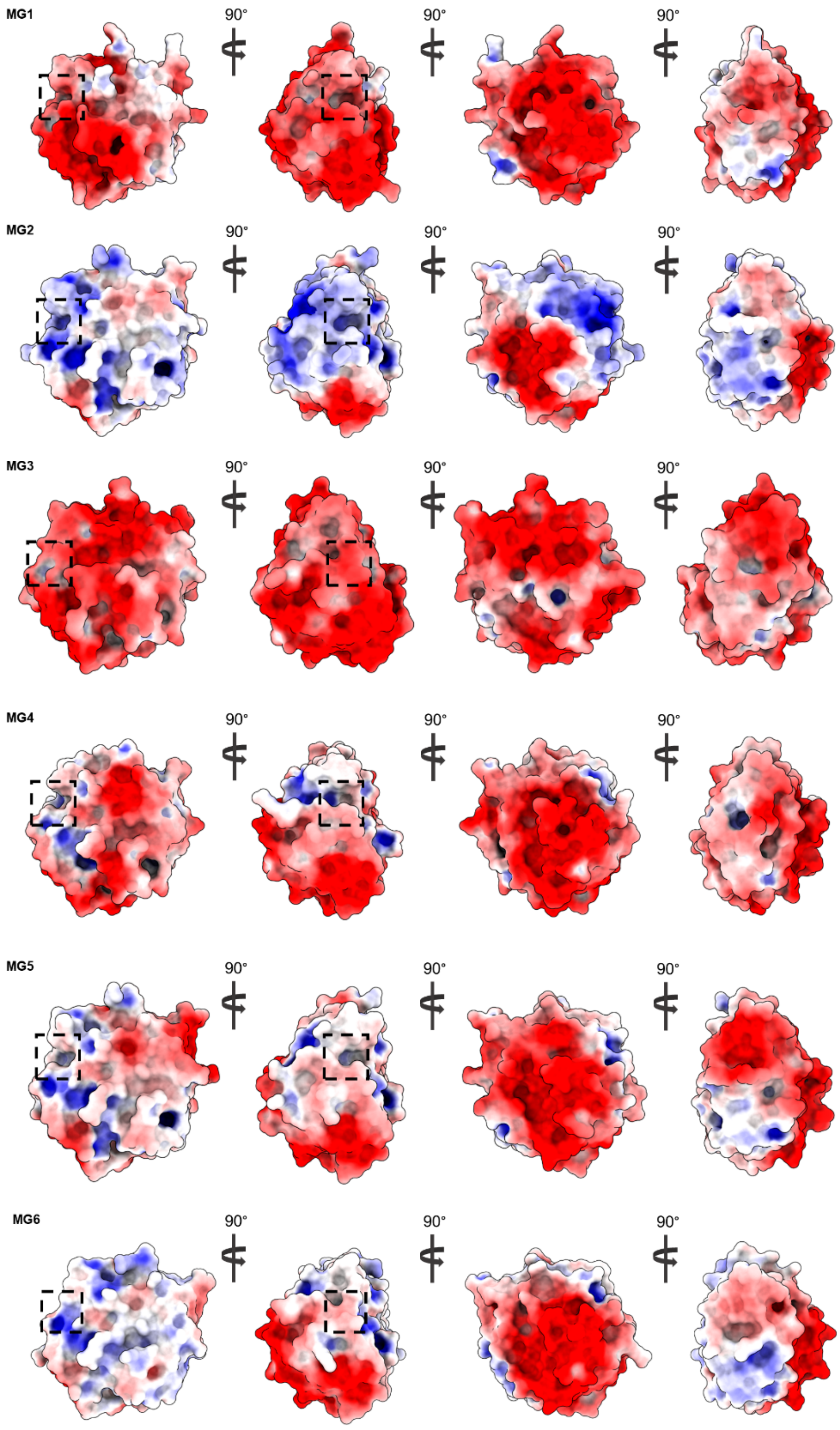
MG1
90°
90°
90°
MG2
90°
90°
90°
MG3
90°
90°
90°
MG4
90°
90°
90°
MG5
90°
90°
90°
MG6
90°
90°
90°

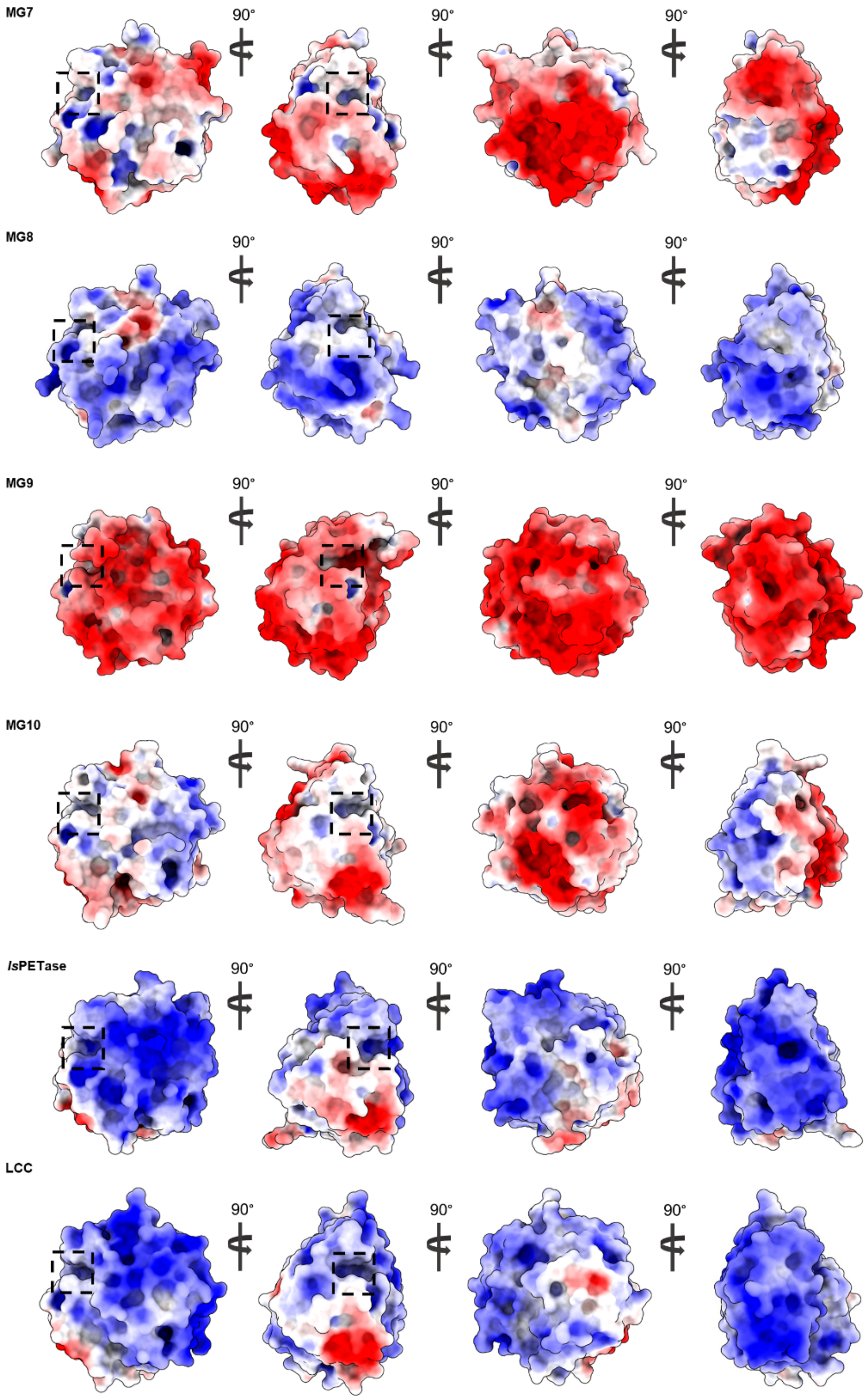

Figure S5. Electrostatic surface potential of MG1-MG10, *Ideonella sakaiensis* (*Is*PETase) and leaf-branch compost (LCC) at pH 7.0. The catalytic pocket is indicated by a dashed rectangle. The distribution of surface potential is coded in red, white, and blue regions, representing areas of negative, neutral, and positive potential respectively (scale of -5.0 to +5.0 k_BT/e_c).

*Is*PETase	S160	H237	D206	Y87	Q119	M161	W185	T88	A89	W159	S238	N241	S242	G243	N244	S245	N246	Q247	C203	C239	Type
MG1	S	H	D	Y	Q	M	Y	V	S	W	F	N	S	G	Y	P	D	E	C	C	II
MG2	S	H	D	F	Q	M	W	L	A	W	S	N	G	G	G	S	N	G	C	C	II
MG3	S	H	D	Y	Q	M	W	V	S	W	Y	N	S	G	Y	S	D	E	C	C	II
MG4	S	H	D	F	Q	M	F	L	A	W	S	N	G	G	S	N	-	R	C	C	II
MG5	S	H	D	F	Q	M	W	F	A	W	S	N	G	G	G	S	N	G	C	C	II
MG6	S	H	D	F	Q	M	W	L	A	W	S	N	G	G	G	S	N	G	C	C	II
MG7	S	H	D	F	Q	M	W	L	A	W	S	N	G	G	G	S	N	G	C	C	II
MG8	S	H	D	Y	Q	M	W	V	S	W	F	N	G	G	S	S	Y	D	C	C	II
MG9	S	H	D	Y	Q	M	W	T	A	H	F	N	Q	D	N	T	-	-	A	A	I
MG10	S	H	D	Y	Q	M	W	T	A	H	F	N	S	P	N	T	-	-	A	A	I

Figure S6. Amino acid sequence alignment of important residues in putative PET-degrading enzymes MG1-MG10, and their Type I vs II classification as proposed by Joo *et al*[3]. *Is*PETase is used as the reference sequence for the alignment. Type II PET hydrolases, which include *Is*PETase, has an additional disulfide bond near their active site. The additional disulfide bond is known to increase both the activity and stability of these enzymes[3].

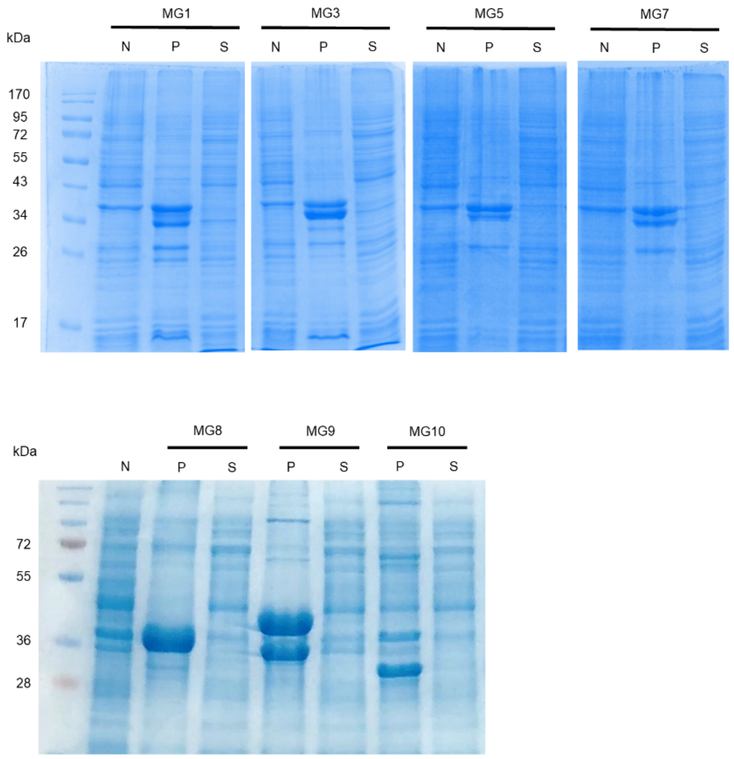

Figure S7. Partitioning of MG enzymes into inclusion bodies upon expression in BL21(DE3). N = non-induced cells; P = pellet/inclusion bodies; S = supernatant. Expected protein size: MG1, MG3, MG5, MG7 and MG10, 35 kD; MG8, 37 kD; MG9, 39 kD.

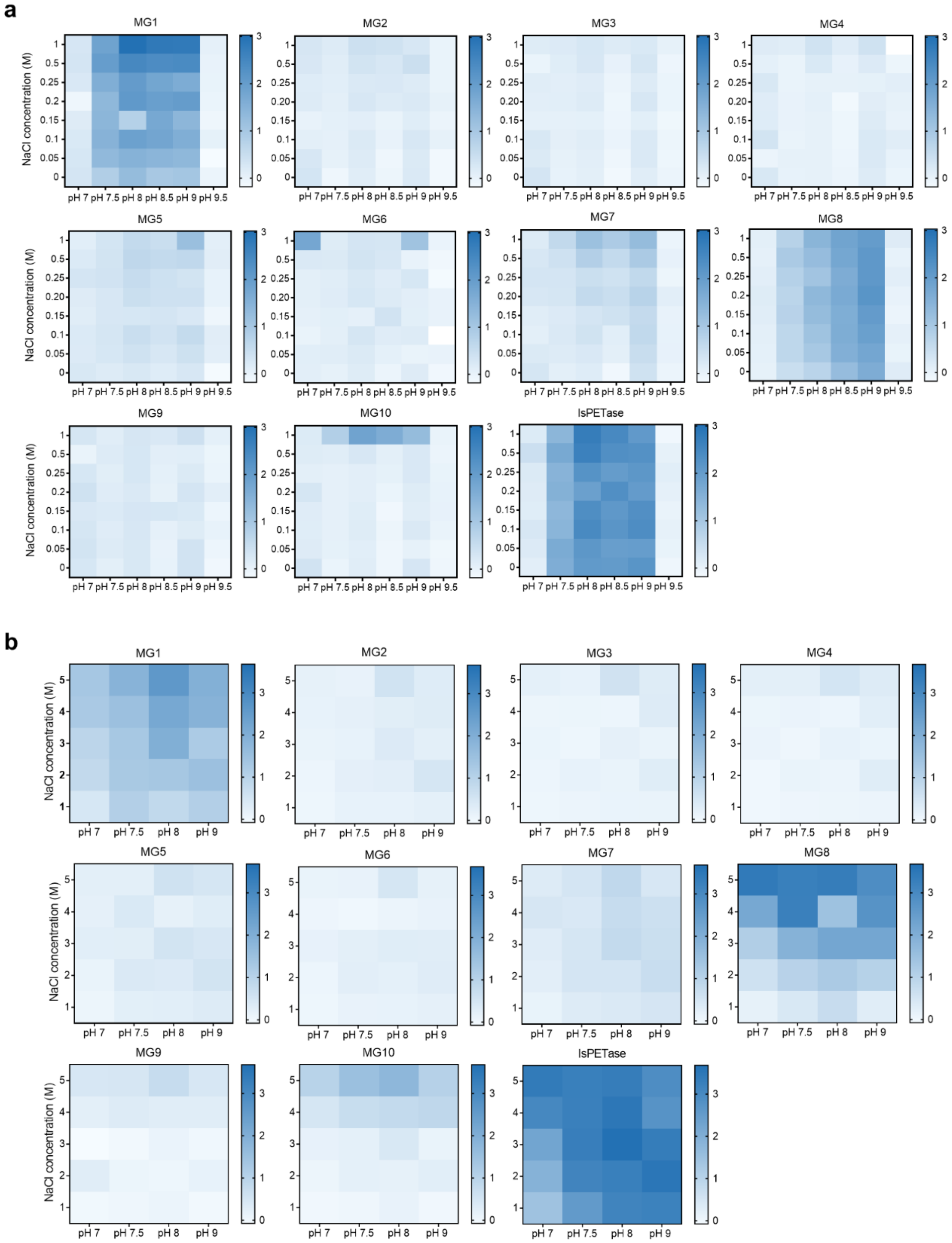

Figure S8. Measuring relative esterase activity of putative PET hydrolases using *p*-nitrophenyl acetate (pNpA). a) Heat maps showing the activity of 300 nM of one the ten putative hydrolases (MG1-MG10) in hydrolyzing 5 mM pNpA under different pHs (7-9.5) and NaCl concentrations (0-1 M). b) Measuring relative esterase activity of putative PET hydrolases using *p*-nitrophenyl acetate (pNpA), at higher NaCl concentration ranges. End-point mean absorbance values at 405 nm after 12-min reactions from three replicates are shown.

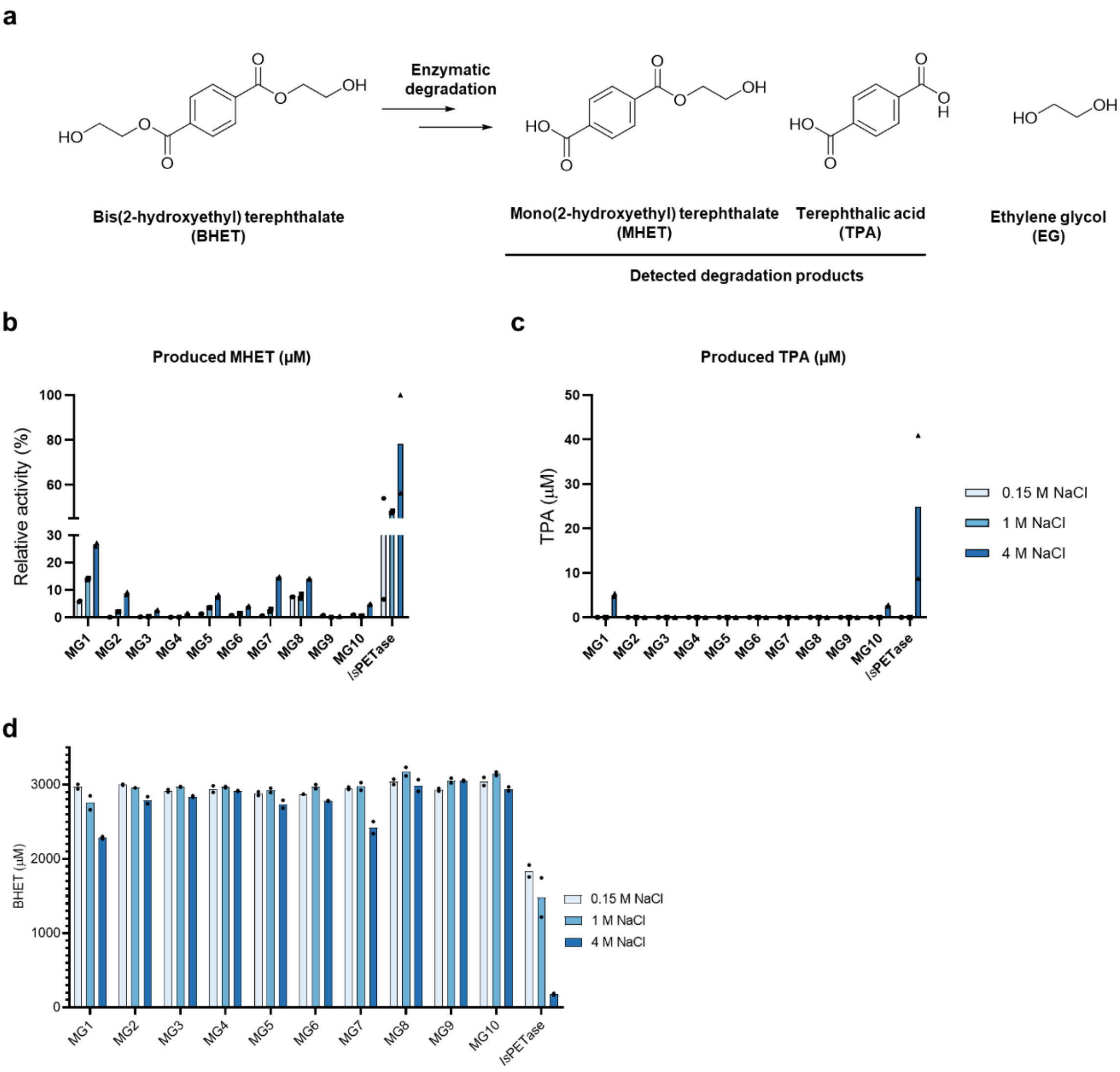

Figure S9. Measuring hydrolysis of a small-molecule PET surrogate bis(2-hydroxyethyl) terephthalate (BHET) by putative PET hydrolases. a) BHET can be hydrolyzed to generate ethylene glycol (EG) and mono(2-hydroxyethyl) terephthalate (MHET), the latter of which is further hydrolyzed to generate EG and terephthalic acid (TPA). The amount of MHET and TPA produced was monitored by HPLC. b) relative activity of 500 nM enzyme (MG1, MG7, MG8, or MG10) in hydrolyzing 3 mM BHET to produce MHET. Hydrolysis was allowed to proceed for 30 min at 37 °C, under three different NaCl concentrations (0.15, 1, and 4 M) before reactions were quenched with 5 mM phenylmethylsulfonyl fluoride (PMSF) and analyzed by HPLC. *Is*PETase activity to generate MHET under the same condition acts as a reference point. c) TPA generated from the same reactions as in b). d) consumption of BHET from the same reactions as in b). Each bar graph represents mean values from duplicate experiments.

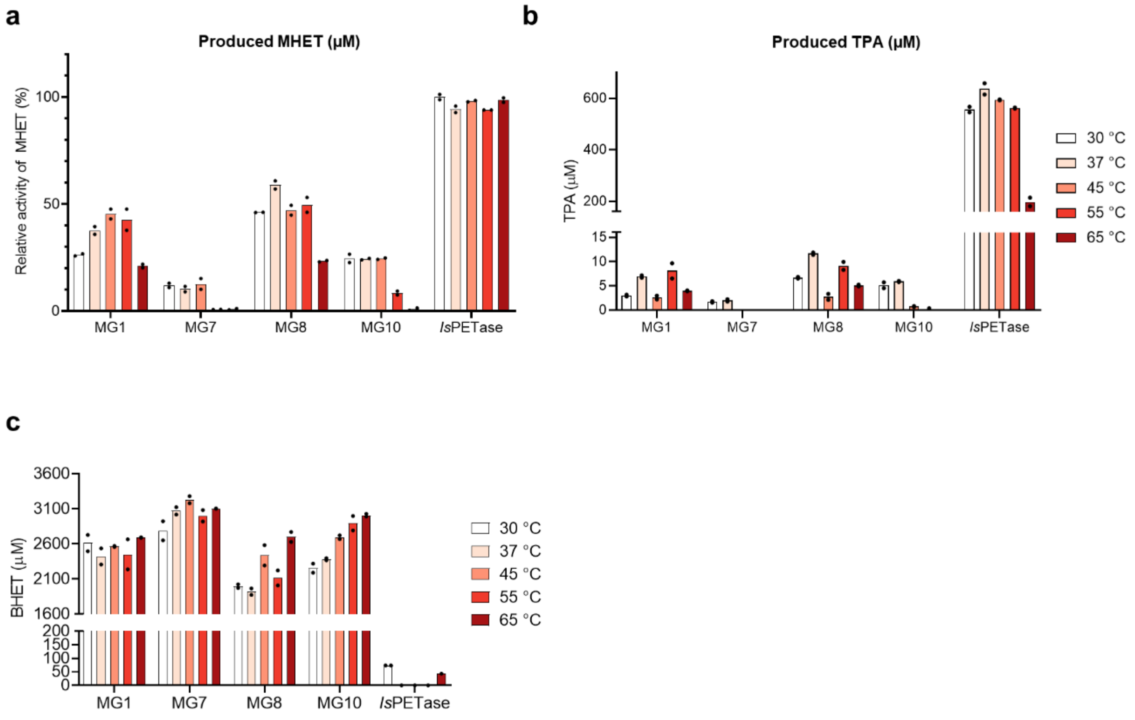

Figure S10. Hydrolysis of BHET by putative PET hydrolases under different reaction temperatures. a) relative activity of 500 nM enzyme (MG1, MG7, MG8, or MG10) in hydrolyzing 1.5 mM BHET to produce MHET. Hydrolysis was allowed to proceed for 30 min at different temperatures (30, 37, 45, 55, or 65 °C), under 4 M NaCl concentration before reactions were quenched with 5 mM PMSF and analyzed by HPLC. b) TPA generated from the same reactions as in a). c) consumption of BHET from the same reactions as in a). Each bar graph represents mean values from duplicate experiments.

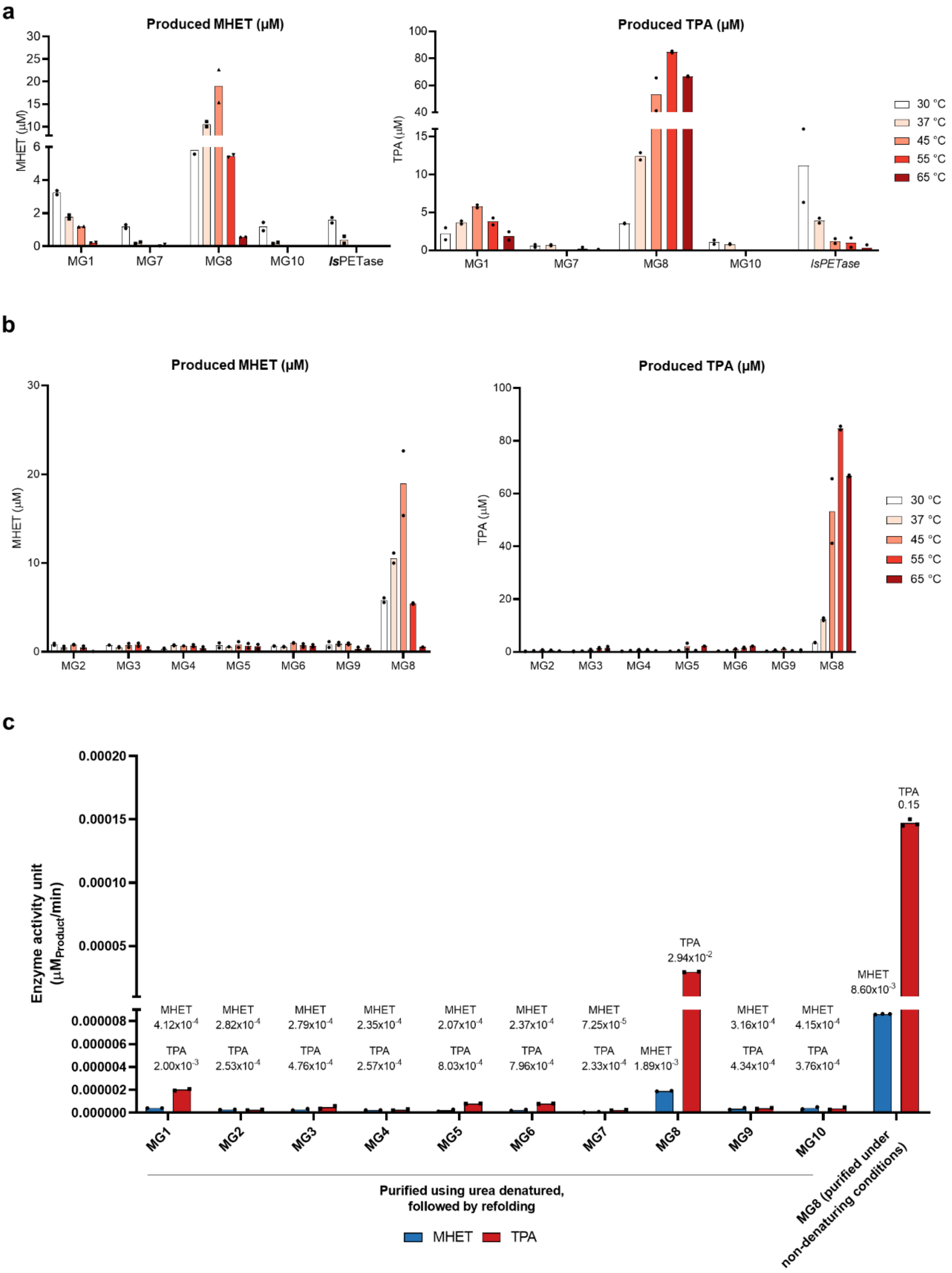

Figure S11. a) Relative activity of 500 nM enzyme (MG1, MG7, MG8, or MG10) in hydrolyzing 20 mg PET powder to generate MHET and TPA (same underlying data as Figure 2b). Hydrolysis was allowed to proceed in buffer containing 4 M NaCl for 48 h at different temperatures (30, 37, 45, 55, or 65 °C), before reactions were quenched with 5 mM phenylmethylsulfonyl fluoride (PMSF) and analyzed by HPLC. Each bar graph represents mean values from duplicate experiments. b) Degradation of PET powder by other less active putative PET hydrolases (MG2, MG3, MG4, MG5, MG6, MG9). Relative activity of 500 nM enzyme in hydrolyzing 20 mg PET powder. Hydrolysis was allowed to proceed in buffer containing 4 M NaCl for 48 h at different temperatures (30, 37, 45, 55, or 65 °C), before reactions were quenched with 5 mM phenylmethylsulfonyl fluoride (PMSF) and analyzed by HPLC. Each bar graph represents mean values from duplicate experiments. c) Enzyme activity unit ($\mu M_{Product}$ min^{-1}) fort the MG enzymes at their respective optimal temperatures: 30 °C (MG10), 37°C (MG7), 45 °C (MG1, MG2, MG4, and MG9), 55 °C (MG3, and MG8), and 65 °C (MG5, and MG6). The hydrolysis reactions were performed with 20 mg PET powder in 4 M NaCl for 48 h. Each bar graph represents mean values from duplicate experiments.

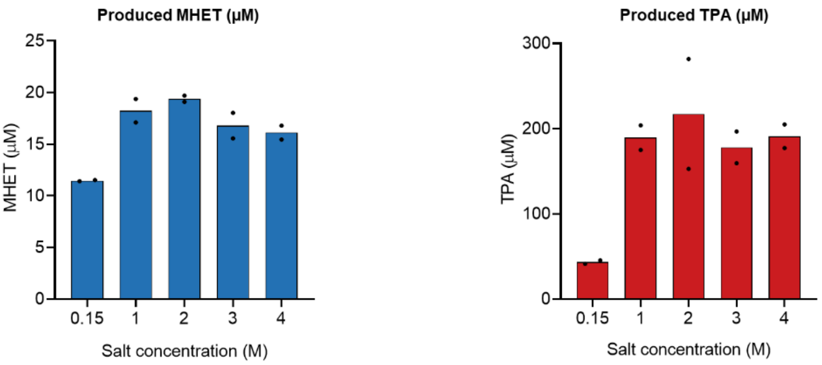

Figure S12. High activity of MG8 in hydrolyzing PET powder to produce MHET and TPA at 1-4 M NaCl concentrations. Each bar graph shows the mean amount of products generated in duplicate experiments.

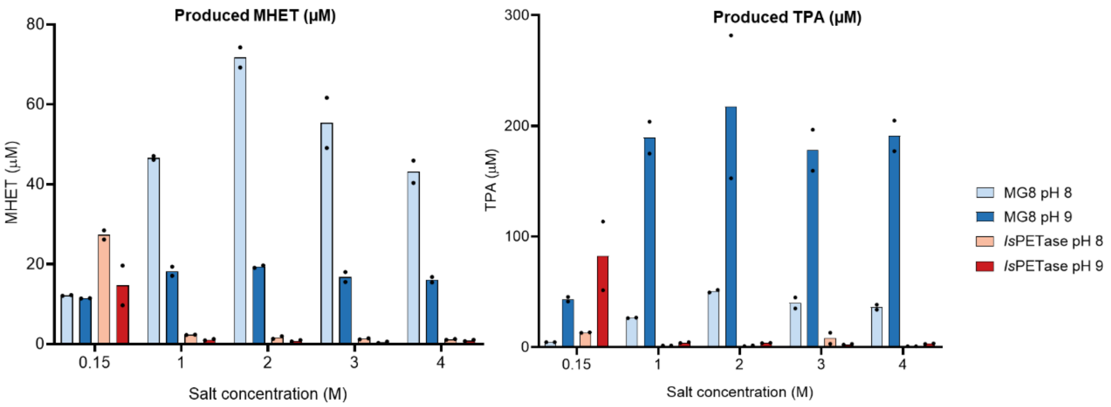

Figure S13. Comparing activity of MG8 and *Is*PETase at 55 °C, at reaction pH of 8 or 9. Each bar graph represents mean values from duplicate experiments.

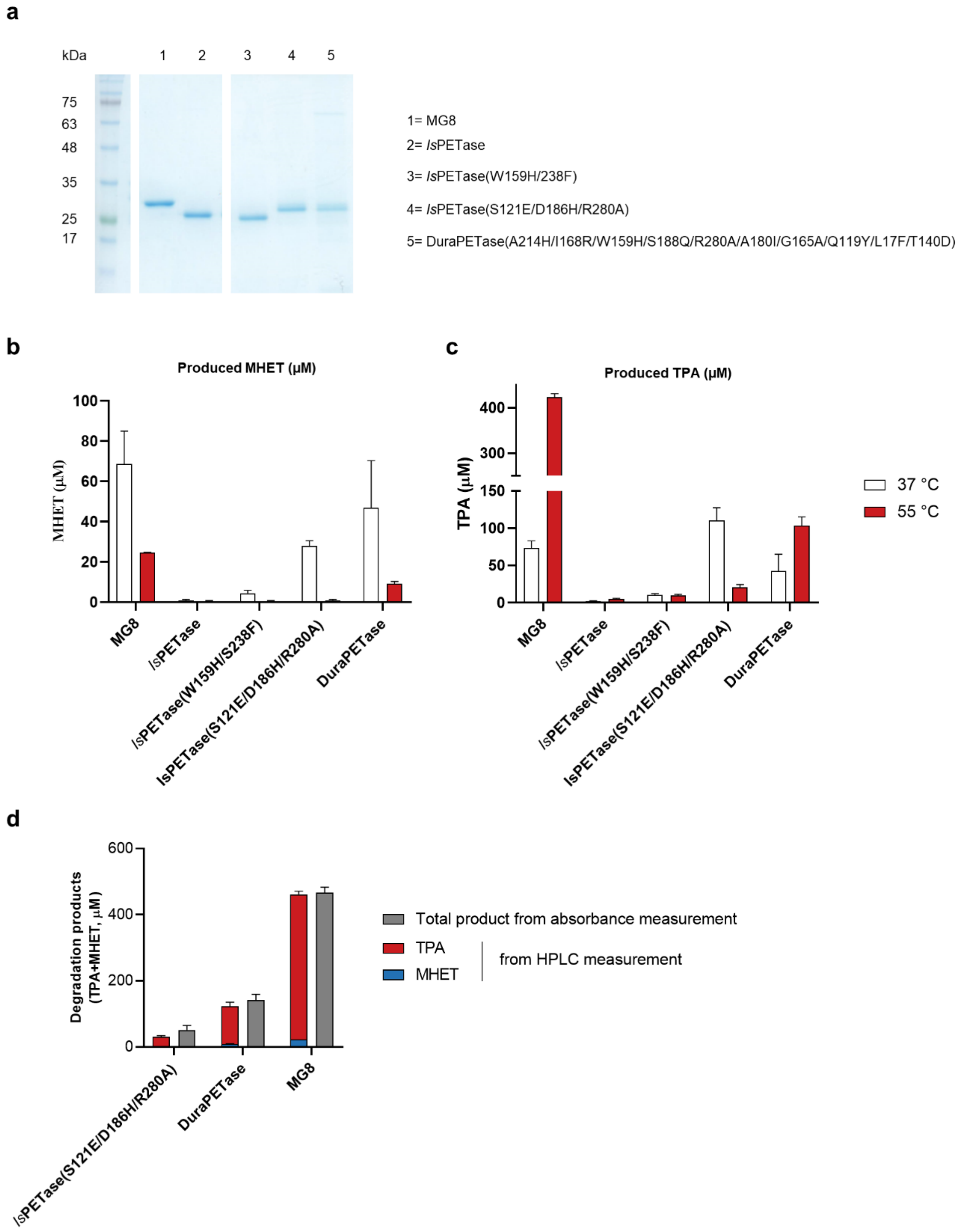

Figure S14. Comparison of enzyme activity of MG8 and *Is*PETase variants (same underlying data as Figure 3a). a) Protein purity of MG8 and *Is*PETase variants. All enzymes were expressed and purified during the same period to minimize bias due to enzyme inactivation during storage. ThermoPETase (*Is*PETase with S121E/D186H/R280A mutations) and DuraPETase (*Is*PETase with A214H/I168R/W159H/S188Q/R280A/A180I/G165A/Q119Y/L17F/T140D mutations) had an additional G11 tag (for split-GFP reconstitution), resulting in the apparent size shift on SDS-PAGE. b, c) Relative activity of 500 nM enzyme in hydrolyzing 10 mg PET powder. Hydrolysis was allowed to proceed in buffer containing 50 mM Glycine-NaOH, pH 9.0 and 4 M NaCl for 48 h at either 37 or 55 °C, before reactions were quenched with 5 mM phenylmethylsulfonyl fluoride (PMSF) and analyzed by HPLC. MHET, and TPA, generated from the reactions are shown. d) Validation of the absorbance method for analysis of enzymatic degradation products (right bar for each enzyme) via comparison to product measurements from HPLC (left bar for each enzyme). Absorbance measurements at 260 nm (A_{260}) were performed on the same reactions as in b), prior to PMSF quenching. Total amount in of degradation products generated (primarily TPA, with <10% MHET) was calculated from A_{260} based on TPA extinction coefficient. Each bar graph represents mean values from triplicate experiments. Error bars, +/- S.D.

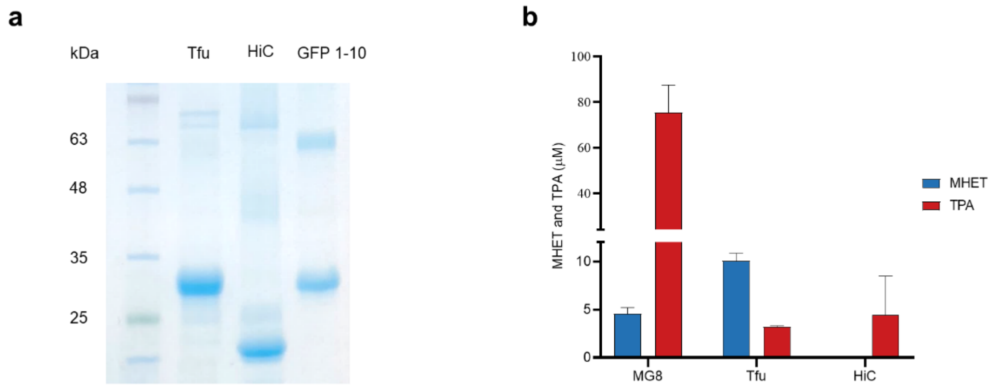

Figure S15. Comparison of MG8 to other known PET hydrolases, *Thermobifida fusca* cutinase (Tfu) and *Humicola insolens* cutinase (HiC). a) Protein purity of Tfu, HiC, and GFP1-10 (used for Figure 4). b) Generated MHET and TPA from PET powder degradation assay performed in 4M NaCl and at 55 °C, 60 °C (Tfu), and 80 °C (HiC) for 48 h are shown. Each bar graph represents mean values from triplicate experiments. Error bars, +/- S.D.

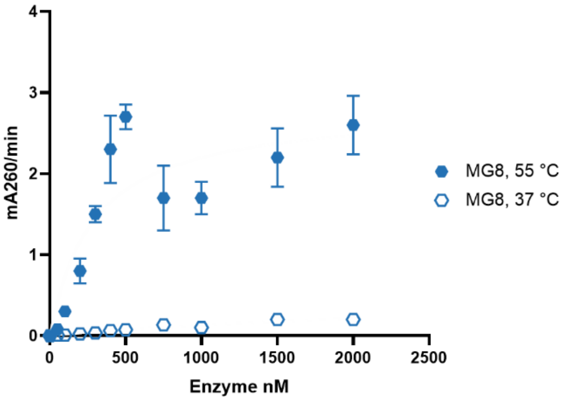

Figure S16. Hydrolysis rates of PET powder as a function of enzyme concentration. PET hydrolysis reactions were performed with 15 mg PET powder and 0-2 uM MG8 at 37 °C or 55 °C. Reaction rates were calculated in terms of absorbance at 260 nm (A_{260}, which reflects generated TPA and MHET, the primary soluble hydrolysis products) per minute, at a given enzyme concentration (n=3 per concentration). Error bars, ± SD.

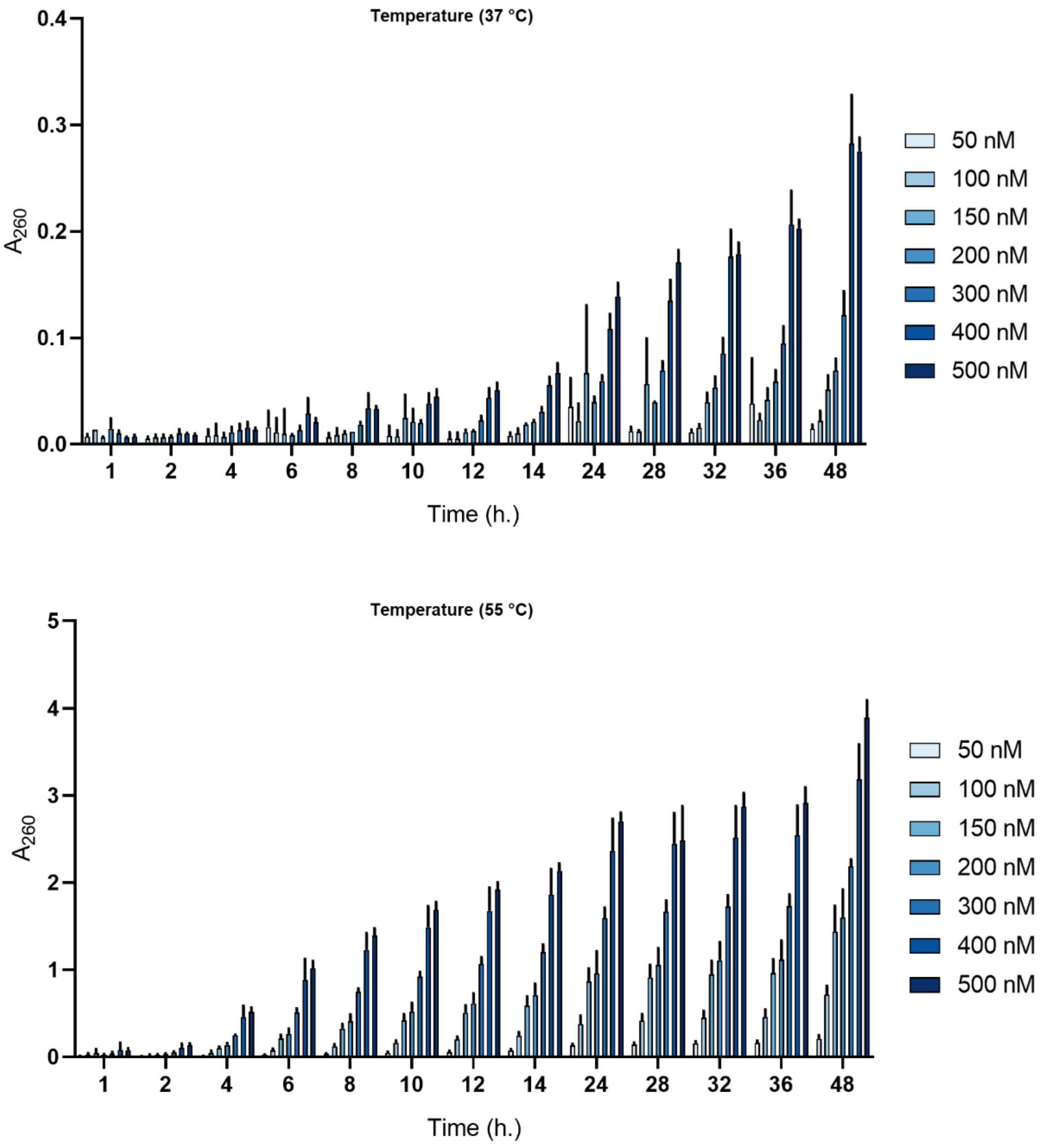

Figure S17. Time-coursed absorbance measurements of enzymatic degradation products from MG8. This data was used to calculated the reaction rates shown in Figure 3b. PET hydrolysis reactions were performed with 15 mg PET powder and 0-500 nM MG8 at 37 °C (top) or 55 °C (bottom), in buffer containing 50 mM Glycine-NaOH, pH 9.0, 4 M NaCl. A_{260} was measured every two hours. Each bar graph represents mean values from triplicate experiments. Error bars, +/- S.D.

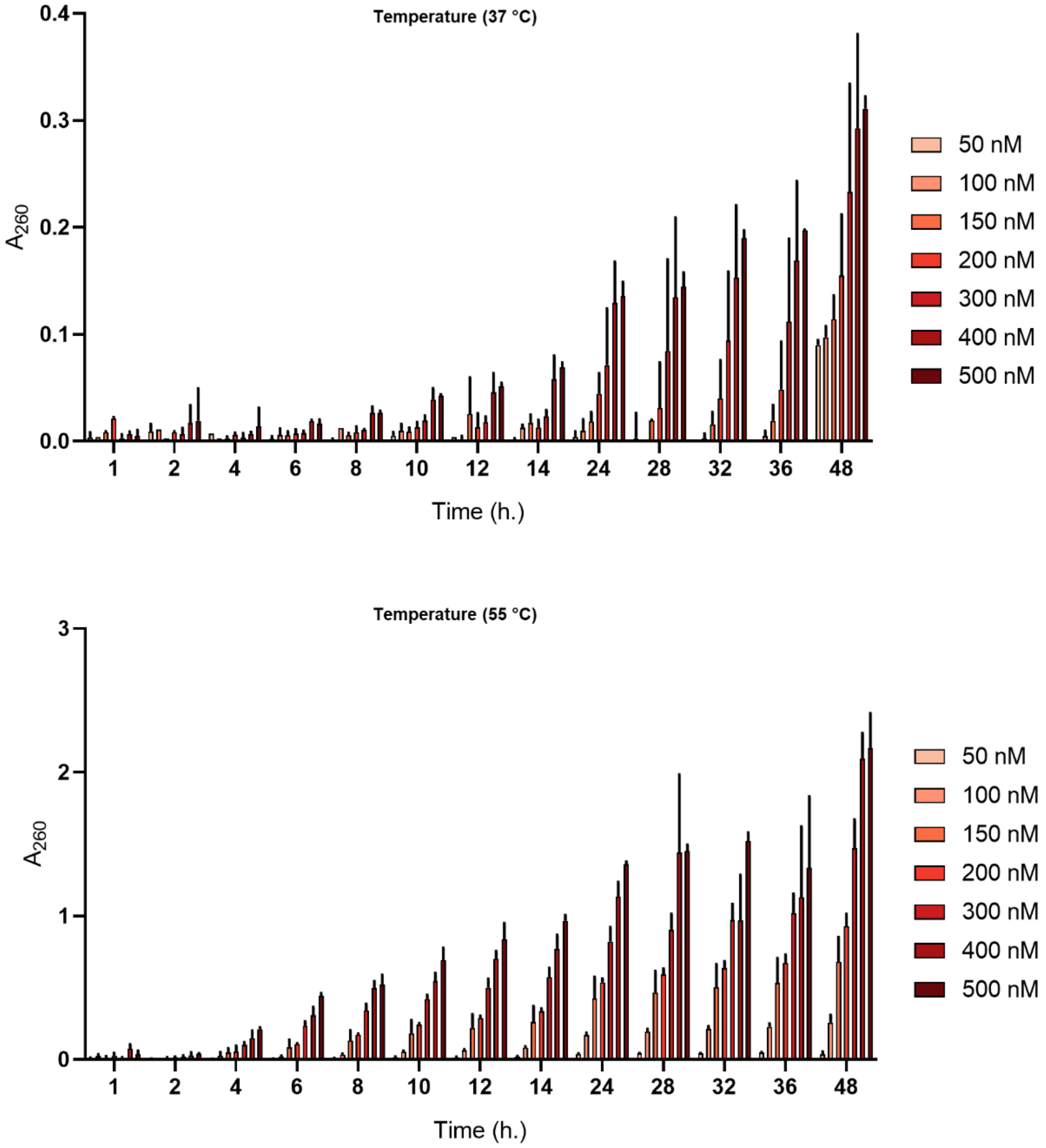

Figure S18. Time-coursed absorbance measurements of enzymatic degradation products from DuraPETase. This data was used to calculated the reaction rates shown in Figure 3b. PET hydrolysis reactions were performed with 15 mg PET powder and 0-500 nM DuraPETase at 37 °C (top) or 55 °C (bottom), in buffer containing 50 mM Glycine-NaOH, pH 9.0, 4 M NaCl. A_{260} was measured every two hours. Each bar graph represents mean values from triplicate experiments. Error bars, +/- S.D.

a

Sample	Temperature (°C)	Crystallinity (%) Rep. 1	Rep 2	Average
PET powder	-	31.23	-	-
PET powder	55	24.39	28.66	26.53
PET powder + MG8	55	27.81	36.96	32.39

b

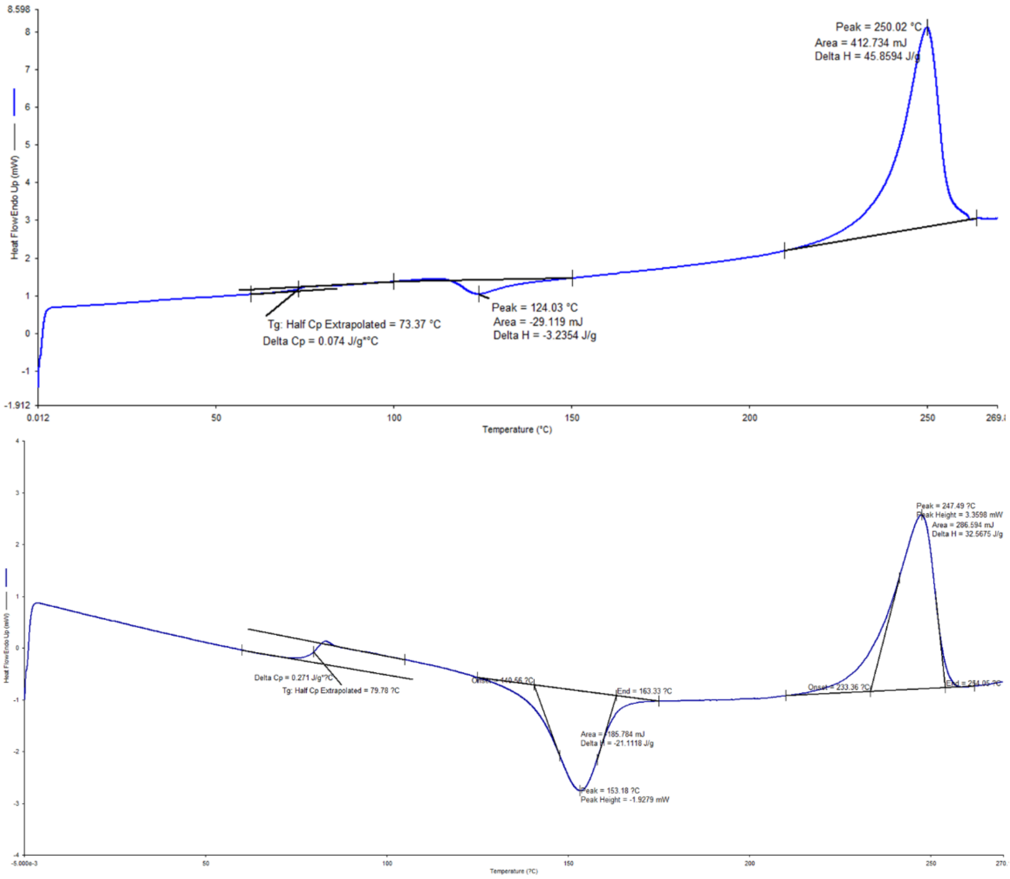

Figure S19. Differential scanning calorimetry characterization of PET powder. a) Percent crystallinity of PET powder (top); PET powder incubated in 4 M NaCl, pH 9.0 at 55 °C for 48 h, but without MG8 enzyme (middle); and PET powder incubated with 500 nM MG8 in 4 M NaCl, pH 9.0 at 55 °C for 48 h (bottom). b) Representative plots from the first heating-cooling cycle (top) and the second heating-cooling cycle (bottom) are shown.

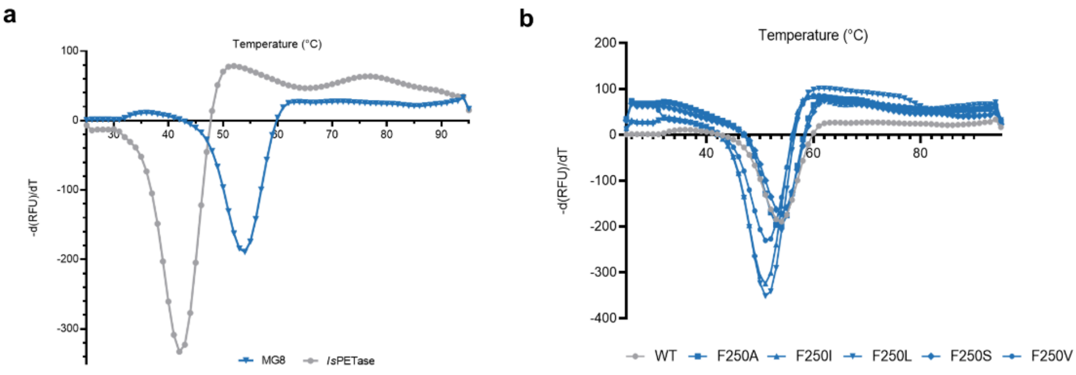

Figure S20. Comparison of enzyme thermal stability. a) Thermal stability of MG8 and *Is*PETase at 0.15 M NaCl concentrations. b) Thermal stability of MG8-F250 mutants.

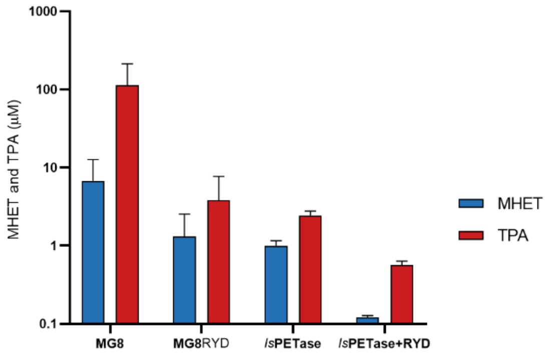

Figure S21. Effects of deletion of the extended RYD loop from MG8 (MG8(-RYD)) and grafting of the RYD loop onto *Is*PETase (*Is*PETase+RYD) on enzyme activity. Generated MHET and TPA from PET powder degradation assay performed in 4M NaCl and at 55 °C for 48 h are shown. Bar graphs show mean amount of products generated in triplicate experiments. Error bars, +/- S.D.

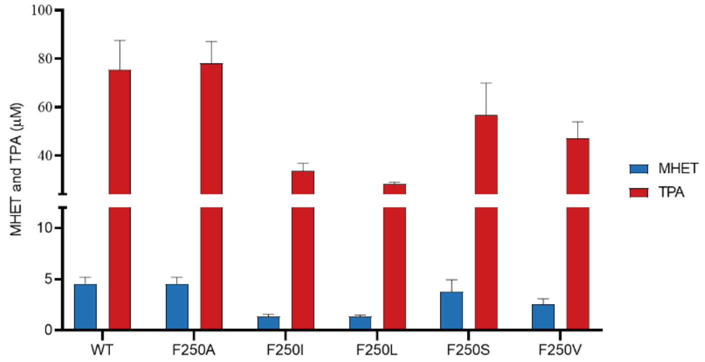

Figure S22. Generation of MHET and TPA from hydrolysis of BHET and PET powder with MG8-F250 mutants. Bar graphs show mean amount of products generated in triplicate experiments. Error bars, +/- S.D.

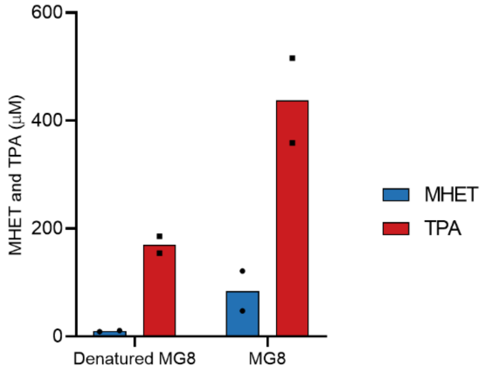

Figure S23. Comparing activity of MG8 purified under denaturing vs non-denaturing conditions. Generated MHET and TPA from PET powder degradation assay performed in 4 M NaCl and at 55 °C for 48 h are shown. Each bar graph represents mean values from duplicate experiments.

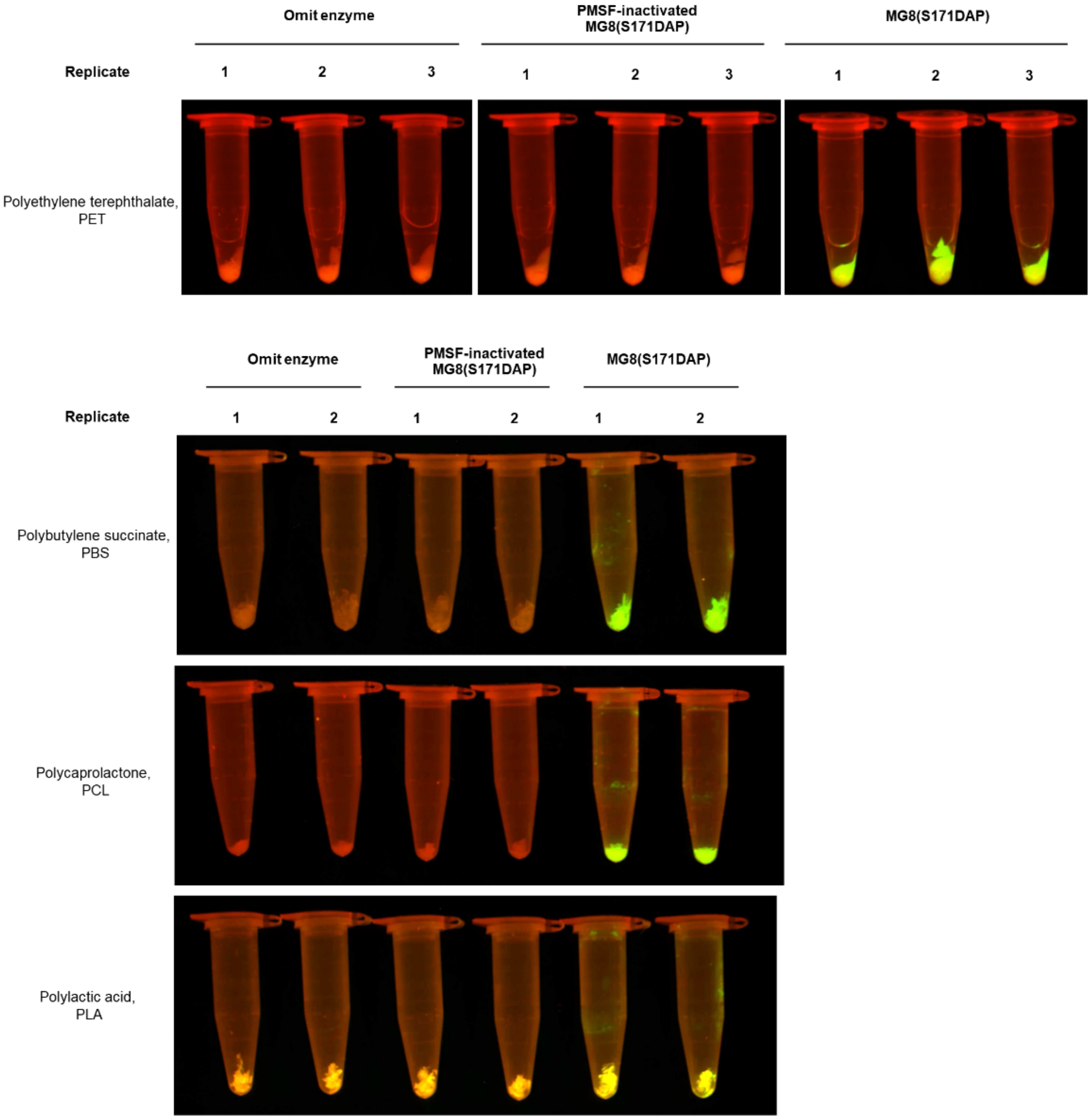

Figure S24. DAP incorporation in MG8 enables its stable entrapment to PET plastic (same data as Figure 5b). Results of PET from triplicate experiments are shown. Results of PBS, PCL and PLA from duplicate experiments are shown.

Table S1. Summary of sequence-level properties of MG1-MG10.

Published ID	Sequence length	Percent Identity with *Is*PETase	Percent similarity with *Is*PETase	Putative host	pI	Start codon	Signal peptide cleavage site	PETase classification type	Number of putative lipase chaperones	PDB ID of Structural homology template (percent similarity calculated by SWISS-MODEL)
MG1	307	48.2	61.7	Gammaproteobacteria: Order Oceanospirillales	4.62	ATG	24/25	IIa	1	6scd (49 %)
MG2	308	51.8	67.2	Gammaproteobacteria: Order Oceanospirillales	5.22	ATG	24/25	IIa	2	6scd (49 %)
MG3	307	50.8	67.6	Gammaproteobacteria: Order Oceanospirillales: Ketobacter sp. MCCC 1A13808	4.58	ATG	24/25	IIa	2	7ec8 (50 %)
MG4	371	43.7	56.8	Gammaproteobacteria: Order Oceanospirillales/Pseudomonadales	4.25	GTG	N/A	IIa	N/A	7ecb (47 %)
MG5	308	51.7	66.7	Gammaproteobacteria: Order Alteromonadales/Enterobacterales	4.82	ATG	24/25	IIa	1	6scd (48 %)
MG6	309	50.6	65.5	Gammaproteobacteria: Order Oceanospirillales: Oleispira sp.	4.95	ATG	24/25	IIa	2	7ec8 (50 %)
MG7	308	50.8	64.8	Gammaproteobacteria: Order Oceanospirillales	4.96	ATG	24/25	IIa	N/A	6scd (49 %)
MG8	308	45.5	63.9	Gammaproteobacteria: Order Pseudomonadales: Pseudomonas sp.	9.23	ATG	27/28	IIa	1	6scd (54 %)
MG9	308	44.8	58.7	Actinobacteria: Order Pseudonocardiales: Saccharopolyspora sp.	4.87	ATG	36/37	I	N/A	7nei (50 %)
MG10	291	44.9	61.8	Actinobacteria: Order Geodermatophilales	6.41	GTG	30/31	I	N/A	3vis (47 %)

Table S2. MGnify attributes of MG1-MG10

Published ID	Target ID	Run ID	MGnify ID	Sample ID	Run/assembly accession	Analysis accession	Longitude	Latitude	Study description
MG1	MGYP000038202395	SRR3962293, ERR2094170, ERR2094164	MGYS00003217, MGYS00003351	SRS1589192, ERS1869125, ERS1869119	ERZ505234, ERZ650159, ERZ650162	MGYA00237656, MGYA00238072, MGYA00238077	-163.53, 121.39, 121.39	9.22, 37.52, 37.52	Marine microbial communities from the Deep Pacific Ocean - MP1788, water samples from conventional and recirculating aquaculture systems
MG2	MGYP000002156585	SRR5469033, SRR5469068, SRR5469034, SRR5469066	MGYS00003337, MGYS00003548, MGYS00003322, MGYS00003323	SRS2139711, SRS2139748, SRS2139712, SRS2139745	ERZ534229, ERZ511469, ERZ511465, ERZ511466	MGYA00237882, MGYA00248564, MGYA00237859, MGYA00237860	-72.4, -72.4, -72.4, -72.4	39.55, 39.54, 39.29, 39.29	Methane-oxidizing microbial communities from mesocosms in the Hudson Canyon - EN1E, EN10B, EN8C, EN8B Hudson Canyon
MG3	MGYP000191526608	SRR3963457	MGYS00003225	SRS1589258	ERZ505252	MGYA00237664	103.31, -60.157	-30.33, 43.858	Marine microbial communities from the Deep Indian Ocean - MP1202
MG4	MGYP000119647116	SRR3933371	MGYS00002420	SRS1572596	ERZ490325	MGYA00514313	-60.157	39.54	Hibernia:Winter (seawater metagenome assembly)
MG5	MGYP000202667312	SRR5469068, SRR5469034, SRR5469066	MGYS00003548, MGYS00003322, MGYS00003323	SRS2139748, SRS2139712, SRS2139745	ERZ511469, ERZ511465, ERZ511466	MGYA00248564, MGYA00237859, MGYA00237860	-72.2, -72.2, -72.2	39.29, 39.29, 39.55	Methane-oxidizing microbial communities from mesocosms in the Hudson Canyon - EN10B, EN8C, EN8B Hudson Canyon
MG6	MGYP000668578634	SRR5469033, SRR5469068	MGYS00003337, MGYS00003548	SRS2139711, SRS2139748	ERZ534229, ERZ511469	MGYA00237882, MGYA00248564	-72.4, -72.4	39.54	Methane-oxidizing microbial communities from mesocosms in the Hudson Canyon - EN1E, EN10B Hudson Canyon
MG7	MGYP000096518708	SRR5469033	MGYS00003337	SRS2139711	ERZ534229	MGYA00237882	-72.4	39.55	Methane-oxidizing microbial communities from mesocosms in the Hudson Canyon - EN1E Hudson Canyon
MG8	MGYP000532440779	SRR3735442	MGYS00003486	SRS1533932	ERZ501120	MGYA00245329	N/A	N/A	CA23DSaliva (human oral metagenome)
MG9	MGYP000333245184	ERR527073	MGYS00002338	ERS475133	ERZ485674	MGYA00156231	120	30	Healthy samples for discovery (human gut stool)
MG10	MGYP000049978080	SRR1950715	MGYS00005037	SRS892079	ERZ501064	MGYA00248913	N/A	N/A	Human skin metagenome

Table S3. Percent sequence similarities of MG1-MG10 enzymes compared to known PET hydrolases. Pairwise amino-acid alignments were performed using the EMBOSS-Needle method.

	BhrPETas	BsEstB	Cbotu_Est.	Cut190	Est119	Est1	FsC	HiC	IsPETase	LCC	PE-H	PET12	PET2	PET5	PET6	PHL_7	Tcur0390	Tcur1278	TfCut2	TfH	Tha_Cut1	Thc_Cut1	Thc_Cut2	Thf42_Cut1	Thh_Est
**MG1**	57.9	18.1	21.9	56.1	58.9	60.4	24.2	12	61.7	59.2	69.7	58.8	69.3	74.2	72.5	55	58.5	59.2	60.6	62.7	59.7	60.6	58.4	60	55.5
**MG2**	57.2	22.5	19.4	56.3	59.4	59.7	15.7	16.6	67.2	58.8	72	65.7	72.4	85.8	66.3	54.5	57.1	60.5	57.2	60	56.3	57.2	55.9	56.9	57.6
**MG3**	60.3	22.3	22.3	56.6	59.4	59.8	17.8	14	67.6	61.5	70.4	66.3	70.1	72.6	71.4	56.1	59.3	61.5	61.8	63.8	60.8	61.8	59.9	61.2	58.9
**MG4**	47.5	19.1	25.1	45.8	50.8	52.2	13.7	22.4	56.8	49.9	53.2	50.1	54.2	55.6	51.3	44.9	48.8	47.4	47.2	50.8	46.4	47.2	46.4	46.9	46.6
**MG5**	58.3	19.8	26.5	54.9	58.8	60.1	16.8	22.4	66.7	58.6	71.5	65.7	71	86.5	65.4	55.1	56.4	59.7	57.9	61	57.6	58.5	57.2	58.5	57.9
**MG6**	56.2	22.6	20.3	54.8	58.6	59.6	17.1	14.6	65.5	58.1	70.6	65.5	71.7	85.2	65.8	54.9	56.7	59.1	57.1	59.3	56.1	57.1	55.4	56.7	57.3
**MG7**	58.1	17.3	27.2	56.5	58.8	59.7	17.2	15.2	64.8	58.3	70.4	65.4	72.8	86.5	65.7	54.8	57.6	60.8	56.9	59.8	55.9	56.9	55.6	56.9	57.2
**MG8**	53.6	21.3	21.8	56.3	56	58.2	15	16.6	63.9	55	81	64.1	70.8	69.5	65.3	55.6	59	60.8	55.8	58.5	56.1	56.8	55.8	56.8	58.8
**MG9**	57.3	21	19.2	66.9	63.5	64.6	25.2	22	58.7	58.2	56.7	58.9	55.3	54	51.1	64.7	66.8	67.5	61.9	65.8	60.9	61.9	61.2	61.9	62.8
**MG10**	67.4	23.1	18.3	67.9	68.5	70.2	22.8	17.8	61.8	67.3	61.4	64.6	57.2	58.9	55.8	67.2	73.8	73.4	66.2	70.3	64.8	65.9	65.9	66.6	66.2

Table S4. Sequences of putative PET-degrading enzymes and GFP1-10. Sequences were codon-optimized for recombinant expression in *E. coli*. Genomic sequences can be obtained from the provided MGnify IDs in Table S2.

Gene name	Gene sequence
MG1	ATGAATACATACTTACTTCGTACACTGAGTATCTGTTTATTCGCAGGGCTTTTTAGTATG<br>CAGGTGCAGGCTATTACGCCCGACCCGGAGCCGGATCCGGACCCCGATCCACCTAGC<br>ACTTGCTCCAACTGCTATCAACGTGGACCAAATCCGACGGTCAGCGCATTAGAGGCTG<br>ACAGCGGGCCATACAGCGTTCGCACTATCAATGTCAGCTCATGGGTATCGGGCTTCGG<br>GGGAGGCACTATTCATTATCCAGTCGGAACAGAGGGAACTATGGGGGCTATCGCAGTT<br>ATTCCAGGCTATGTGAGCTATGAGCGTAGCATCAAGTGGTGGGGACCTCGTTTGGCAT<br>CGTGGGGTTTCGTTGTCATCACAACAGATACTAACACCATCTACGACCAGCCTGACTCA<br>CGCGCCGACCAATTATCCGCGGCCTTAGATTACGTTATTAGCCAGTCGAATTCTAGTCG<br>TTCACCGATTTATGGCATGGTCGATGCTAATCGCCTTGGGGCGATGGGGTGGAGTATG<br>GGAGGTGGTGGAACATTGAAACTTAGCACCGAACGCGAGCTTAAAGCGGCGATTCCGC<br>AAGCACCTTATTATGCTGGGTTCAACCCTTTTGATGAGATTACGACCCCGACCTTAATTA<br>TCGCTTGTGAACTGGACGTTGTCGCTCCCGTGGCCCAACATGCTAGTCCTTTTTACCGT<br>GAAATCCCCGGATCTACTGCAAAAGCGTTCCTTGAGATCAACGGTGGGGACCACTTCT<br>GTGCAAACTCCGGATATCCCGACGAAGATATCTTGGGAAAATACGGCATCGCGTGGAT<br>GAAACGTTTTATTGATGAGGATCGTCGTTATGACCAATTCTTGTGTGGTCCAAATCATGA<br>AGCAGACCGCTCCATTTCGGAATACCGCGACACGTGTAATTAC
MG2	ATGAATAAATCTATTCTTGAGAAAATGGTAGTAGGCACATCTGCATTATTGTTTTCTCTTA<br>GCGCATTCTCTTTCACCCCAGATCCGACCCCTGACCCCGATCCCGAACCTGAGCCATG<br>TCAGGGCGAATGCGACTTCACTCGCGGTCCCGCGCCCACTTCTTCTTACCTTGAGGCG<br>TCGTCAGGCCCGTACAGTGTTGATACAGTAGGCGTGTCTCGCTCTGTTTCCGGCTTCG<br>GCGGCGGTACCATTTATTATCCTACAAGCACGAGCGGAACAATGGGTGCCATTGCTATT<br>GTCCCGGGTTTCTTGGCGGGGGAATCATCTATTTCTTGGTGGGGTCCCCGTCTGGCGT<br>CCCACGGCTTCGTGGTTATCACTATTGCGACCAACAGCACCTTCGATCAACCCGCAAG<br>TCGTTCAACTCAGCTGTCTCGTGCCTTAGATTACGTTGTGTCGCAAAGTAATTCTAACA<br>GTTCTCCAATTTCGGGCAAAGTTGACTCCACCCGCCTGGGTGCAATGGGATGGAGCAT<br>GGGTGGAGGTGGCGCTCTGCGTCTGGCAAGCGGCAATCGCTTAGCTGCTGCGATCCC<br>TCTTGCACCTTGGAACACGGGCAGTAACAGTTTTAACCAGATCGATACACCCACCTTGA<br>TCTTTGCCTGCGAGAATGACACGACCGCACGCGTCGGCACTCACGCGTCCCCGTTCTA<br>TAATCGTATCCCAGCATCTACAGACAAAGCGTACCTGGAGATCAACAACGGTTCGCACT<br>CATGTGCGAACGGCGGAGGGTCCAATGGAGGTTTACTTGGCAAATACGGGGTTTCTTG<br>GATGAAGCGTTTTATCGATGAAGACACGCGCTATTCTCAATTTTTATGCGGACCAAATC<br>ACGAGGCAAACAGCGCAATCTCTGAGTACCGTGAGACCTGTAACTAC
MG3	ATGAAAACTGTGCGTTTTAACACGGCAGCTGCCGTCTTTACCTCTGCCCTTCTTTCTTCA<br>CAGGTATTCGCTATCACCGACGACCCAGTTGACCCTGTAGACCCCGTAGATCCGCCTT<br>CGTCAGGTACTGTACGTGGACCGGACCCGACACTTTCTGCGCTTGAGAGCACGCGTAG<br>TGGTCCCTATTCTGTGCGTACCGAGAATGTGTCTAACTTGAGTGCCTCCGGCTTTGGC<br>GGCGGGACAATCCACTACCCTACGAACGCTGGGGAGAATATGGGAGCTATTGCGGTTA<br>TTCCCGGGTACGTCAGTTACGAATCGAGCATCGAGTGGTGGGGTCCACGTTTAGCGTC<br>TTGGGGGTTCGTAGTAATCACGATTGACACAAATACAATCTACGACCAGCCGGATTCGC<br>GCGCGGATCAGCTGAGTGCGGCCCTTGATCACCTGATTGATGAAAGTGGAAGCAGCTC<br>TAGCCCGATTTCTGGTTTAGTCGATGCTTCGCGCTTAGGTGTGATCGGATGGAGTATG<br>GGCGGGGGTGGTACTCTTAAGCTGGCCACGGAACGCAACCTGAAAGCGATTATCCCA<br>CAAGCACCCTGGTATAGTGGCTTCAATTCGTTCGACCGTATCACTACACCCACAATGAT<br>CATCGCCTGCGAATCCGACGCGATTGCGCCCGTAGGGCAGCACGCTTCCCCGTTTTAC<br>AATGATATTCCGAATTCTACTGCGAAAGCCTTTTTGGAAATTAATGGAGGTTCACACTAC<br>TGCGCCAACAGTGGGTATTCCGACGAGGATATCTTGGGAAAGTATGGTATCTCATGGA<br>TGAAACGTTTTATGGACAACGACACTCGCTATTCACAATTTTTATGTGGTCCTAATCATG<br>AGTCTGACCGCTCCATCAGTGAGTACCGCGATACCTGCAATTAT
MG4	ATGTTTGATCCGAACGGAGTGTCCTTTGTTGTAACCGATTCTGACAACGTCGGATTTAA<br>CGGCAATTTCGGTCTTTCGTCTAGTTCATTATTAACGCTGAATTTCATCGACTTCGGTCC<br>AGGCGAAACATTTAGCTTCAACGGGGACCTTGACGACTCGAATGGTTCATACACAACG<br>GGGGCGGACGTTATGGGGGCCGAGTTGACCGTAAGTGCTTCGGGAAATACTTCCGTC<br>AGCGCGTCGTTCACAGTCGATTCATCGAACGCCGATCGCGCAGTGGTTAATATCGGGG<br>GGTCAACTCCACCACCCCCAATTGGGGGAGGATGTGGACCAGATCCCACGTTGGCCT<br>CTCTTGAAGCCTCGACAGGCCCGTTCTCCTTCTCTTCCATTGACGTACCGTCATCAGCA<br>CAAGGCTTTCGCAATGGTACCATCTATTATCCCACAAATGCCCCCGTAGATTGTACTTT<br>CGGTGTAATCTCAGTGGTACCCGGATTCTTGGCTAACGAGAGCTCGATCGACTGGTGG<br>GGACCACGCTTGGCATCCCACGGCTTTATTGTAATCACCATCAATACCAACACGATCAC<br>CGATCAACCAAACTCTCGCGCACTTCAAAGCATTGCCGCCTTAAATTATGCGGTGAACT<br>TAGGTAATTCAAGCAGTAGCGCTATTGGTGGCAAAGTGGACTCCTCAAAACAAGGGGT<br>CATGGGTTGGTCAATGGGCGGAGGTGGAGCGTTACGTGCTGCGGCCGACAACCCTTA<br>CTTGAAGGCCGCGATGCCACAGGCGCCATTTAATTCTGGTCGCAACGACTTTGATGAA<br>ATCACAGTGCCAACCTTAATTATTGCGTGCGAAAATGATACTGTGGCCAGCGTAGGCGT<br>TCACGCCTCCCCCTTCTACAACGCTATCAGTTCGTCGACGGAGAAAGGATTCCTTGAAA<br>TCAACAACGGGAGCCATTCATGTGCGAACGGGGGTTCCAATCGCGACATCCTGGGCAA

GTATGGCGTGAGTTGGATGAAGCGTTTTATGGACGACGATACTCGCTACTCGCAGTTCT
TGTGCGACGTGAATCACACCTCGGATTCGGCCATCTCTGATTATCGCGAGAACTGTCCT
TAT

MG5 ATGAATACTTCTCTGCTTGAAAAAATTGCAATTGGAACGTCGACTCTGTTATTCTCGGTT
TCTGCATTCTCGTTTACACCGTCACCGACACCTGATCCGATTCCCGAGCCCGAGCCAT
GCCAGGGAGAGTGCGACTTCACGCGTGGTCCTTCTCCTACCTCAAGTTATTTAGAAGC
ATCGTCCGGCCCGTACAGCGTGAATACGGTTAATGTTAGTCGCAGCGTGAGCGGGTTC
GGAGGCGGTACGATCCACTATCCGACCAATACTGATGGAACTATGGGTGCCATTGCAA
TTGTACCTGGTTTTTTTGCTGCGGAAAGCTCCATTGCATGGTGGGGACCACGTTTGGC
GAGTCATGGCTTCGTAGTCATTACAATCGCCACAAATTCCACGCTTGACCAGCCAGCGT
CCCGCGCAACACAATTAGATAACGCTCTGGACTACGTTATCTCGCAATCTAATAGTAAT
TCATCGGATATTTCCGGTATGGTTGATTCTAACCGTGTGGGGGTCATGGGCTGGTCGA
TGGGAGGAGGCGGAGCGTTGCGCGTGGCAAGTGGGAATCGTCTGAGTGCAGCTATTC
CGTTGGCGCCCTGGAACACCGGTAGTAATGATTTTGACCAGATCGACACACCCACCAT
GATTATTGCTTGCGAGAATGACAGCACAGCTCCGGTAAACAATCATGCATCACCATTCT
ACAATCGTATGCCCGTCACTACTGACAAAGCATTTCTTGAAATCAACAATGGGGGCCAT
TCATGCGCTAACGGAGGTGGTTCCAACGGCGGGTTGCTGGGCAAATACGGTGTGTCCT
GGATGAAGCGCTTCATTGACCATGATACTCGCTACAATCAGTTCTTATGCGGACCGAAT
CATGCCGCAAACAGTGCCATCAGCGAGTATCGTGAGACTTGTGACTAC

MG6 ATGAATACATCTTTGCTTGAAAAAATTGCCGTAGGCACCAGCGCTTTGTTGTTCAGTGT
CTCGGCCTTTTCTTTCACCCCTAGTCCAACCCCGCCGGTTGATCCGGAGCCTCCAGCA
CCCTGCGAGGGTGAATGCGATTTTACACGTGGCCCTGCGCCAACTAGTAGTTACTTAG
AAGCAAACAGTGGTCCGTATTCTGTAAATACAGTAAACGTATCGCGTTCGGTCAGCGG
GTTTGGTGGAGGAACTATCCACTATCCTACTAATACTGCCGGCACTATGGGAGCTATTG
CCGTAGTCCCTGGATTCCTTGCTGGAGAGTCCTCAATCGCATGGTGGGGGCCGCGTCT
TGCATCACATGGTTTTGTGGTTATCACCATTGCCACCAACTCAACATTTGACCAACCCG
CATCTCGTGAAACCCAGTTAGGGCGCGCACTGGACTACGTAATTAGTCAAAGTAACTC
GTCAAGTAACCCAATTAGTGGAATGGTCGACAGTTCGCGTGTGGGGGCTATGGGTTGG
TCCATGGGTGGTGGGGGGGCATTACGCATTGCTTCCGGAGATCGTTTATCGGCCGCG
ATCCCACTTGCGCCTTGGCATACAGGGTCCAATGATTTCGATGAAATTTCAACCCCGAC
TATGATCATTGCCTGTGAGAATGATTCCACCGCACCAGTCCGTTCTCACGCGTCTCCGT
TTTACAACTCCATCCCTGATACCACTGATAAAGCCTTACTGGAGATTAACAACGGTGGC
CACAGTTGCGCTAATGGCGGCGGTAGCAATGGGGGCTTGCTGGGCAAGTACGGGGTC
AGCTGGATGAAACGTTTCATCGATAATGACACCCGTTATTCTCAGTTCTTATGTGGCCC
TAACCATGCAGCTAACTCGGCTATCTCGGAGTACCGTGAAACTTGCGATTAC

MG7 ATGAATAAGTCGATCCTGGAGAAAATTGCTGTCGGTACCAGCGCGCTGTTGTTCTCCGT
TTCAGCGTTTTCTTTCACACCCAGTCCCACGCCGGACCCAGACCCAGAACCGCAACCG
TGTCAGGGTGAGTGTGACTTTACGCGCGGCCCTGCTCCTTCGTCCTCATACCTTGAGG
CCTCTTCGGGTCCTTACTCCGTAAATACCGTTAATGTAAGCCGCTCCGTGAGCGGCTTT
GGCGGAGGTACTATTCACTACCCAACTAATACTACAGGAACGATGGGTGCAATCGCTAT
TGTCCCCGGTTTTCTTGCGGGTGAGAGCTCAATCGCCTGGTGGGGGCCACGCTTGGC
GTCCCATGGTTTTGTGGTCATCACGATCGCTACAAACAGCACCTTTGACCAGCCTGCGT
CTCGCGAAACTCAGCTGGGGCGCGCGCTGGACTATGTGATTTCCCAGTCTAACAGTAA
CTCTTCGGACATCTCTGGCATGGTAGATTCTACTCGTGTTGGTGCTATGGGGTGGTCCA
TGGGCGGTGGTGGCGCTTTGCGTATTGCGTCCGGTTCGCGTCTTTCAGCCGCCATTCC
CTTAGCGCCCTGGCATACTGGCTCCAATGATTTCGATGAAATCGACACCCCTACAATGA
TTATTGCCTGTGAGAACGATTCAACAGCACCCGTTCGTTCTCACGCATCCCCTTTTTATA
ATTCCATCCCGTCCACCACTGATAAGGCTCTTCTTGAGATCAACAATGGGAGCCATAGT
TGCGCGAACGGTGGGGGGTCAAATGGTGGGCTTCTTGGGAAGTACGGCGTTAGCTGG
ATGAAGCGCTTCATTGACGAGGACACTCGTTACAGTCAATTCCTTTGCGGACCCAACCA
TGCGTCTAACTCCGCTATTTCCGAATACCGTGAGACGTGTGACTAT

MG8 ATGAAAGCGCTGACGTTTTCAAAAAGTTTTCTGTCAGCAATCGCAGCGGGTGCCCTGAT
GCTGAGCGCGAGCGCCATGGCGAATAATCCTCCTCCACCAGACGACCCGGGAGCTCC
CAGCCCCTATCAGCGTGGACCGGACCCTACTGTGTCATTCGTGGAAGCAAGCCGCGG
CAACTATCGTGTTGCGACGTCGAACGTCTCCTCTCTCGTGTCGGGCTTTGGTGGCGGT
ACCATTCATTACCCTAGCAATGCCACCGGTACAATGGGTGCGATCGTAGTTATCCCGG
GCTATGTGTCTGGCGAAGGCTCCATTGACTGGTGGGGCCCGAAACTGGCGTCCTACG
GGTTTGTTGTGATGACGATTGGTACCAATTCTGGGTTTGACCAACCACCGTCCCGCGCT
CGTCAAATTAATAATGCATTAGATTACCTGGTGGAGCAGAATACCCGCACAGGCTCGCC
AGTCCGTGGTATGATTGATCCGAGCCGCCTCGGCGTGATCGGCTGGAGCATGGGTGG
CGGTGGTACTATCCGCGTTGCCGGCGAAGGTCGCATCAAAGCCGCCATTCCCCTTGC
GCCCTGGGATACGAGCTCCATCCCCTCGCGTGGCGTGCAAGCACCGACTCTCATCTTC
GCGTGCCAGTCAGATGTCGTTGCGCCGGTGCGCAGTCATGCGTCGCCGTTCTATAACG
CCCTGCCTGGCAGCATTTCCAAAGCATTTGTTAACCTGAATAACGGCAATCACTTTTGT
GCGAACGGTGGGTCTAGCTTCGGACGTTACGACGCCGCGTTGGGCCGTTTGGGCGTT
AGCTGGATGAAACGCTTTCTCGATGAAGATCGTCGCTATAGCCAGTTTTTGTGCGGTCC
CAACCATACTGGTGATCGCCAAATTACGGAGTACCGTGGTAATTGTCCGTAC

MG9 ATGCGCGCAACAGCTACGCCGTCGCGTCTGCGTCGCGCCGTCGTGATTACCACGACC
GCGATCGGCCTGTTAGCAGGCACCGCGCCGCTGGCGCAAAGCGCGGAAACCACAACA
CAGCATGGGCCCGATCCCACCGAAGGCTCTATCACGGCGCCGACGGGGCCGTTTCAG
ACTGACCAGAAAACCGTACCGCGCTCGAGTGTGCAAGGCTTTGGCGGTGGCACCATCT

	ATTACCCTACTGAAACTAGCCAAGGCACATTTGGCGCCGTAACCATTGCTCCGGGCTA<br>CACCGCGGGTAAAGAATCTCTTGCCTATGATGGCTACACCGGAGACCAAAGCTCCATG<br>GCCTGGTATGGTCCGCGTCTCGCTTCTCAGGGTTTTGTCGTTTTTACCATTGACACGAT<br>CACAACGAGTGACCAGCCCGACAGTCGCGCAAAACAGCTGATGGCTTCTCTGGACTAC<br>TTAACCGGCGACTCGGATGTTCGCGATCGTATCGACACGTCCCGTCTGGCCGTCATGG<br>GTCACTCCATGGGCGGAGGTGGTACCCTGGAAGCCGCTCGTGATAACCGTAACCTGAA<br>AGCGGCGATTCCAATGACCCCGTGGGATACCACCAAAGATTTCTCCGGGGTGCAAACC<br>CCCACGCTGATTATCGGGGCACAAAATGATACCATTGCGCCCGTTGCCCAACACGCGA<br>AACCATTTTATGGTAGTCTGCCGGATGATCCAGGCAAAGCGTATCTGGAGCTGGCCGG<br>CGCGTCTCACTTTGCACCCAACCAGGACAATACGACCATTGCGAAATCCTCCATTGCCT<br>GGTTGAAGCGTTTTGTCGATGATGACACCCGTTACGATCAATTTTTGTGTCCACCGCCG<br>CAGGATGCCGAGATCTCAGATTACCAGTCAACATGTCCTTAT
MG10	ATGCCAACCCCGCGGCTGCTGACCCGTGCGCTTGCTGCATTAGCGCTGGCCTTGGCA<br>ACCCTGGTCGCGCTTTCCATTCCGGCTAGTGCCGATCCAAGCTGGTGGCGTGGGCCG<br>GCGCCGACACAGGCGGGGATTAGTGCAGAACGCGGCCCATTTGCGGTTGCAAGTACA<br>AGCGTGTCTGATTTCAGCACGCGCGGATTTGGGTCCGCGACAATTCATTACCCTACGA<br>CCACGACGGCGGGGACCTTCGGCGGTGTGGCAATTTCACCGGGTTATACCGCTTCGG<br>AATCAACCATCGCATGGTTAGGACCGCGTATTGCCAGCCAGGGTTTTGTGGTGATTAC<br>CTTCAACACAAATAGCCGTTATGACCAGCCAGCAAGTCGGGGGGATCAGCTGCTGGCG<br>GCACTGGATTACCTGACCGGCAGCTCTGCGGTGCGCGCGCGTGTTGACGCCTCACGC<br>CTGGCGGTGATGGGTCATTCAATGGGCGGTGGTGGCGCGCTGGAAGCAGCCAAGGAC<br>CGTCCGTCCCTGCAGGCTGCGATCCCTCTGACGGGCTGGAATACCGACAAAACGTGG<br>CCGGAAGTGCGCACGCCGACACTGGTCATTGGCGCAGAAAATGATTCTGTAGCGCCA<br>GTGGGCTCCCACTCCATCCCCTTTTACAATAGCCTGACGTCTGCGCCCGAACGGGCAT<br>ACCTGGAACTGAACAACGCGTCGCATTTTGCGCCGAACTCGCCGAATACGGATATCGC<br>GAGCAGCAGCATCGCGTGGCTGAAGCGCTATGTGGACGATGATACGCGTTACACGCA<br>ATTTTTATGCCCGGGCCCGAGTGTCTCGGCCTTCGGCTCGGTATCAGATTACCGCGCT<br>ACCTGTCCGATG
GFP 1-10	ATGAGCAAAGGAGAAGAACTTTTCACTGGAGTTGTCCCAATTCTTGTTGAATTAGATGG<br>TGATGTTAATGGGCACAAATTTTCTGTCAGAGGAGAGGGTGAAGGTGATGCTACAATCG<br>GAAAACTCACCCTTAAATTTATTTGCACTACTGGAAAACTACCTGTTCCATGGCCAACAC<br>TTGTCACTACTCTGACCTATGGTGTTCAATGCTTTTCCCGTTATCCGGATCACATGAAAA<br>GGCATGACTTTTTCAAGAGTGCCATGCCCGAAGGTTATGTACAGGAACGCACTATATCT<br>TTCAAAGATGACGGGAAATACAAGACGCGTGCTGTAGTCAAGTTTGAAGGTGATACCCT<br>TGTTAATCGTATCGAGTTAAAGGGTACTGATTTTAAAGAAGATGGAAACATTCTCGGACA<br>CAAACTCGAGTACAACTTTAACTCACACAATGTATACATCACGGCAGACAAACAAAAGA<br>ATGGAATCAAAGCTAACTTCACAGTTCGCCACAACGTTGAAGATGGTTCCGTTCAACTA<br>GCAGACCATTATCAACAAAATACTCCAATTGGCGATGGCCCTGTCCTTTTACCAGACAA<br>CCATTACCTGTCGACACAAACTGTCCTTTCGAAAGATCCCAACGAAAAGTAA

Table S5. Primer sequences for cloning.

Primer name	Primer sequence
MG8-F250-R	GTGATTGCCGTTATTCAGGTTAACAAATGC
MG8-F250A-F	TGAATAACGGCAATCACGCCTGTGCGAACGGTG
MG8-F250I-F	TGAATAACGGCAATCACATTTGTGCGAACGGTG
MG8-F250L-F	TGAATAACGGCAATCACCTGTGTGCGAACGGTG
MG8-F250S-F	TGAATAACGGCAATCACAGCTGTGCGAACGGTG
MG8-F250V-F	TGAATAACGGCAATCACGTGTGTGCGAACGGTG
MG8-RYD-del-F	TGGGTCTAGCTTCGGAGCCGCGTTGGGCCGTT
MG8-RYD-del-R	TCCGAAGCTAGACCCACCGTTCGCACA
IsPETase-RYD-ins-F	CTGGGAACAGCAACCAGCGTTATGATGCACTGATCGGAAAAAAAGGGG
IsPETase-RYD-ins-R	CTGGTTGCTGTTCCCAGAGTTGGC
MG8-RYD- check-F	TGCCTGGCAGCATTTCCAAAG
MG8-RYD- check-R	GGTTGGGACCGCACAAAAAC
IsPETase-RYD-check-F	AATTAACGGCGGTAGCCACTC
BB-MBP-F	TACATATGAAATCTTCTCACCATCACCAT
MBP-MG8-R	TGCATTGGATTGGAAGTACAGGTTTTC
MBP-MG8-F	AACCTGTACTTCCAATCCAATGCAAATAATCCTCCTCCACCAGACGAC
MG8-BB-R	AGGATCCTTAATTAACTAGCTGGTGCTACCCCCCCC

^{1}H and ^{13}C NMR characterization of pDAP and its synthetic precursors

^{1}H NMR

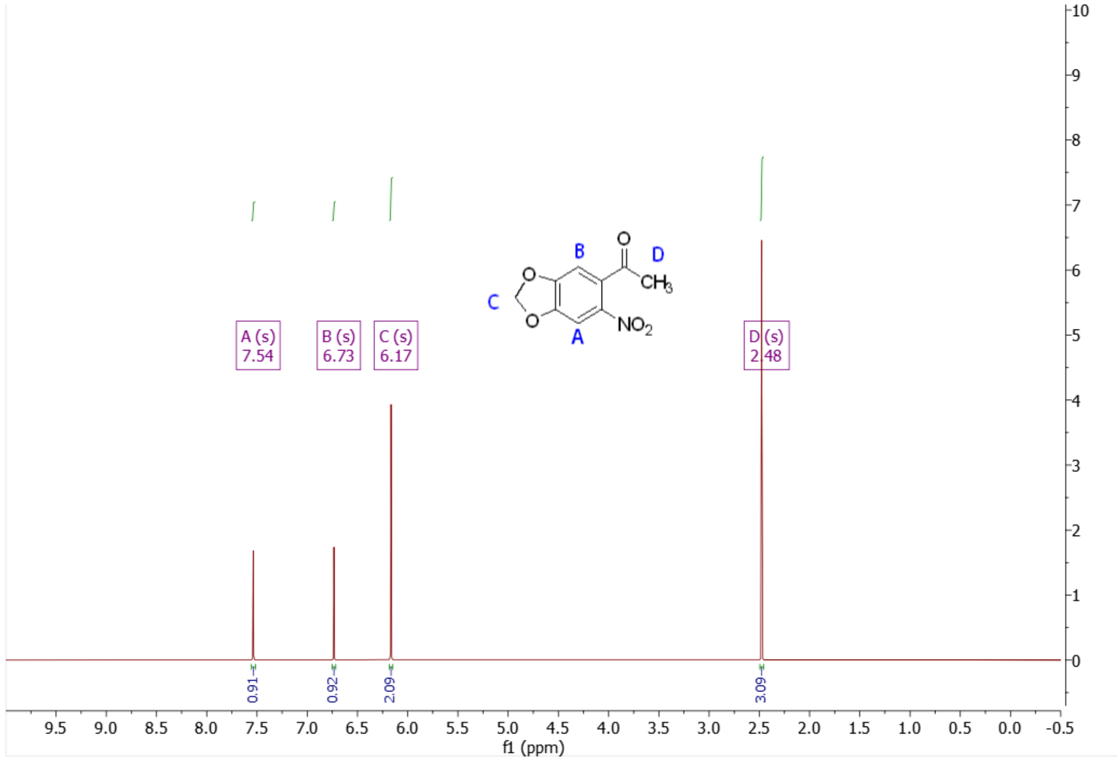

^{13}C NMR

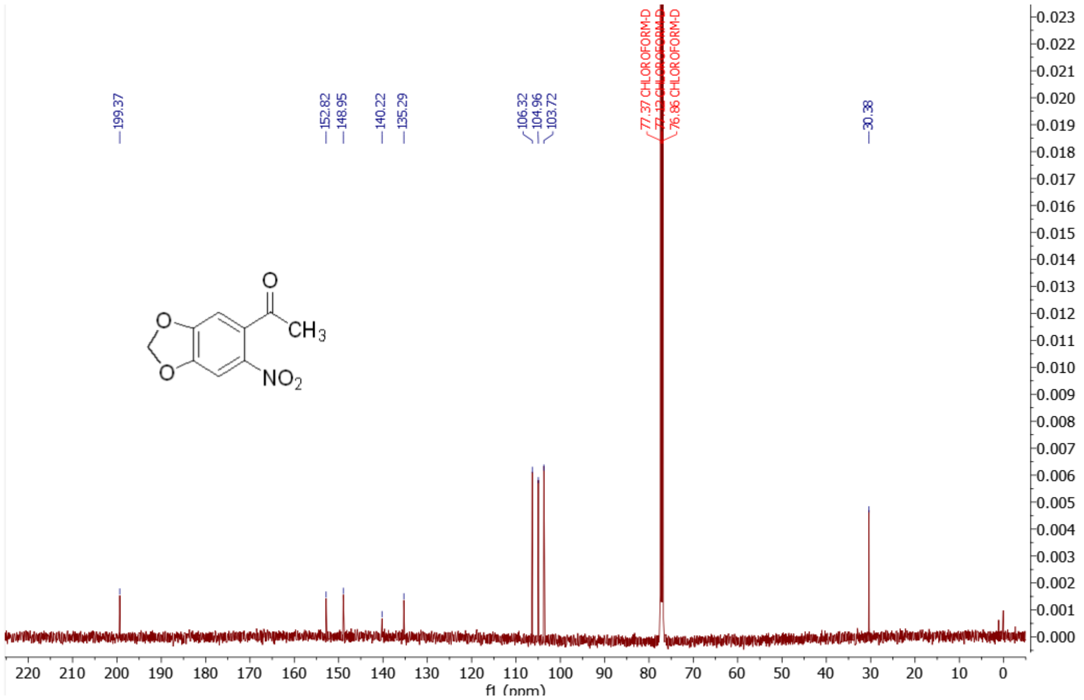

^{1}H NMR

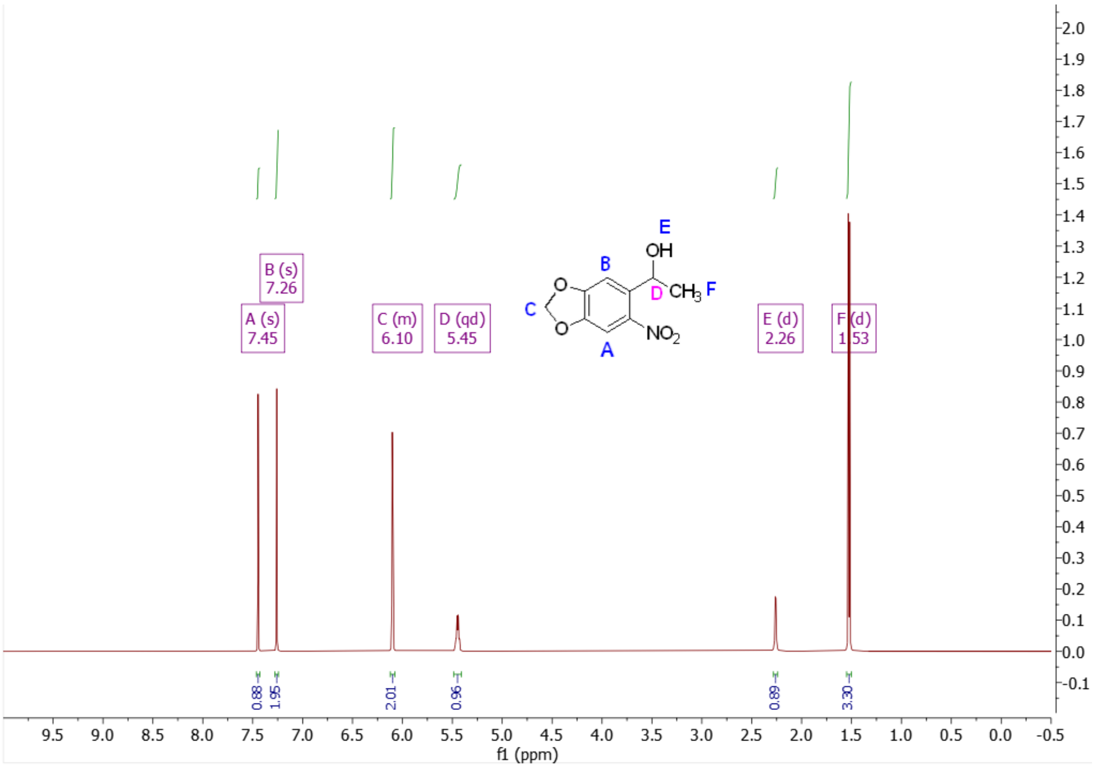

^{13}C NMR

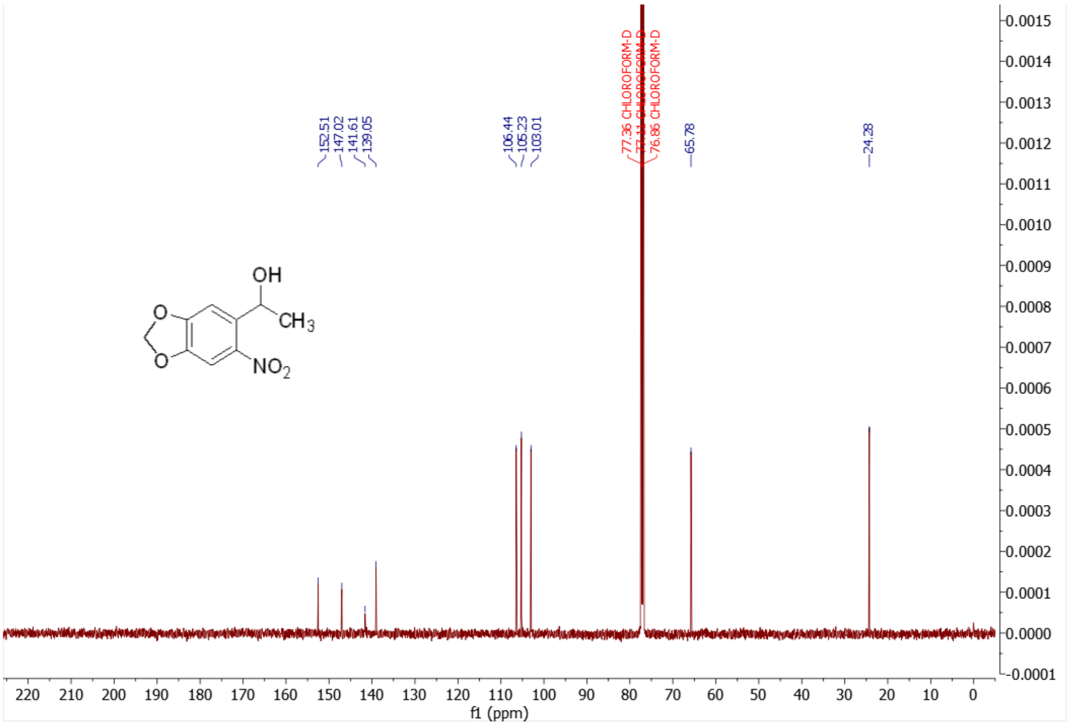

^{1}H NMR

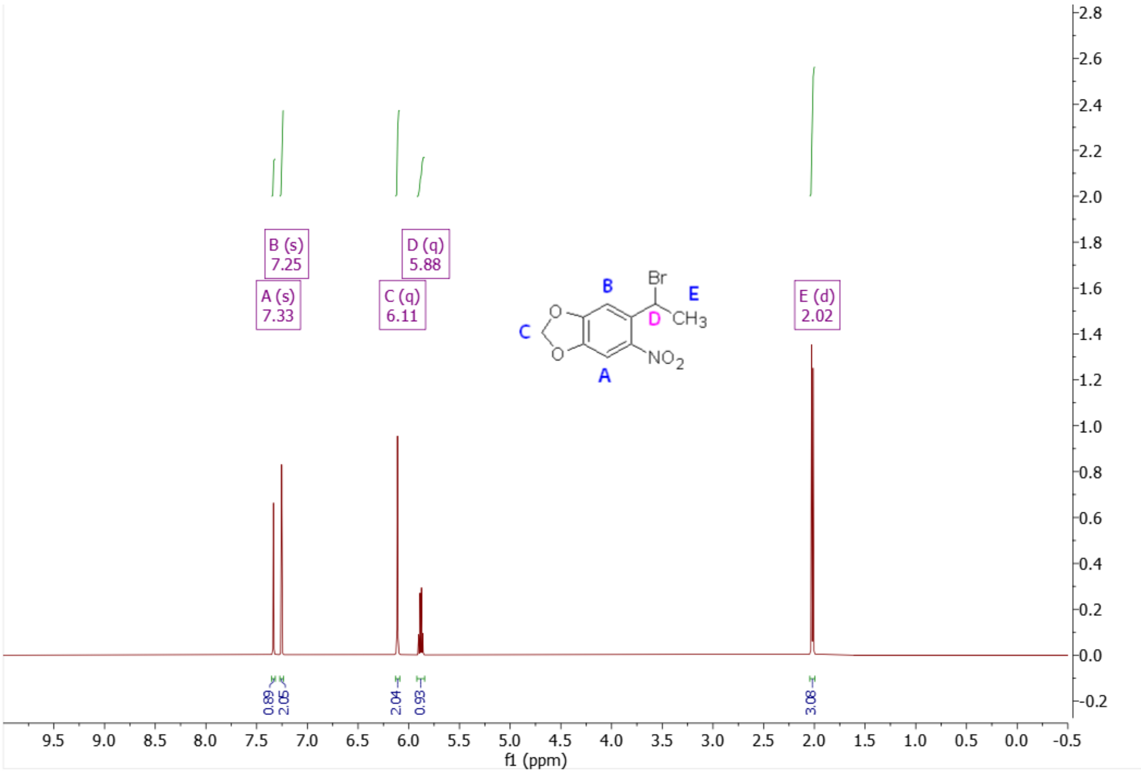

^{13}C NMR

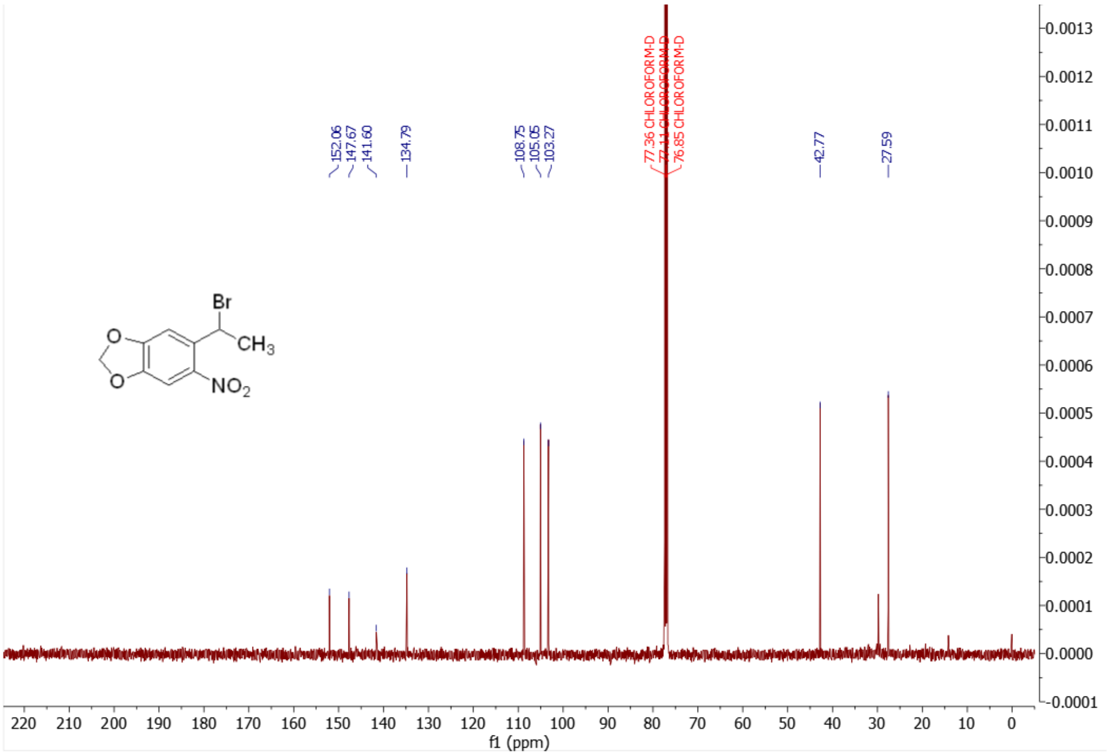

^{1}H NMR

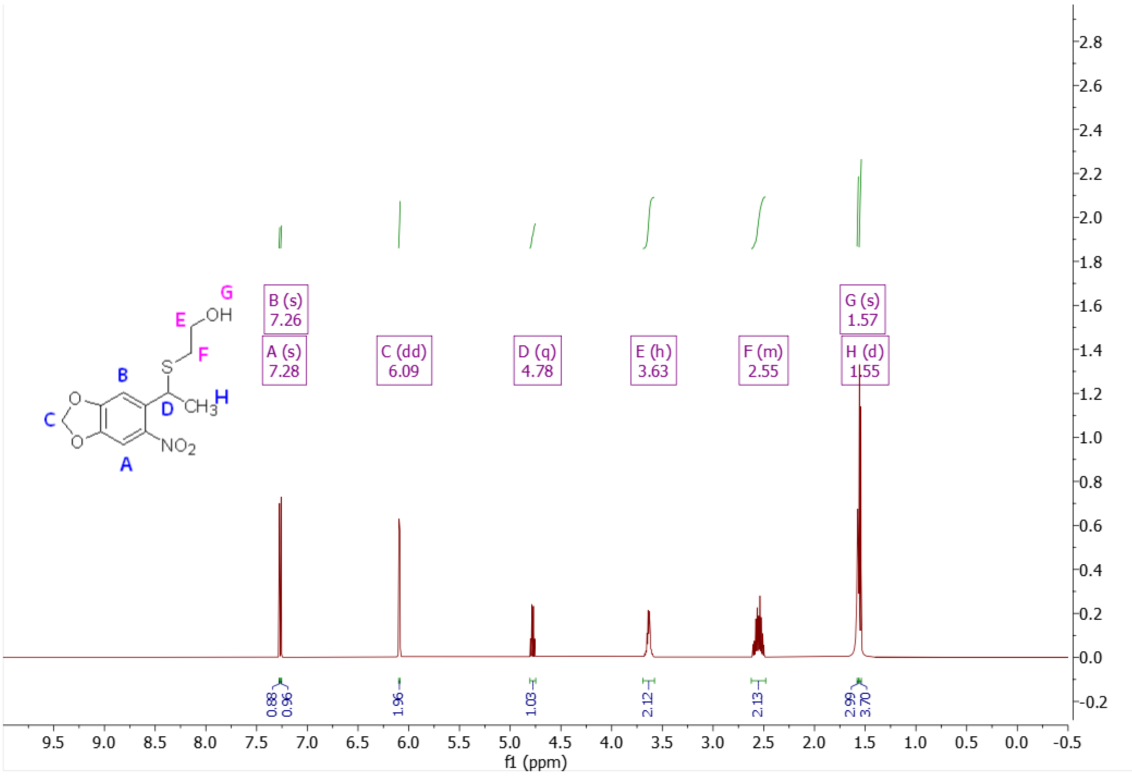

^{13}C NMR

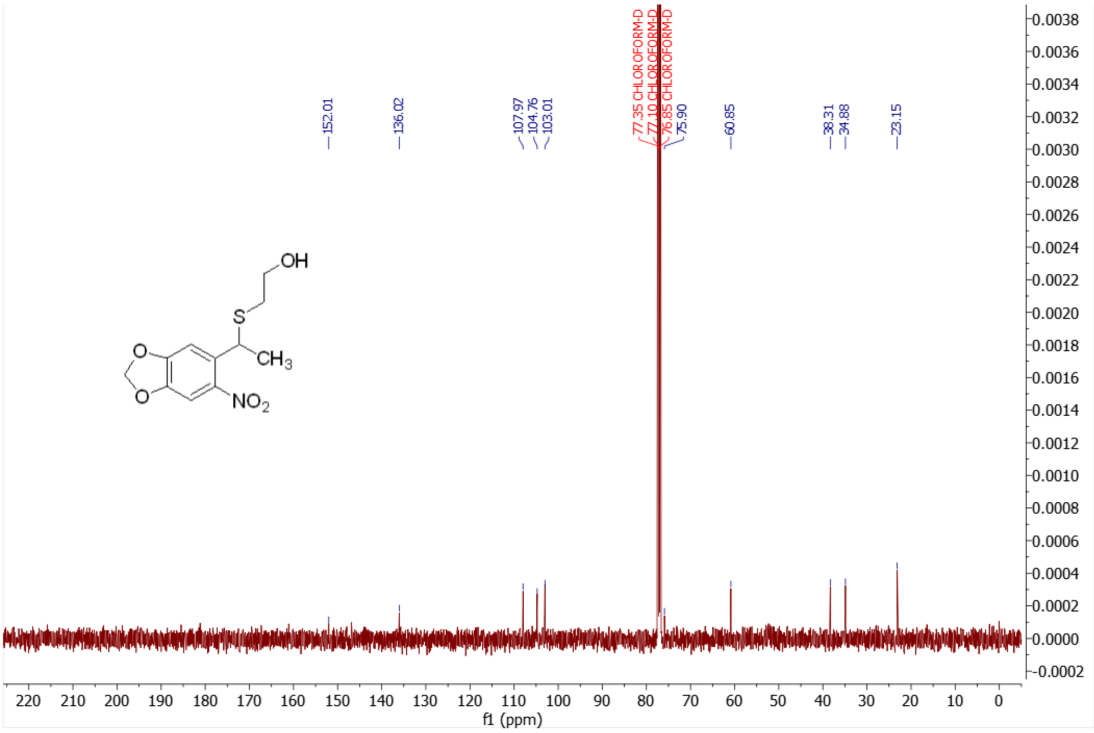

^1H NMR

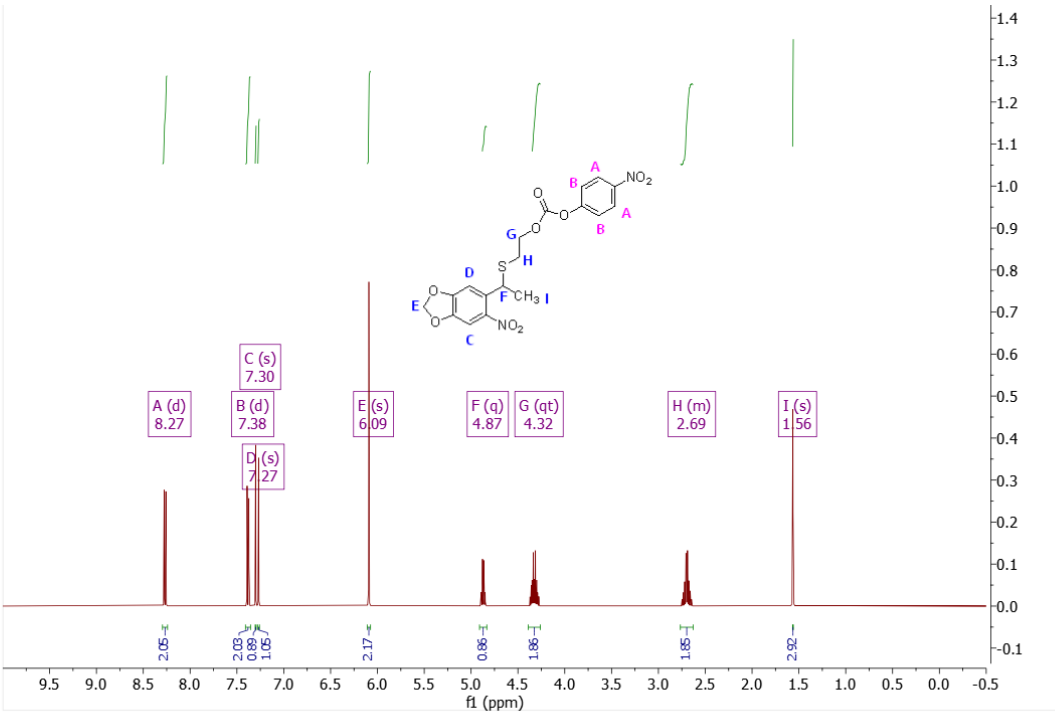

^{13}C NMR

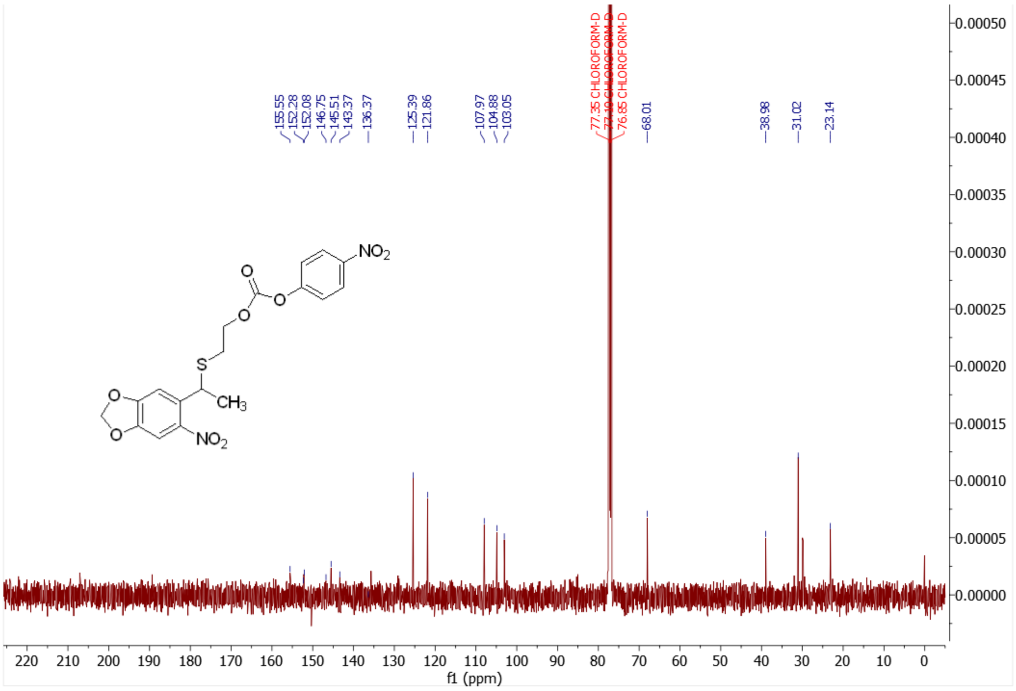

^{1}H NMR

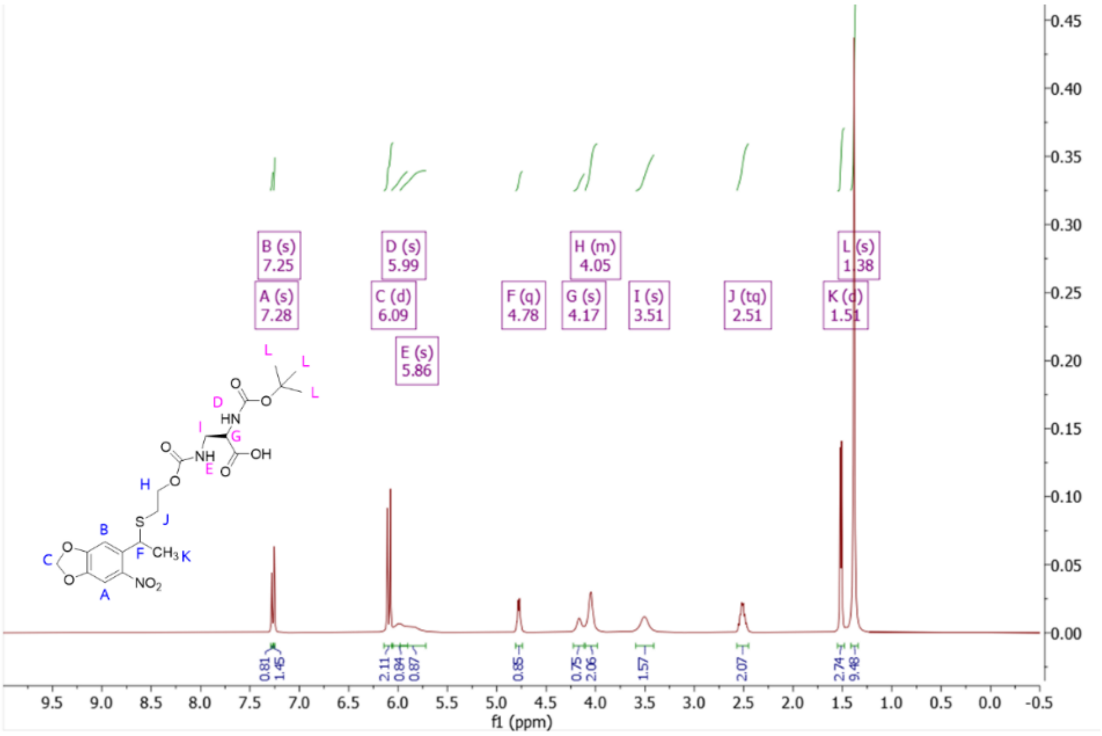

^{13}C NMR

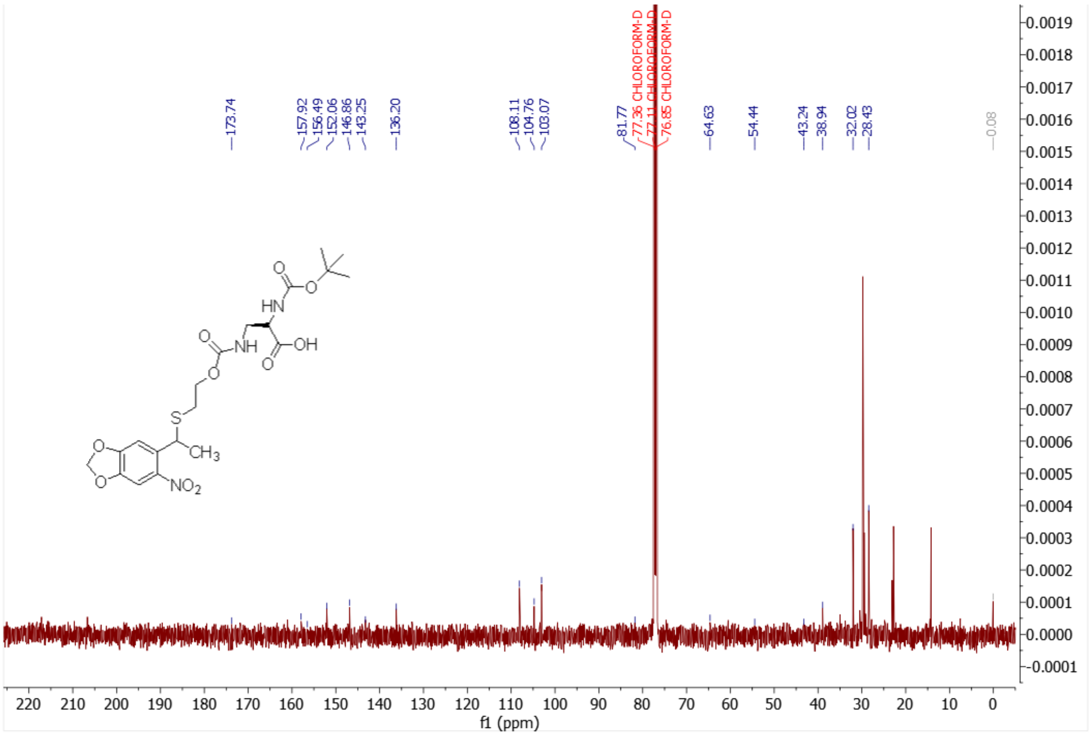

^{1}H NMR

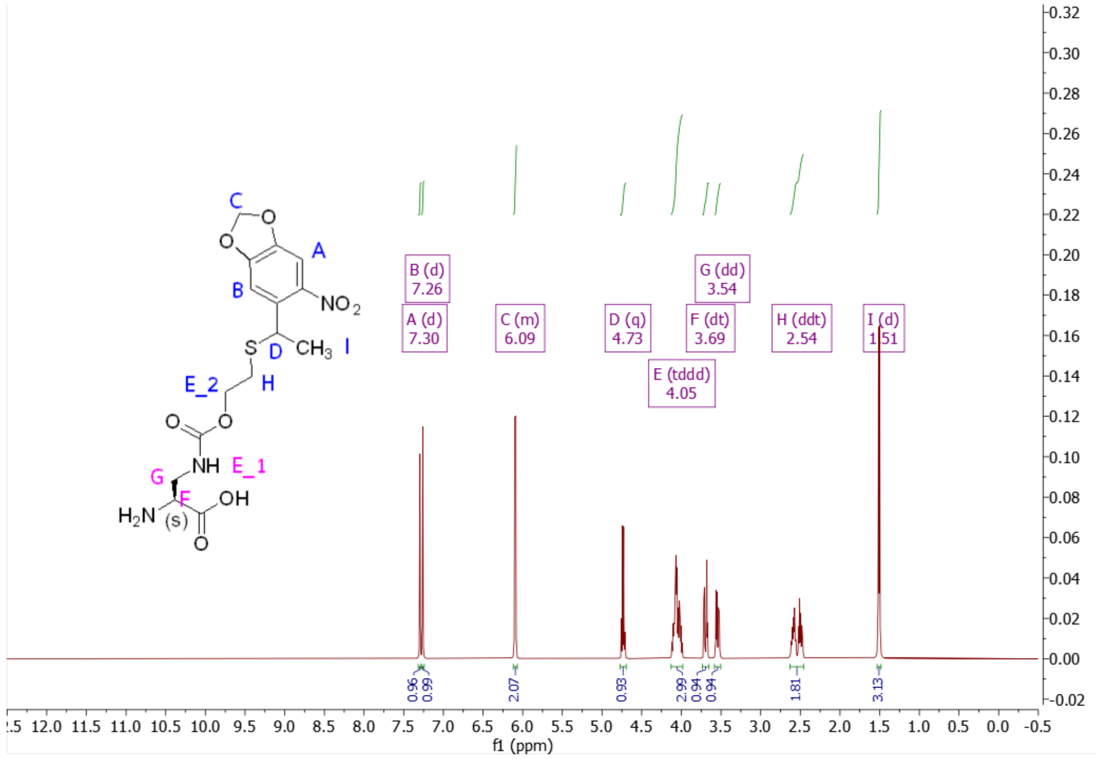

^{13}C NMR DEPT 135

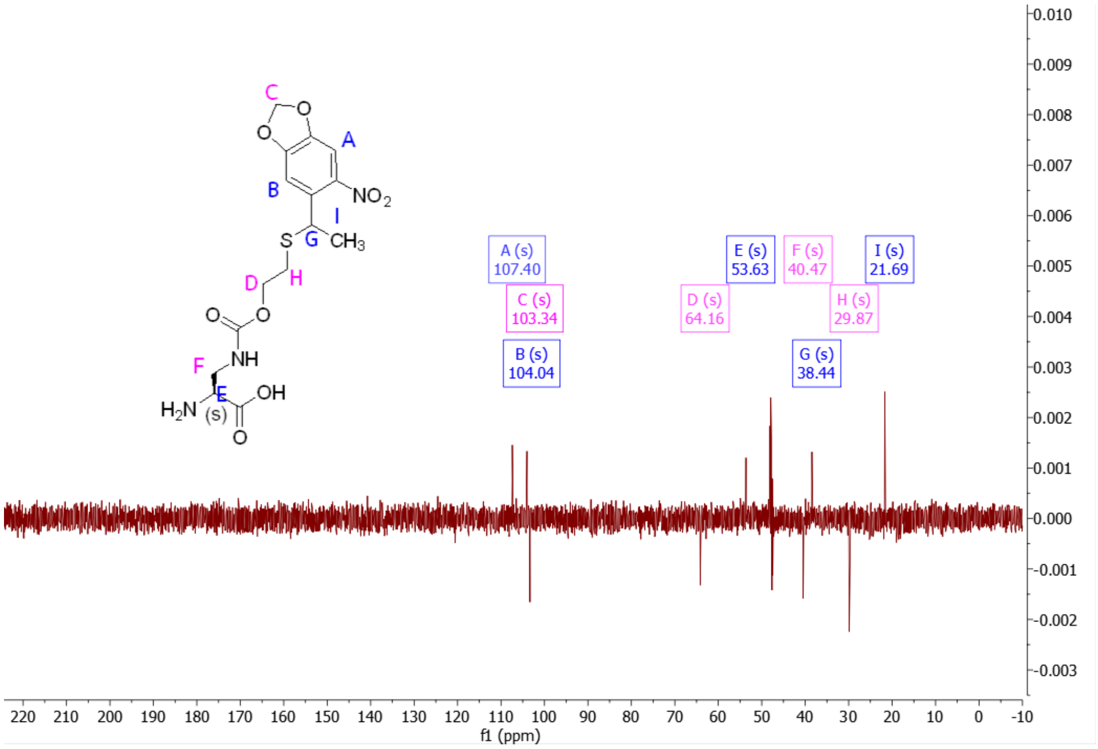

^{13}C NMR

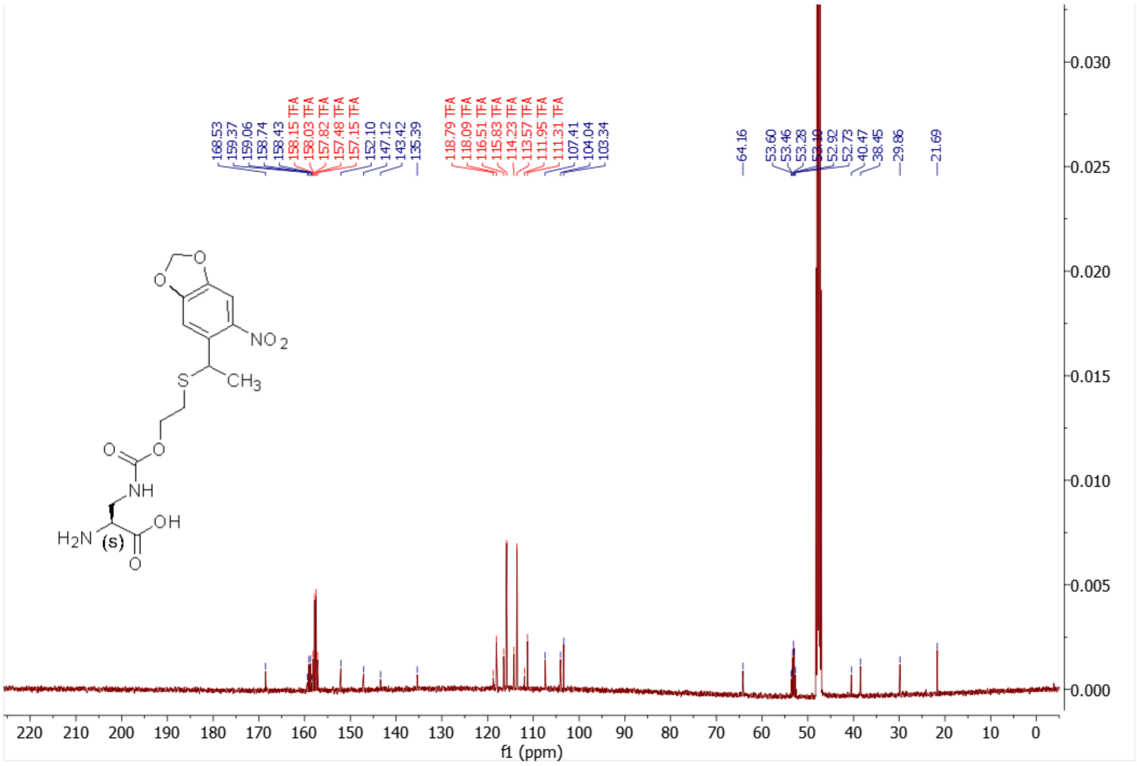

Supplementary References

[1] L. J. McGuffin, K. Bryson, D. T. Jones, *Bioinformatics* **2000**, *16*, 404-405.
[2] J. J. Almagro Armenteros, K. D. Tsirigos, C. K. Sønderby, T. N. Petersen, O. Winther, S. Brunak, G. von Heijne, H. Nielsen, *Nat Biotechnol* **2019**, *37*, 420-423.
[3] S. Joo, I. J. Cho, H. Seo, H. F. Son, H.-Y. Sagong, T. J. Shin, S. Y. Choi, S. Y. Lee, K.-J. Kim, *Nature Communications* **2018**, *9*, 382.
[4] M. J. R. f. Toledo, **2013**.
[5] N. Huguenin-Dezot, D. A. Alonzo, G. W. Heberlig, M. Mahesh, D. P. Nguyen, M. H. Dornan, C. N. Boddy, T. M. Schmeing, J. W. Chin, *Nature* **2019**, *565*, 112-117.
[6] A. Waterhouse, M. Bertoni, S. Bienert, G. Studer, G. Tauriello, R. Gumienny, F. T. Heer, T. A. P. de Beer, C. Rempfer, L. Bordoli, R. Lepore, T. Schwede, *Nucleic Acids Res* **2018**, *46*, W296-w303.
[7] E. Jurrus, D. Engel, K. Star, K. Monson, J. Brandi, L. E. Felberg, D. H. Brookes, L. Wilson, J. Chen, K. Liles, M. Chun, P. Li, D. W. Gohara, T. Dolinsky, R. Konecny, D. R. Koes, J. E. Nielsen, T. Head-Gordon, W. Geng, R. Krasny, G. W. Wei, M. J. Holst, J. A. McCammon, N. A. Baker, *Protein Sci* **2018**, *27*, 112-128.
[8] E. F. Pettersen, T. D. Goddard, C. C. Huang, E. C. Meng, G. S. Couch, T. I. Croll, J. H. Morris, T. E. Ferrin, *Protein Sci* **2021**, *30*, 70-82.
[9] J. Santos-Aberturas, M. Dörr, U. T. Bornscheuer, *Methods Mol Biol* **2018**, *1685*, 157-170.
[10] M. Furukawa, N. Kawakami, K. Oda, K. J. C. Miyamoto, **2018**, *11*, 4018-4025.
